# Robust and synthesizable photocatalysts for CO_2_ reduction: a data-driven materials discovery

**DOI:** 10.1038/s41467-019-08356-1

**Published:** 2019-01-25

**Authors:** Arunima K. Singh, Joseph H. Montoya, John M. Gregoire, Kristin A. Persson

**Affiliations:** 1 Joint Center for Artificial Photosynthesis, Lawrence Berkeley National Laboratory, Berkeley, CA 94720 USA; 2 Chemical Sciences Division, Lawrence Berkeley National Laboratory, Berkeley, CA 94720 USA; 3 Environmental Energy Technologies Division, Lawrence Berkeley National Laboratory, Berkeley, CA 94720 USA; 4 Joint Center for Artificial Photosynthesis, California Institute of Technology, Pasadena, CA 91125 USA; 5 Department of Materials Science and Engineering, University of California, Berkeley, CA 94720 USA

## Abstract

The photocatalytic conversion of the greenhouse gas CO_2_ to chemical fuels such as hydrocarbons and alcohols continues to be a promising technology for renewable generation of energy. Major advancements have been made in improving the efficiencies and product selectiveness of currently known CO_2_ reduction electrocatalysts, nonetheless, materials discovery is needed to enable economically viable, industrial-scale CO_2_ reduction. We report here the largest CO_2_ photocathode search to date, starting with 68860 candidate materials, using a rational first-principles computation-based screening strategy to evaluate synthesizability, corrosion resistance, visible-light absorption, and compatibility of the electronic structure with fuel synthesis. The results confirm the observation of the literature that few materials meet the stringent CO_2_ photocathode requirements, with only 52 materials meeting all requirements. The results are well validated with respect to the literature, with 9 of these materials having been studied for CO_2_ reduction, and the remaining 43 materials are discoveries from our pipeline that merit further investigation.

## Abstract (web summary)

While the conversion of greenhouse CO_2_ to chemical fuels offers a promising renewable energy technology, there is a dire need for new materials. Here, authors report the largest CO_2_ photocathode search using a first-principles approach to identify both known and unreported candidate photocatalysts.

## Introduction

The photocatalytic conversion of CO_2_ to chemical fuels has attracted considerable interest in recent years as it promises a future path to clean, low-cost renewable energy. Among the unresolved challenges impeding economical, industrial-scale, solar-driven reduction of CO_2_, the most daunting is the dearth of photocatalysts which simultaneously provide high product selectivity, high efficiencies as well as long term durability in the highly reducing conditions needed for CO_2_ reduction^1^. While numerous approaches have been used to improve the performance of known photocatalysts, including the use of co-catalysts, sacrificial agents, morphology optimization, and novel architectures such as composite semiconductors, the attempts for finding new photocathode materials have been few and based largely on trial and error^1^.

Through this work, we aim to accelerate materials innovation by performing the largest photocatalyst search to date^2–4^, starting with 68,860 materials, and screening for the electronic state as well as electrochemical stability of the candidate materials, to identify 43 new photocathode materials for CO_2_ reduction. Recent advances in available computing power have facilitated large-scale and predictive first-principles simulations of materials properties through open-source computational databases^5–8^. Such databases have already aided in an exploratory search of new materials for a variety of applications, such as metallic glasses^9^, electrolytes for batteries^10^, and transparent conductors^11^. Leveraging the Materials Project (MP) database^5,6^, we use a rational computational search strategy to identify photocathodes based on the intrinsic properties of materials. This screening strategy allows us to identify semiconductors which not only fulfill metrics for synthesizability, corrosion-resistance—under the highly reducing conditions (< −0.5 V vs RHE) needed for CO_2_ reduction—but also exhibit bandgaps and band-edge energies suited for efficient solar-energy conversion. This computational strategy minimizes the number of computationally expensive electronic structure simulations through a judicious screening of the information which can be queried from existing information in the MP database.

The photocathode materials identified by the tiered computational screening include 9 materials previously reported as CO_2_ photocathodes, as well as discovery of a set of 43 new candidate photocathodes comprised of 34 previously synthesized materials, and 8, as yet, hypothetical structures, providing new chemical classes, inspiration and future prospect for materials design by optimizing photocathode performance through strategies such as thickness variation, nanostructuring, alloying, defect engineering, and use of co-catalysts. We also apply the screening strategy to forty-five photocathodes reported in the literature in order to highlight its scope and demonstrate its viability in identifying suitable, durable photocathodes. Based on the validation against previously reported photocathodes, we find that this screening strategy is aptly designed to identify promising photocathodes, however, some promising photocathodes may have been excluded due to factors such as current limitations in computing power, short-comings of certain computational methods for particular classes of materials, or the specific choice of the screening criteria.

## Results

### Computational strategy for discovery of new photocathodes

**Fig. 1:**
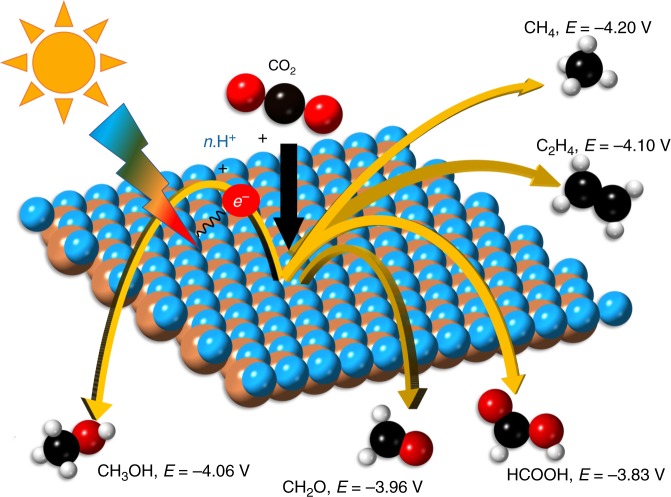
A schematic of photocatalytic reduction of CO_2_ to chemical fuels. Light of sufficient energy can excite electrons across the bandgap of a photocatalyst which can be used to drive the reaction of CO_2_ with hydrogen ions to several closely competing products. At a neutral pH the potential required for converting to each product is noted. The potential for H^+^/H_2_ at this pH is −4.03 eV^63^ with respect to the vacuum level

**Fig. 2:**
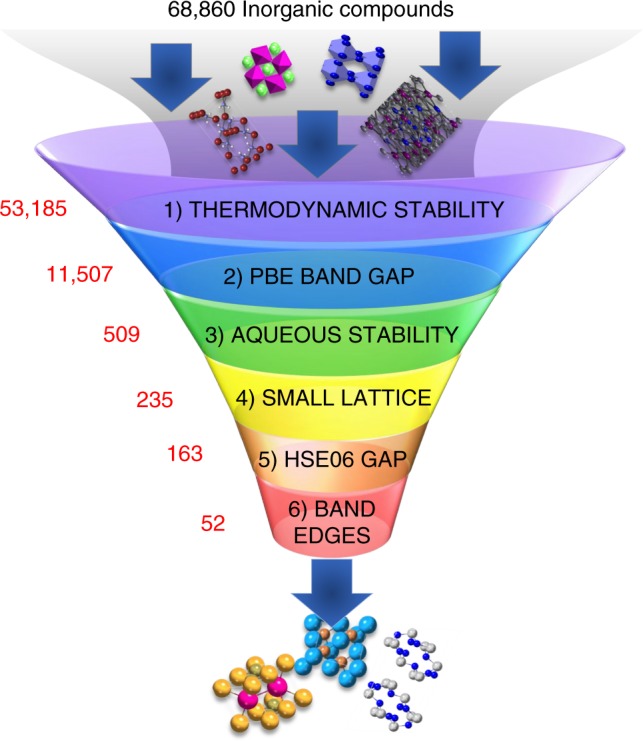
The selection criteria, as well as the number of materials which satisfy the criterion, are shown for each tier. Note that less than 5% of the semiconductors from tier 2 make it through tier 3, highlighting that very few semiconductors are water-stable at the reducing conditions needed for CO_2_ reduction

Suitable photocatalyst surfaces supply photo-excited electrons to facilitate the reaction of CO_2_ with protons in solution. Electrons are excited into the conduction band by the absorption of visible light with a photon energy greater than the bandgap of the photocatalyst material. Electrons of different energies have different thermodynamic propensity for reducing CO_2_ to different fuels, as shown in Fig. 1. The similarity in the reduction potentials for a broad range of fuels poses a challenge for attaining selective photoreduction of CO_2_ and an opportunity for computational screening since a moderate range of target conduction band energies can be used to identify photocathodes for practically any fuel. The resulting photocathode screening pipeline, which is specific to CO_2_ reduction but not to any particular fuel, is shown in Fig. 2 and is composed of six intrinsic property-based screening criteria. The use of progressive tiers, instead of simultaneous evaluation of all target properties, reduces the computational cost associated with the search. The first four tiers are based on computational results already accessible through the MP database, however, the last two tiers are enabled by accurate, more expensive, electronic structure simulations performed specifically for this study. Specifically, the MP database is used to obtain the properties screened in tier 1–4 as well as for accessing the crystal structures which are used for the simulations of tier 5 and 6.

The 68,860 materials available in MP are evaluated using this screening strategy. These materials consist of 34,913 materials reported in the Inorganic Crystal Structure Database (ICSD)^12^ (the world’s largest database of experimentally synthesized and completely characterized inorganic materials) and 33,947 originating from non-ICSD sources, including hypothetical materials predicted by a variety of procedures such as machine learning models of experimental structures^13^, ion substitutions in existing structures, and crowd-sourced user submissions using the MP’s crystal toolkit.

The first tier estimates the thermodynamic stability, through a metric based on the computed energy above the convex hull in the composition space, Δ*E*^hull^, and provides a proxy for the synthesizability of the candidate materials. Δ*E*^hull^ is the energy of decomposition of a material into the set of most stable materials at this chemical composition. We select materials which are predicted thermodynamic ground states, Δ*E*^hull^ = 0 eV.atom^−1^, as well as materials which are meta-stable up to Δ*E*^hull^ < 80 meV.atom^−1^^14^. The cutoff of 80 meV.atom^−1^ is chosen as approximately 80% of known verified sulfides and oxides are within this limit. Of the original 68,860 materials, 53,185 pass the first tier.

The second tier is designed to select materials which have the potential to utilize the visible-light spectrum (1.7 eV–3.0 eV) which accounts for 44% of the solar radiation. The chosen screening range $\left( {1\,{\mathrm{eV}} < E_{{\mathrm{PBE}}}^g < 2.5\,{\mathrm{eV}}} \right)$ takes into account that the calculated electronic structure available through the MP database are computed using PBE and PBE + U functionals, which can underestimate bandgaps with respect to experiment by ~40%^15,16^.

One of the pre-eminent challenges in identifying suitable photocathodes is finding materials which exhibit long-term aqueous stability under reducing conditions. At the reducing potentials necessary for CO_2_ reduction, which typically fall between 0 and −1.0 V vs RHE^1,17,18^, most materials reduce to their metallic forms or hydrolyze. Generally, materials with larger cation and anion sizes and/or small size differences remain stable in aqueous media^19^. Also, multivalent materials tend to have strong lattice energies in comparison to monovalent materials which renders them more likely to be stable in water in comparison to their monovalent counterparts^19^. However, as lattice and hydration enthalpy, and entropy of dissolution and hydration contribute to the Gibbs free energy of dissolution of compounds, generalizations with respect to the stability of specific materials is non-trivial, motivating our evaluation of the electrochemical stability of each candidate photocathode^19^.

In tier 3, we use our recently developed formalism^20,21^ to screen for electrochemically stable materials from the 11,507 materials which pass tier 2. This formalism uses first-principles simulations to generate phase maps in Eh-pH space, or so-called Pourbaix diagrams. Furthermore, it allows us to estimate the electrochemical stability Δ*G*_pbx_, defined as the Gibbs free energy of decomposition of a material to Pourbaix-stable phases at a given pH and potential. We have previously shown that Δ*G*_pbx_, together with an analysis of the predicted decomposition products, provides a quantitative measure for the propensity of materials to be stable in water, either by inherent stability or through the development of a passivating film. Materials with Δ*G*_pbx_ = 0 eV.atom^−1^ are considered to be stable, whereas, for Δ*G*_pbx_ > 0, the candidate materials decompose to Pourbaix-stable phases in thermodynamic equilibrium. We additionally demonstrated that materials with Δ*G*_pbx_ up to 0.5 eV.atom^−1^ can be metastable due to lack of sufficient driving force for decomposition into Pourbaix-stable phases (referred as decomposed species hereon). An inclusive criteria of Δ*G*_pbx_ < 0.2 eV.atom^−1^ for aqueous stability in tier 3 is chosen, also accounting for local temperature and ionic concentration fluctuations of ~10^−2^ M. We screen for materials which decompose to at least one solid phase such that a passivation layer might form on the surface to prevent further corrosion. Only 509 materials, fewer than 5% of those from the previous tier pass the aqueous stability screening. The significant reduction of candidates underscores the importance of determining aqueous stability of potential photocathodes and highlights that electrochemical stability is a much more discriminating requirement than e.g., absorption of visible light.

More generous criteria for aqueous stability for *e*.*g*. Δ*G*_pbx_ < 1.5 eV.atom^−1^ results in 3054 candidate materials. While this expands the list of candidate materials, the materials with large Δ*G*_pbx_ exhibit a high probability of either corrosion or formation of passivating surface films. Passivating films may protect the catalyst surface from corrosion and may even aid in charge migration or separation. However, if the passivation layers exhibit unfavorable electronic properties, for instance if they are electronically insulating they can be detrimental to the photocatalytic reaction. While it is difficult to undertake high-accuracy simulations of tier 5 and 6 for such large number of materials, future advancements in computing power may allow us to relax the aqueous stability criteria to study more materials as well as optimize the catalyst-passivation layer interface for high-efficiency CO_2_ reduction.

We emphasize that the computational screening presented here addresses the challenge of identifying water-stable photocatalysts. In the future, developments in computational methods can facilitate a search for materials which are suited for vapor-phase CO_2_ reduction or CO_2_ reduction in non-aqueous solvents, both of which have shown promising improvements in kinetics and corrosion control^22,23^.

The last two tiers require computationally expensive density-functional theory (DFT) simulations using the HSE06 functional for accurate estimates of the electronic structure, and hence we pre-screen candidates at this stage which are computationally tractable. More specifically, we include materials with 20 or fewer atoms per unit cell, which retains 235 of the 509 materials from tier 3. In tier 5 we filter materials using a hybrid exchange-correlation functional, HSE06, which typically exhibits improved treatment of semiconductor bandgaps, $E_{{\mathrm{HSE06}}}^{\mathrm{g}}$. We retain materials with absorbance in the visible-light range and, allowing for a possible decrease in absorbance due to surface states^24^, up to 0.5 eV beyond the visible-light energy. In total, 163 materials met the criteria of tier 5.

In order to reduce CO_2_, the conduction band minimum (CBM) of a candidate photocatalyst surface should exceed the equilibrium CO_2_ energy, which is approximately −4.2 eV with respect to the vacuum level^1,17^ for most of the reduced products shown in Fig. 1. The single electron reduction of CO_2_ to the anion radical ${\mathrm{CO}}_2^{ \ast - }$ exhibits a higher potential of −2.5 eV^1^. In tier 6, we filter materials surfaces whose CBM are larger than the free energy of reduction or formation of the ${\mathrm{CO}}_2^{ \ast - }$ radical, i.e., −4.3 eV < $E_{{\mathrm{HSE06}}}^{\mathrm{g}}$ < −2.4 eV. Explicit effects of adsorbates, surface reconstructions or solvation is beyond the scope of our study, nonetheless, we note that the large 2 eV window for band-edge criteria abates omission of promising materials. The exploration of reaction mechanisms via calculations of reaction energy barriers, adsorption energy calculations and experimental measurements can shine further light on the efficiency of the individual materials’ surfaces and their selectivity towards specific products.

It is noteworthy that the computational screening performed in this work relies on DFT simulations which has well-known limitations in e.g., the accuracy of the reported bandgap. The use of a consistent DFT simulation parameter sets for computations within a tier and also between the computations of different tiers suggests that any errors are likely systematic, and thus qualitative trends between investigated materials are more likely to be preserved. In addition, our final screening tiers use high-accuracy electronic structure simulations which are specifically helpful in overcoming the limitations associated with bandgap predictions.

**Fig. 3:**
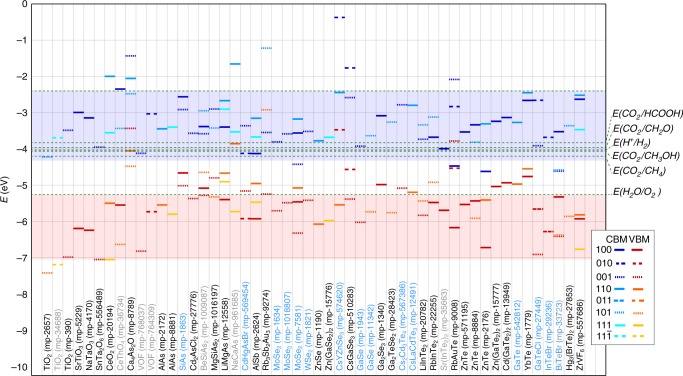
Band-edge energies of materials which pass tier 6 are shown on the *y*-axis and the *x*-axis labels show the corresponding materials formula and the Materials Project material-id in parenthesis. The conduction band minima (CBM) of different planes are marked with lines in shades of blue and the valence band maxima (VBM) in the shades of red as shown in the figure legend. The energy levels *E*(CO_2_/HCOOH), *E*(CO_2_/HCHO), *E*(H^+^/H_2_), *E*(CO_2_/CH_3_OH), *E*(CO_2_/CH_4_), and *E*(H_2_O/O_2_), in decreasing order of energy, are shown as green dashed lines. Note that all potentials are with respect to the vacuum level that is set to 0 eV. The blues shaded region shows the CBM which satisfy the criteria of tier 6. Materials which are reported in the Inorganic Crystal Structure Database (ICSD) are labeled in black, layered-materials are labeled in blue, and those which do not have a corresponding ICSD structure are labeled in gray. BiTeBr is labeled in blue and gray since it is a hypothetical layered material. The phonon spectra of the hypothetical materials are shown in Supplementary Fig. 1

Figure 3 shows the CBM and the valence band maxima (VBM) for the 52 materials, 43 new and 9 previously tested for CO_2_ reduction, which satisfy the criteria for all tiers. The $E_{{\mathrm{HSE06}}}^{\mathrm{g}}$, $E_{{\mathrm{PBE}}}^{\mathrm{g}}$, ICSD-id, materials-id, number of sites in the cell, Δ*E*_hull_, band-edge alignments and Δ*G*_pbx_ for the 235 materials which pass tier 4 are listed in Supplementary Table 1. Surfaces of materials with CBM in the blue shaded region are deemed excellent photocathode candidates for CO_2_ reduction and hydrogen production and those with VBM in the red shaded region may also oxidize water. Before we discuss these newly identified photocathodes and their chemistries, we turn to assess the strengths and short-comings of this screening strategy by applying it to known photocathodes.

### Scope of screening: validation against known photocathodes

The following 45 materials have been reported in the literature as experimentally demonstrating some evidence of photocatalytic CO_2_ reduction:^1,25,26^

1. bulk materials—BiVO_4_, CuInGaSe_2_, CuInS_2_, CuInSe_2_, GaP, InP, and WO_3_
2. nanoparticles—BaTiO_3_, Bi_2_S_3_, Bi_2_WO_6_, CdS, CeO_2_, CuFeO_2_, CuGaO_2_, CuO, Cu_2_O, Cu_2_SnZnS_4_, In_2_Ge_2_O_7_, InNbO_4_, InTaO_4_, KNbO_3_, K_2_Ti_6_O_13_, LiNbO_3_, MnS, MgO, MoSe_2_, NaNbO_3_, NaTaO_3_, Si, SiC, SrTiO_3_, TaNO, Ta_2_O_5_, TiO_2_, W_18_O_49_, WSe_2_, ZnO, ZnS, ZnSe, Zn_2_SnO_4_, ZnTe, and ZrO_2_
3. mesoporous materials—Ga_2_O_3_, Zn(GaO_2_)_2_, and Zn_2_GeO_4_

We note that the crystal structure is not always reported along with the chemical composition, which necessitates the consideration of several polymorphs per reported material. Only CuInGaSe_2_ is not represented by a corresponding phase in the MP database and we note that no crystal structure of CuInGaSe_2_ is reported in the ICSD. The remaining 44 materials comprise a total of 352 phases, whereof 267 phases satisfy the thermodynamic stability criteria of tier 1.

Somewhat surprisingly, tier 2, which removes phases with low probability for visible light absorption, retains 202 phases, corresponding to only 27 materials. Eliminated materials include: CuFeO_2_, CuGaO_2_, CuInS_2_, CuInSe_2_, CuO, Cu_2_O, Cu_2_SnZnS_4_, InP, MnS, ZnO, Zn_2_SO_4_ and W_18_O_49_ with $E_{{\mathrm{PBE}}}^{\mathrm{g}}$ < 1 eV, and InNbO_4_, InTaO_4_, K_2_Ti_6_O_13_, MgO, and ZrO_2_ which exhibit large gaps with $E_{{\mathrm{PBE}}}^{\mathrm{g}}$ > 2.5 eV. These results are overall consistent with experimental measurements. For example, CuO is not a visible-gap material (experimentally measured band gap of 1.2 eV^27^) thus correctly does not pass tier 2. While most materials exhibit PBE bandgaps that are underestimated by ~40% with respect to their experimental gaps, there are pathological exceptions. For example, Cu_2_O is a known strongly correlated-electron material which exhibits a measured bandgap of 2.2 eV^28,29^, however this material is eliminated in tier 2 due to the severe underprediction of its PBE gap, beyond even the generous limits imposed here (1.0 eV < $E_{{\mathrm{PBE}}}^{\mathrm{g}}$ < 2.5 eV). The MP database uses the PBE+U method^30^ to compute the bandgaps of several of the transition metal oxides (see https://materialsproject.org/docs/calculations) to minimize the electron-correlation error from the PBE method. However, such corrections are not employed for the Cu-O chemical family, as a single U value for all Cu oxides has not been found to be universally beneficial^31^.

The most stringent screening is the aqueous stability tier 3, where only 12 materials corresponding to 30 possible phases, satisfy the criteria. These materials are TiO_2_, CeO_2_, Ga_2_O_3_, MoSe_2_, NaTaO_3_, Si, SrTiO_3_, Ta_2_O_5_, Zn(GaO_2_)_2_, ZnTe, ZnSe and WSe_2_. All the other phases exhibit much larger Δ*G*_pbx_, up to 2.6 eV.atom^−1^, and are expected to completely corrode or—in the best case scenario—passivate. The latter scenario depends critically on the ionic conductivity of the surface film and its ability to protect the underlying material from corrosion. For instance, WO_3_ is expected to leach oxygen, to form W metal.

In the subsequent tier 4, where the computational cost is considered, 26 of the 30 phases remain for which HSE06 simulations were performed. After applying the remaining two tiers, the previously tested rutile- and anatase-phases of TiO_2_, cubic-phases of SrTiO_3_, CeO_2_, and NaTaO_3_, the 2*H*-phase of MoSe_2_ and WSe_2_ and the zinc-blende phases of ZnTe and ZnSe are all ascertained as suitable photocathodes. Some new, untested polymorphs of these materials, listed in Supplementary Table 2, were also identified.

It is noteworthy that the aqueous stability screening eliminated the largest proportion of literature-reported photocathodes, demonstrating that our screening procedure is highly selective of materials which are robust under the necessary CO_2_ electroreduction conditions. All materials which emerge from the screening and have been tested for CO_2_ reduction show appreciable photocurrent. It can be concluded from this analysis that the materials predicted as photocathodes in our work have the potential to be excellent photocatalysts and remain robust during operation.

### The identified photocathodes

Among the 52 photocathodes which emerge from the screening, all but eight materials have a corresponding ICSD-id (as shown in Table 1). Among the 44 ICSD materials, barring wurtzite-AlAs, all the materials have been previously synthesized, see Supplementary Table 3. Furthermore, 15 of the screened materials are layered materials as indicated in Fig. 3, a promising class of photocatalyst materials^32^. Among the 52 photocathodes predicted in this work, 43—to our knowledge—have not been tested for CO_2_ reduction as reported in the open literature.

**Table 1:** The spacegroup (SG), the lattice constants (*a*, *b*, *c* in Å, *α*, *β*, *γ* in °), bandgaps computed with HSE06 functional ($E_{{\mathrm{HSE06}}}^{\mathrm{g}}$ in eV), the MP material-id (mp-id) and the ICSD-id for all 52 materials which emerge as suitable photocathodes from the screening are listed in the table

Formula	SG	*a*	*b*	*c*	*α*	*β*	*γ*	$E_{{\mathrm{HSE06}}}^{\mathrm{g}}$	mp-id	ICSD-id	Exp
TiO_2_	*P*4_2_/*mnm*	2.97	4.65	4.65	90.0	90.0	90.0	3.2 (D)	mp-2657	202240	Y [33]
TiO_2_	*C*2/*c*	3.82	3.82	5.43	109.7	109.7	90.1	3.5 (I)	mp-34688	None	N
TiO_2_	*I*4_1_/*amd*	3.81	3.81	5.56	110.0	110.0	90.0	3.5 (I)	mp-390	202242	Y^63^
SrTiO_3_	*Pm*3̄*m*	3.95	3.95	3.95	90.0	90.0	90.0	3.2 (I)	mp-5229	80872	Y^35^
NaTaO_3_	*Pm*3̄*m*	3.98	3.98	3.98	90.0	90.0	90.0	3.1 (I)	mp-4170	88378	Y^64^
Ta_2_SnO_6_	*Cc*	4.92	5.62	9.06	91.4	105.8	90.0	3.1 (I)	mp-556489	54078	N
CeO_2_	*Fm*3̄*m*	3.87	3.87	3.87	60.0	60.0	60.0	3.5 (I)	mp-20194	164225	Y^65^
CeThO_4_	*P*4/*mmm*	3.92	7.84	6.79	30.0	54.7	60.0	3.2 (I)	mp-36734	None	N
Ca_4_As_2_O	*I*4/*mmm*	4.57	4.57	8.39	105.8	105.8	90.0	2.0 (I)	mp-8789	68203	N
VOF	*P*2_1_/*c*	5.19	5.00	5.10	90.0	101.8	90.0	2.7 (D)	mp-768037	None	N
VOF	*Pmmn*	3.87	3.17	6.28	90.0	90.0	90.0	2.7 (I)	mp-764309	None	N
AlAs	*F*3̄*m*	4.05	4.05	4.05	60.0	60.0	60.0	2.1 (I)	mp-2172	606009	N
AlAs	*P*6_3_*mc*	4.05	4.05	6.65	90.0	90.0	120.0	2.4 (I)	mp-8881	67771	N
SiAs	*C*2/*m*	3.70	9.06	10.23	111.8	90.0	101.8	2.1 (I)	mp-1863	43227	N
Cd_2_AsCl_2_	*P*2_1_/*c*	8.00	8.22	9.37	90.0	90.0	116.9	1.8 (I)	mp-27776	26013	N
BeSiAs_2_	*I*4̄2*d*	5.38	5.38	6.57	114.2	114.2	90.0	1.7 (D)	mp-1009087	None	N
MgSiAs_2_	*I*4̄2*d*	5.95	5.95	6.88	115.6	115.6	90.0	1.9 (D)	mp-1016197	182367	N
LiMgAs	*F*4̄3*m*	4.39	4.39	4.39	60.0	60.0	60.0	2.0 (I)	mp-12558	107954	N
NaCaAs	*F*4̄3*m*	4.93	4.93	4.93	60.0	60.0	60.0	2.2 (I)	mp-961685	None	N
CdHgAsBr	*Pmma*	4.80	11.06	10.21	90.0	90.0	90.0	1.8 (I)	mp-569454	240354	N
AlSb	*F*4̄3*m*	4.41	4.41	4.41	60.0	60.0	60.0	1.8 (I)	mp-2624	609288	N
Rb_3_Sb_2_Au_3_	*R*3̄*m*	8.29	8.29	8.29	47.4	47.4	47.4	1.7 (D)	mp-9274	78978	N
MoSe_2_	*P*6_3_/*mmc*	3.33	3.33	15.45	90.0	90.0	120.0	1.9 (D)	mp-1634	49800	Y^25^
MoSe_2_	*P*6_3_/*mmc*	3.33	3.33	14.27	90.0	90.0	120.0	1.9 (I)	mp-1018807	644346	N
MoSe_2_	*R*3*m*	7.61	7.61	7.61	25.3	25.3	25.3	1.9 (I)	mp-7581	16948	N
WSe_2_	*P*6_3_/*mmc*	3.33	3.33	15.07	90.0	90.0	120.0	1.9 (I)	mp-1821	40752	Y^25^
ZnSe	*F*4̄3*m*	4.06	4.06	4.06	60.0	60.0	60.0	2.3 (D)	mp-1190	652224	Y^26^
Zn(GaSe_2_)_2_	*I*4̄	5.64	5.64	6.81	114.5	114.5	90.0	2.3 (D)	mp-15776	168594	N
CsYZnSe_3_	*Cmcm*	4.18	8.37	11.03	90.0	90.0	104.5	3.1 (D)	mp-574620	280847	N
CsGaSe_3_	*P*2_1_/*c*	6.91	7.97	13.30	90.0	90.0	106.5	2.8 (I)	mp-510283	98670	N
GaSe	*P*6_3_/*mmc*	3.82	3.82	17.75	90.0	90.0	120.0	2.1 (I)	mp-1943	63122	N
GaSe	*R*3*m*	9.13	9.13	9.13	24.1	24.1	24.1	2.1 (I)	mp-11342	73388	N
Ga_2_Se_3_	*Cc*	6.81	6.77	6.90	81.0	60.4	71.4	1.9 (D)	mp-1340	37168	N
Ga_2_TeSe_2_	*I*4_1_*md*	7.44	7.44	7.61	119.3	119.3	90.0	2.5 (D)	mp-28423	64617	N
Cs_2_Cd_3_Te_4_	*Ibam*	6.89	10.92	10.92	75.9	71.6	71.6	2.3 (D)	mp-567386	90369	N
CsLaCdTe_3_	*Cmcm*	4.72	8.98	12.31	90.0	90.0	105.2	2.4 (D)	mp-12491	173316	N
LiInTe_2_	*I*4̄r2*d*	6.54	6.54	7.88	114.5	114.5	90.0	2.1 (D)	mp-20782	639906	N
RbInTe_2_	*I*4/*mcm*	7.42	7.42	7.42	104.9	104.9	119.1	1.8 (I)	mp-22255	75346	N
Sr(InTe_2_)_2_	*I*4/*m*	7.21	7.21	7.21	104.5	104.5	119.8	1.7 (I)	mp-35663	None	N
RbAuTe	*Pmma*	5.25	6.01	7.40	90.0	90.0	90.0	1.7 (D)	mp-9008	71652	N
ZnTe	*P*3_1_	4.37	4.37	10.70	90.0	90.0	120.0	2.0 (D)	mp-571195	80076	N
ZnTe	*P*6_3_*mc*	4.37	4.37	7.18	90.0	90.0	120.0	2.1 (D)	mp-8884	67779	N
ZnTe	*F*4̄3*m*	4.37	4.37	4.37	60.0	60.0	60.0	2.1 (D)	mp-2176	41984	Y^41^
Zn(GaTe_2_)_2_	*I*4̄	6.07	6.07	7.39	114.3	114.3	90.0	1.8 (I)	mp-15777	44888	N
Cd(GaTe_2_)_2_	*I*4̄	6.27	6.27	7.47	114.8	114.8	90.0	1.8 (D)	mp-13949	25646	N
GaTe	*C*2/*m*	4.15	9.35	10.82	106.1	90.0	102.8	1.7 (D)	mp-542812	153456	N
YbTe	*Fm*3̄*m*	4.50	4.50	4.50	60.0	60.0	60.0	2.1 (I)	mp-1779	653185	N
GaTeCl	*Pnnm*	4.16	5.98	15.91	90.0	90.0	90.0	3.0 (D)	mp-27449	15582	N
InTeBr	*P*2_1_/*c*	7.84	7.74	8.54	90.0	117.2	90.0	2.6 (D)	mp-29236	100705	N
BiTeBr	*P*3*m*1	4.36	4.36	6.91	90.0	90.0	120.0	1.8 (I)	mp-33723	None	N
Hg_3_(TeBr)_2_	*I*2_1_3	8.59	8.59	8.59	109.5	109.5	109.5	2.5 (D)	mp-27853	27402	N
ZrVF_6_	*Fm*3̄*m*	5.87	5.87	5.87	60.0	60.0	60.0	3.3 (I)	mp-557686	73354	N

Materials with reported activity for CO_2_ reduction are denoted in the Exp column as Y along with the corresponding reference and N otherwise. Direct and indirect bandgaps are denoted (D) and (I), respectively, in the $E_{{\mathrm{HSE06}}}^{\mathrm{g}}$ column

Among the 43 newly identified photocathodes listed in Table 1, 35 have been previously synthesized (see Supplementary Table 3 for the references of synthesis procedures) and 8 are as-yet hypothetical materials. The phonon spectra of the 8 hypothetical materials, Supplementary Fig. 1 and Supplementary Note 1, show that BeSiAs_2_, BiTeBr, CeThO_4_, and NaCaAs are dynamically stable. However, the monoclinic TiO_2_, SrIn_2_Te_4_ and the two phases of VOF are dynamically unstable and are removed from the list of high-ranking candidates. The crystal structure of all the 52 identified photocathodes are shown in Supplementary Figs 3–54.

We also note that most prior experimental data on photocatalytic CO_2_ reduction focuses on oxides. Our work identifies promising photocathodes from a wider range of chemistries which may have been overlooked, including 11 oxides, 9 arsenides, 2 antimonides, 12 selenides, 17 tellurides, and one transition-metal fluoride. Among the 9 previously known photocathodes, 5 are oxides and 3 are selenides. Only one of the 17 predicted tellurides has been tested, i.e., ZnTe. To the best of our knowledge, none of the arsenide or antimonide photocathodes identified in this work have been tested before.

The rutile- and anatase-phases of TiO_2_ are undoubtedly the most studied photocathodes thus far^33,34^. By using different morphologies and various co-catalysts, a range of reduced products including ethane, methanol, formic acid, and CO have been obtained from TiO_2_ catalysts^33,34^. Other previously studied oxides include SrTiO_3_, NaTaO_3_, and CeO_2_, each of which can form nanoparticles^35^ with reported CO_2_ reduction activity.

Among the oxide photocathodes identified in this work, CeThO_4_ is an as yet hypothetical structure, whereas SnTa_2_O_6_ and Ca_4_As_2_O have been synthesized^36,37^. Heating of KTaO_3_ and SnCl_2_ at a high-temperature of 673 K for 24 h produces SnTa_2_O_6_^36^ and the reaction of Ca_3_P_2_ and Ca_3_As_2_ yields Ca_4_As_2_O^37^. SnTa_2_O_6_ was recognized as a promising photocathode by another study^38^ but it is yet to be experimentally tested. Extraordinarily, none of the identified arsenides have been tested for CO_2_ reduction, even the commercially well-used, high-electron mobility, zinc blende-AlAs. Barring wurtzite-AlAs, BeSiAs_2_, and NaCaAs, all candidate arsenides have been previously synthesized. Typically, they are produced from prolonged high-temperature reactions of constituent elements or compound-phases mixed in a stoichiometric ratio.

Cationic substitutions in the MgSiAs_2_ and the LiCaAs phase yields the hypothetical materials BeSiAs_2_ and NaCaAs, presenting an operative technique to modulate the band-edge energies as shown in Fig. 3. Likewise, the bandgap, band-edge positions and the band-transitions of layered materials, such as the SiAs and CdHgAsBr, can be altered by varying the number of layers, intercalation or application of strain, thus, providing added avenues to tune product selectivity^32^.

Two antimonides are identified as promising photocathodes, the high-temperature cubic-phase of AlSb and the trigonal Rb_3_Sb_2_Au_3_. Both the materials have been synthesized before but neither has been tested for CO_2_ reduction.

Compared to oxides, selenides have lower bandgaps in the visible-region and can utilize a large fraction of the solar spectrum. Among the twelve identified selenide photocathodes only three have been tested before, the transition-metal dichalcogenides, 2*H*-MoSe_2_ (mp-1634) and 2*H*-WSe_2_ (mp-1821)^25^ and the cubic-ZnSe (mp-1190) nanosheets with a Ni co-catalyst^26^. All three reduce CO_2_ to CO.

All other identified selenide photocathodes, a 2-layered MoSe_2_ polymorph, a 4-layered MoSe_2_ polymorph, Zn(GaSe_2_)_2_, CsYZnSe_3_, CsGaSe_3_, hexagonal-GaSe, trigonal-GaSe, Ga_2_Se_3_, and Ga_2_TeSe_3_ have been experimentally synthesized but are yet to be tested for CO_2_ reduction. High-quality crystals of these materials can be grown via high-temperature synthesis methods such as the Bridgman technique and chemical vapor transport (CVT).

The hexagonal-GaSe (mp-1943) is particularly promising since both *n*- and *p*-type crystals can be synthesized for this phase^39^. The defected zinc-blende structures of Ga_2_TeSe_2_ and Ga_2_Se_3_ also promise defect-mediated faster mobility of photogenerated electrons and holes. Furthermore, these two materials have direct gaps which can allow direct photon-induced electron transfer without phonon-assistance. In total, half of the predicted twelve selenides are layered materials.

A large number of telluride photocathodes have been identified through this work, largely because these materials exhibit excellent aqueous stability under reducing conditions, which is intuitive given the significantly larger aqueous stability region of elemental Te, as compared to Se and S^40^. Among the 17 telluride photocathodes only the zinc-blende phase of ZnTe (mp-2176), grown on Zn/ZnO nanowires, has been tested before and reduces CO_2_ to CO^41^.

Two new phases of ZnTe, a trigonal-^42^, and a hexagonal^43^-phase have been identified as photocathodes. As a high-pressure phase, the trigonal structure is not as suitable as the hexagonal phase for CO_2_ reduction. In addition to the ZnTe phases, two other binary telluride photocathodes are identified through this work, YbTe and the layered GaTe.

All candidate tellurides except BiTeBr have been synthesized before. Akin to the selenides, the tellurides have been synthesized using the Bridgman technique, CVT and also the reactive halide synthesis method. Most of the ternary tellurides have reported facile synthesis procedures and are stable in air. However, previous work shows that obtaining phase pure Zn(GaTe_2_)_2_^44^, is challenging, LiInTe_2_ oxidizes in air^45^, and GaTeCl and InTeBr are hygroscopic^46^. Notably, the layered ternary Cs_2_Cd_3_Te_4_^47^ and CsLaCdTe_3_^48^ exhibit bandgaps 2.3 eV and 2.4 eV, which are much larger than that of the 1.5 V of the well-known CdTe^49^.

The compounds that have been successfully synthesized with *p*-type conductivity are perhaps the most amenable to experimental investigation as CO_2_ photocathodes, and those with no reported conductivity or doping studies may require development of doping strategies before photoelectrochemical performance can be assessed. The presence of steps, edges, defects, grain-boundaries, and surface adsorbates have been known to have significant effects on catalyst rates and selectivities for CO_2_ reduction specifically^50^, which is not addressed in this study but is likely to further differentiate the screened materials. While the experimental path connecting this computational screening to an operational device may be quite tortuous, our screening pipeline is designed to identify the materials and chemistry classes that, upon successful traversal of that path, have the requisite operational stability to create a deployable technology.

## Discussion

To summarize, we have performed the largest exploratory search, covering 68,860 materials, for CO_2_ reduction photocathodes with targeted intrinsic properties, including corrosion resistance, and identified 39 new materials which have never been reported for this functionality before. We design and employ a computational screening strategy which makes the monolithic task of computing accurate electronic structure properties manageable by pre-screening materials based on computed properties available through first-principles simulations-based databases. After applying the screening strategy to photocathodes reported in the literature, we find that the strategy is highly selective of materials which are extremely robust in the reducing conditions needed for CO_2_ reduction. The predicted materials include diverse chemistries, such as arsenides, tellurides, selenides, and oxides and include several layered materials. This diversity presents ample opportunity for further materials design via alloying, application of strain, use of co-catalyst or use of tandem designs. Even though special emphasis is placed on materials with surfaces which can reduce CO_2_, several materials obtained from the screening strategy are also suitable for solar-driven water oxidation and hydrogen production or as solar energy absorbers. We hope that this work will open up new avenues and inspiration for materials optimization towards viable solar fuel production.

## Methods

All the simulations are based on density functional theory using the projector-augmented wave method as implemented in the plane-wave code VASP^51–54^. The ground state structures, energies as well as the bandgap computation, in tier 1 and 2, use the PAW method for modeling core electrons and the Generalized Gradient Approximation with the PBE parameterization is used for the exchange and correlation^55–58^. An energy cutoff of 520 eV and a *k*-point mesh of 1000/number of atoms in the cell is used for all simulations. The energy difference for ionic convergence is set to 0.0005 eV × number of atoms in the cell. These parameters yield well-converged structures in most instances^59^. Some elements have been modeled using PBE+U and an energy correction is used to make them comparable with the PBE calculations^60^. Oxides containing Co, Cr, Fe, Mn, Mo, Ni, V, or W are modeled using PBE+U with U values of 3.32 eV, 3.7 eV, 5.3 eV, 3.9 eV, 4.38 eV, 6.2 eV, 3.25 eV, and 6.2 eV, respectively.

An ionic concentration of 10^−5^ M, activity of solids as 1, temperature of 298 K, and a pressure of 1 atm is assumed for the Δ*G*_pbx_ computations of tier 3.

To account for the well-known bandgap underestimation problem in PBE and PBE+U methods we adopt the HSE06 functional for tier 5 and 6. HSE06 is a hybrid functional featuring local fractional exact exchange, 25 % for this work, which is predictive for bandgaps for a range of materials^61^. HSE06 simulations were performed with starting structures as the MP optimized bulk structures, and systems failing to converge are discussed in the Supplementary Note 2. The relation between the $E_{{\mathrm{HSE06}}}^{\mathrm{g}}$ and $E_{{\mathrm{PBE}}}^{\mathrm{g}}$ gaps of the materials with pass tier 4 are shown in Supplementary Fig. 2 and discussed in Supplementary Note 2.

Band-edge simulations for tier six were performed in the slab geometry with minimum slab-thickness of 15 Å and a minimum vacuum spacing of 12 Å. Three slabs, the ones with lowest number of atoms for (*hkl*) where *h*, *k*, *l* = 0, 1, −1, were considered for each of the 163 materials which emerge from tier 5 resulting in a total of 489 surfaces. Slabs were generated by the pymatgen package^62^. A dipole correction as implemented in VASP was added in the direction perpendicular to the vacuum spacing of the slabs. The slab geometry for surfaces of materials which pass tier 5 consisted of 6–160 atoms for the various surfaces. Due to the large number of atoms as well as the polarity of certain surface terminations, the HSE06 band-edge simulations were converged for 126 surfaces corresponding to 76 materials. Surfaces with more than 72 atoms did not converge in the 96 hrs run time limit. See supplementary methods for the simulation method and parameters used to compute the phonon dispersion of the eight hypothetical materials.

An energy cutoff of 520 eV is employed for all calculations except the more expensive band-edge slab-simulations where the energy cutoff was reduced to 400 eV. A *k*-point mesh of 1000/(number of atoms in the cell) was used for all but slab calculations where a *k*-point mesh of 30/*a* × 30/*b* × 1 is used (*a* and *b* are the cell dimensions in direction perpendicular to the vacuum spacing). All computations are performed with spin polarization and with magnetic ions in a high-spin ferromagnetic initialization.

## Supplementary information

Supplementary Information

## Data Availability

The data that support the results within this paper and other findings of this study are available at https://materialsproject.org/#search/materials, the supplementary information and from the corresponding author upon reasonable request.
