# Supplementary material for: Robust and synthesizable photocatalysts for CO2 reduction: a data-driven materials discovery
Source: Nat Commun. 2019 Jan 25;10:443. doi: 10.1038/s41467-019-08356-1 (PMC6347635; doi:10.1038/s41467-019-08356-1)
Supplement: Supplementary file 1 — Supplementary Information [file 41467_2019_8356_MOESM1_ESM.pdf]

Supplementary Information for

Robust and Synthesizable Photocatalysts for CO_2 Reduction: A Data-Driven Materials Discovery

Singh et al.

Supplementary Figures

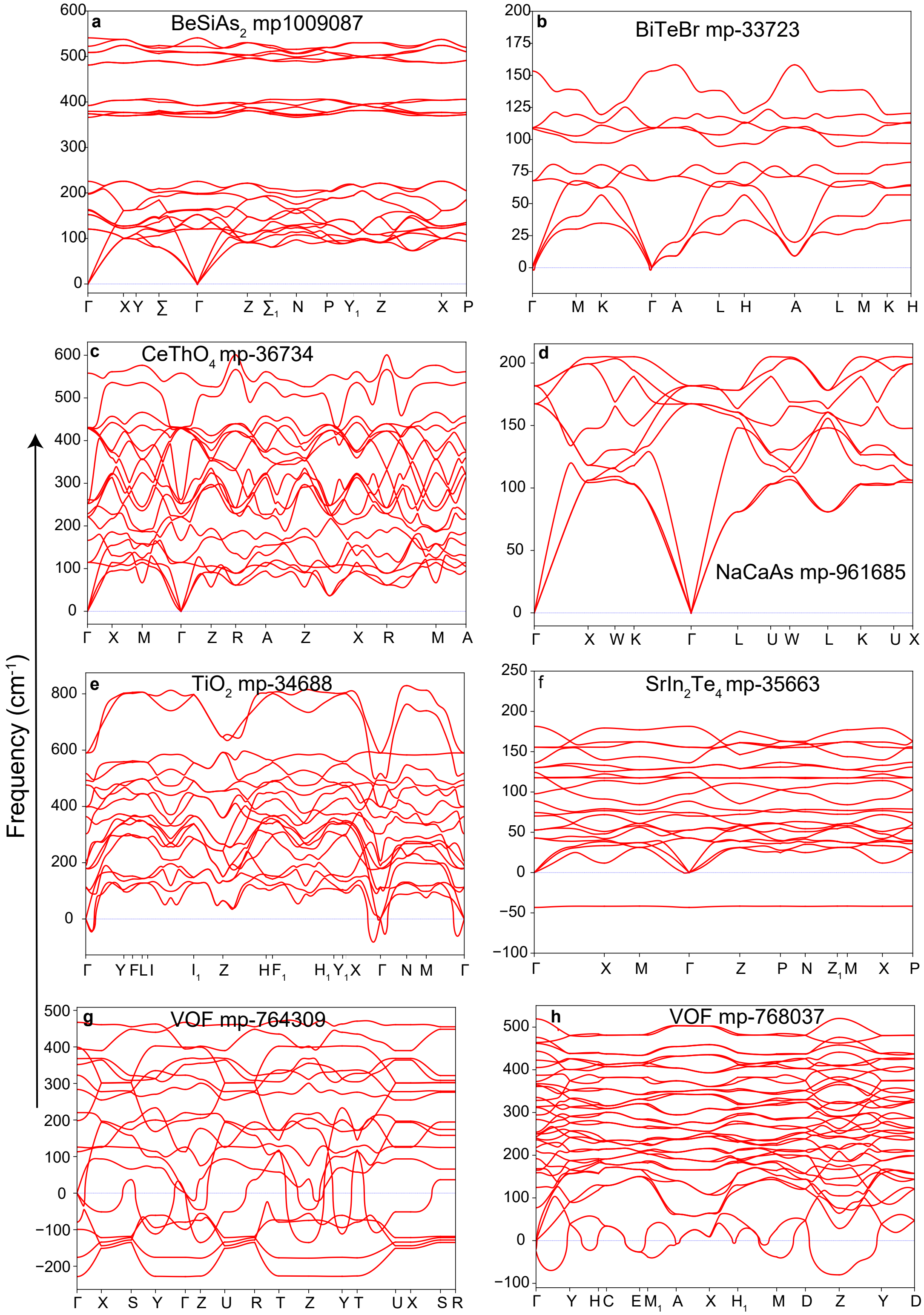

Supplementary Figure 1: Phonon spectra of the hypothetical materials identified in this work. Sub-figure inset shows the formula of the materials and their Materials Project materials ids. Note that a 3x3x3 supercell was used to compute the spectra in (a)-(e) and a 1x1x1 supercell was used for the spectra in (f)-(h).

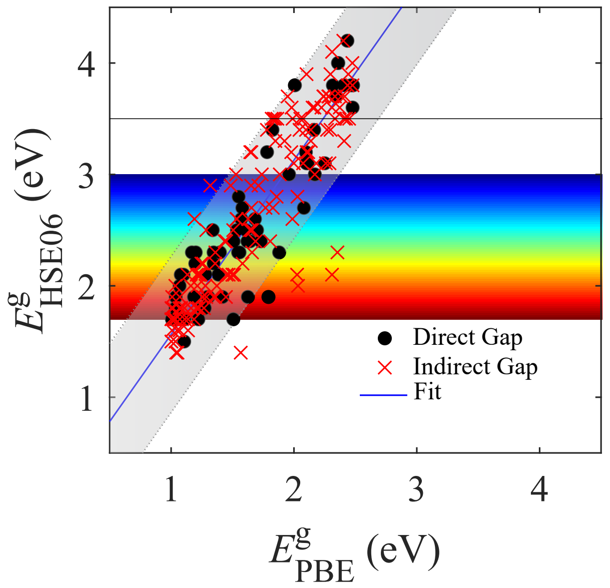

Supplementary Figure 2: A comparison of the bandgaps computed from PBE or PBE+U functional, E^{g}_{PBE}, and the HSE06 functional, E^{g}_{HSE06}, for the materials which pass tier four. Materials with E^{g}_{HSE06} between 1.7 eV to 3.5 eV are marked with the black bounds. Visible light range is marked by corresponding colors. The 95 % prediction bounds for $E^{g}_{HSE06} = 1.6\ E^{g}_{PBE}$ fit are marked as shaded gray regions. The direct gap materials, total 62 materials, and indirect gap materials, 142 materials, are distinguished with black circles and red cross symbols, respectively. See Supplementary Table 1 for bandgap values of specific materials.

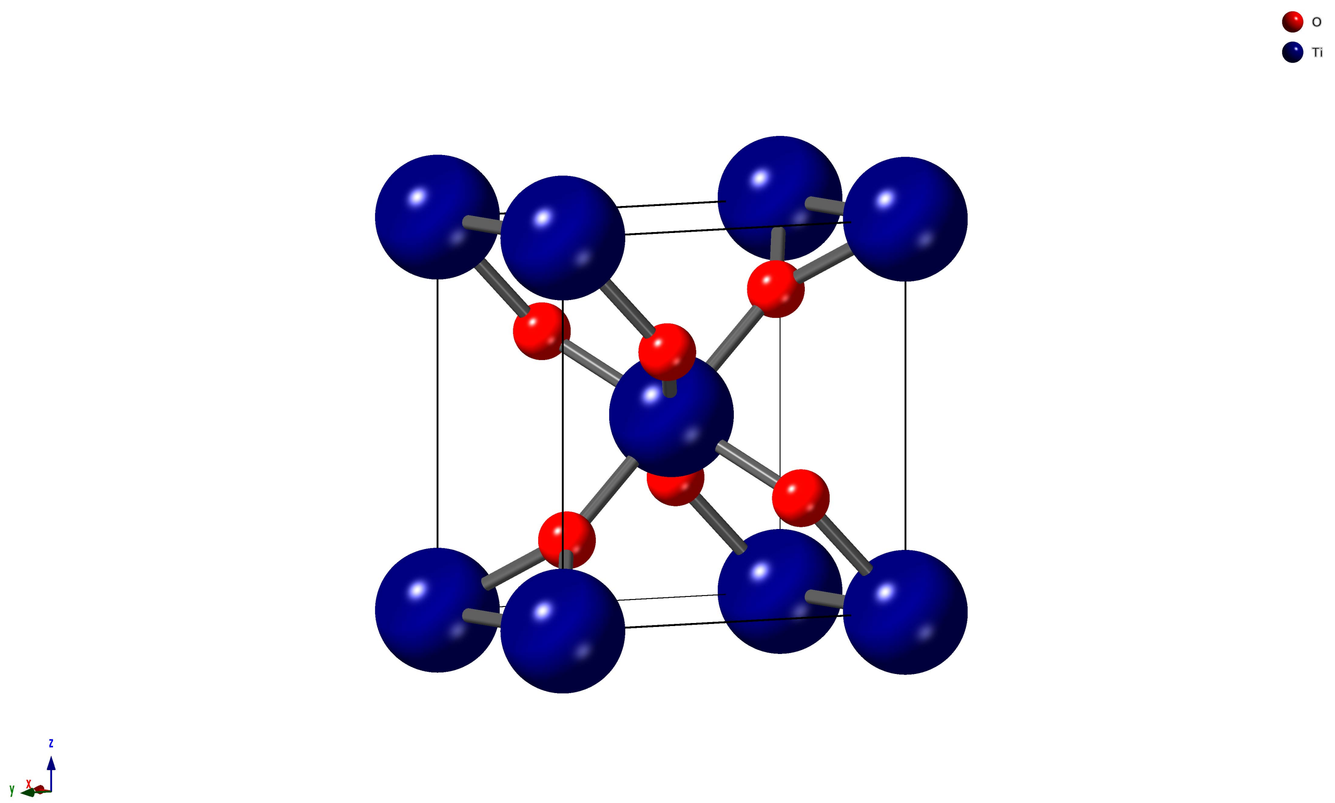

Supplementary Figure 3: Crystal structure of TiO_2 (mp-2657)

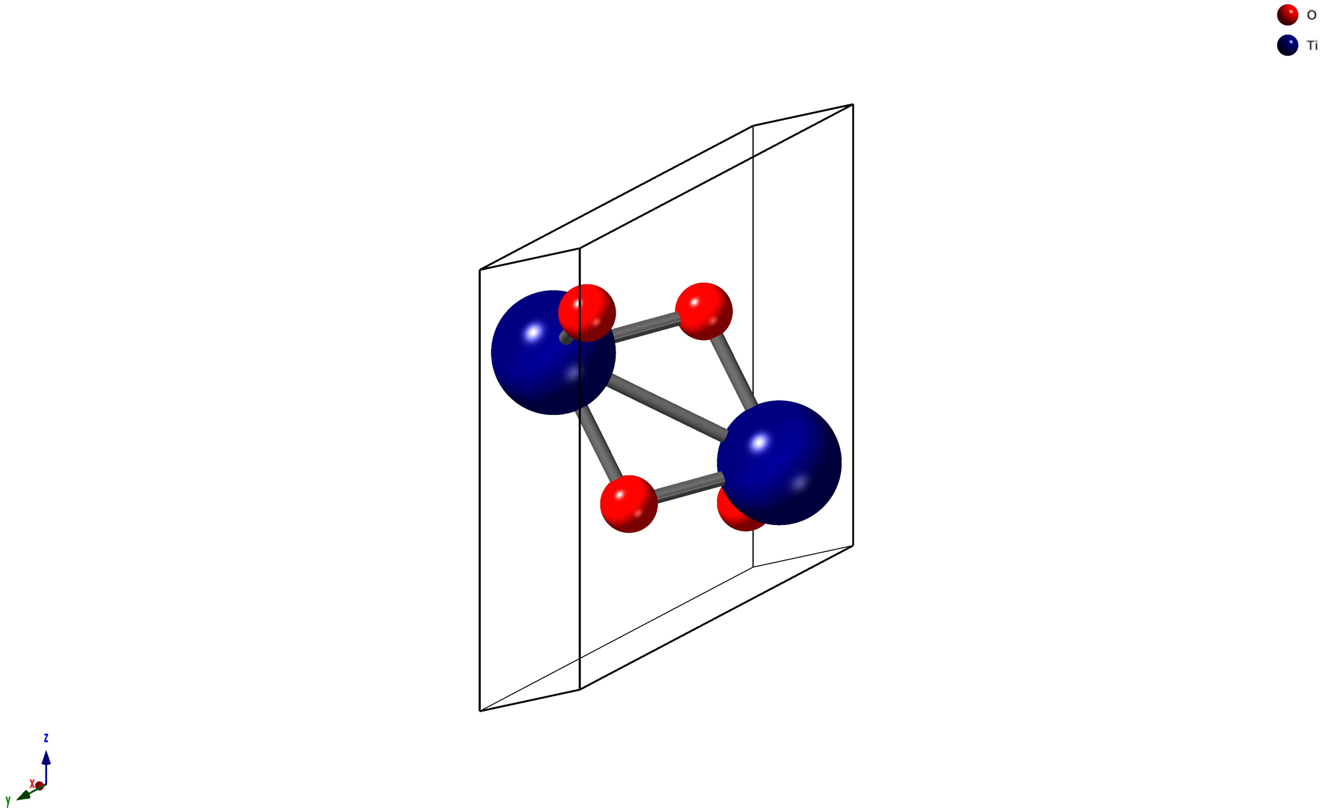

Supplementary Figure 4: Crystal structure of TiO_2 (mp-34688)

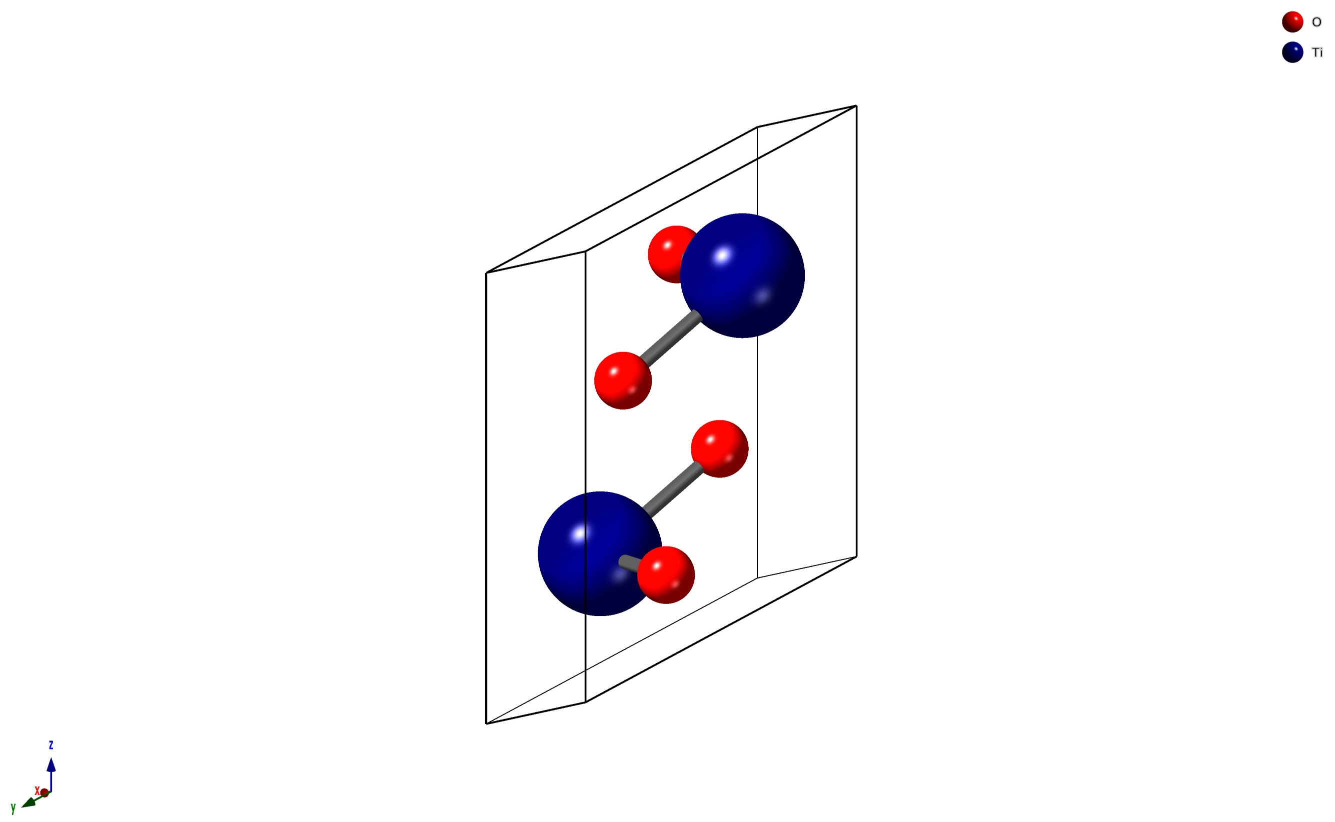

Supplementary Figure 5: Crystal structure of TiO_2 (mp-390)

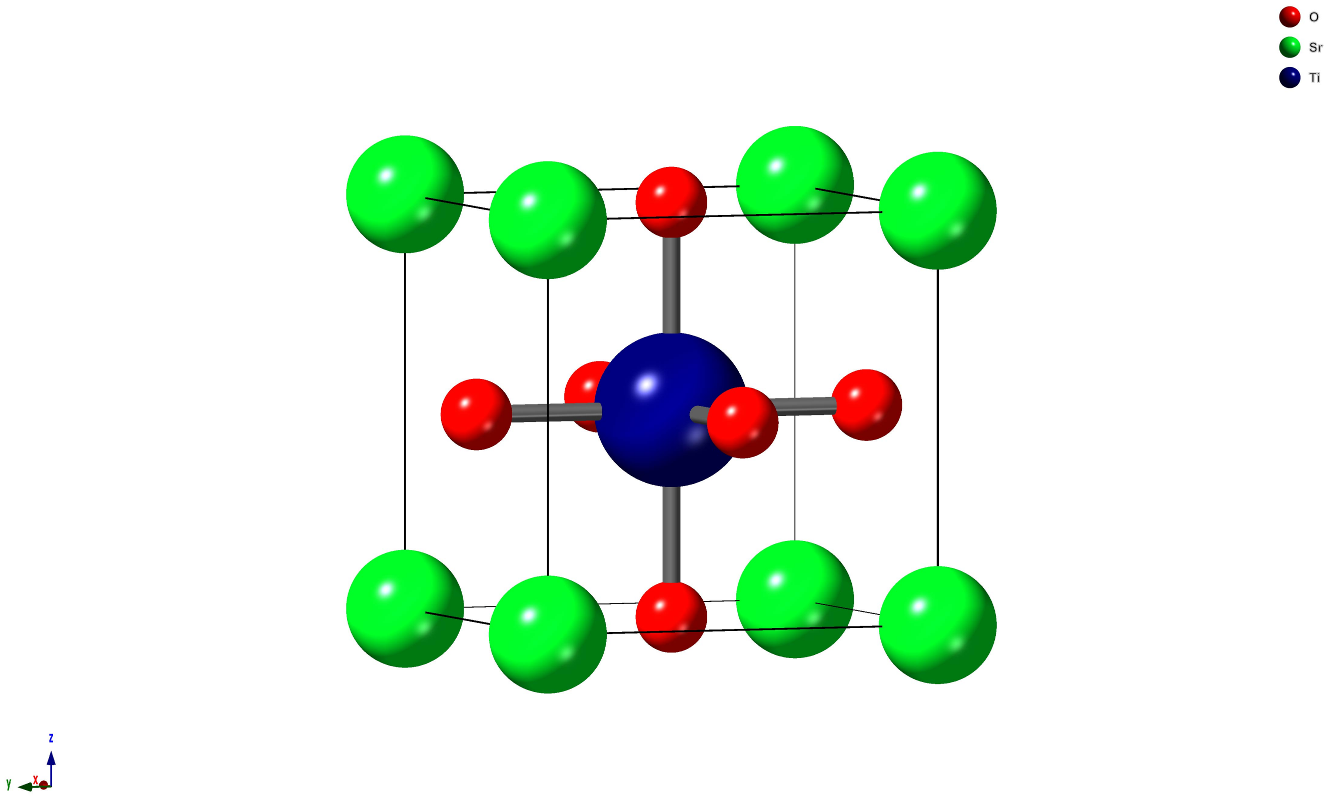

Supplementary Figure 6: Crystal structure of $SrTiO_3$ (mp-5229)

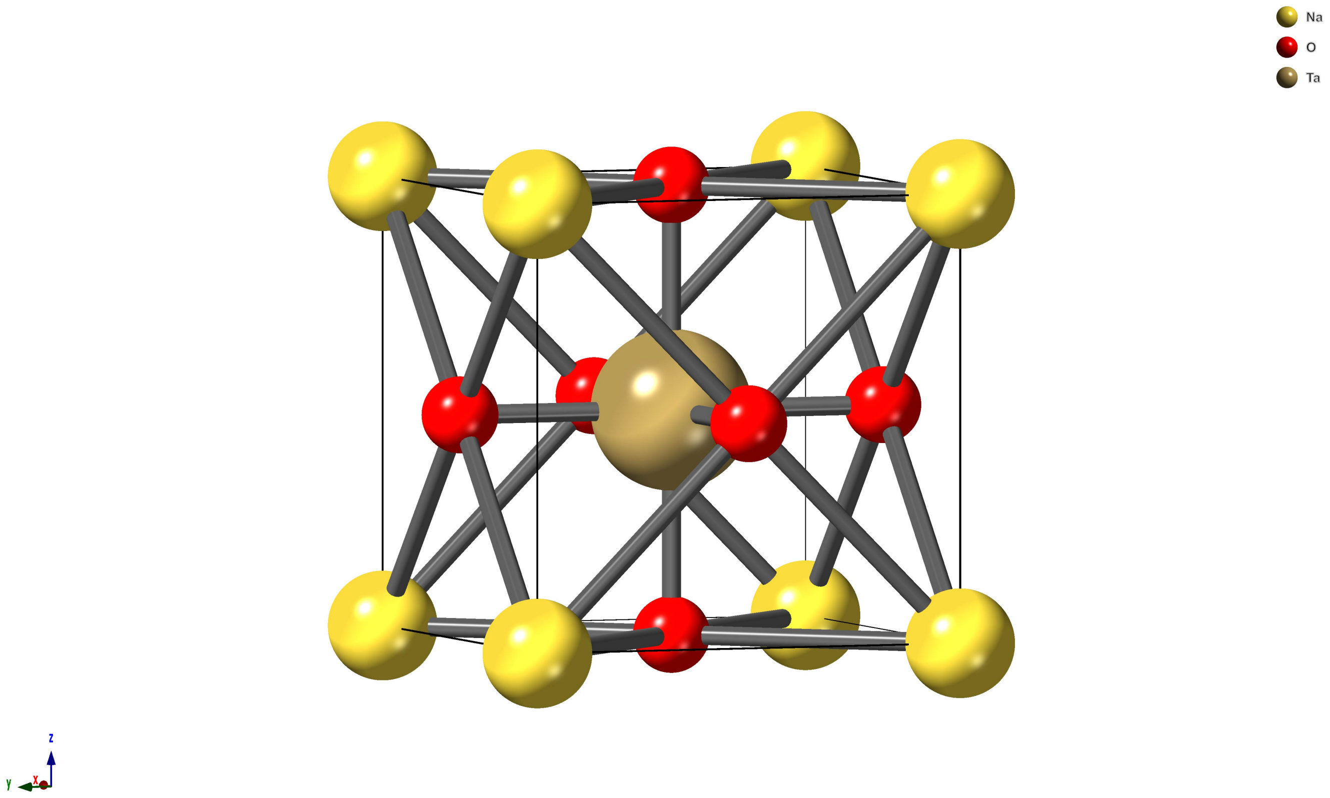

Supplementary Figure 7: Crystal structure of $NaTaO_3$ (mp-4170)

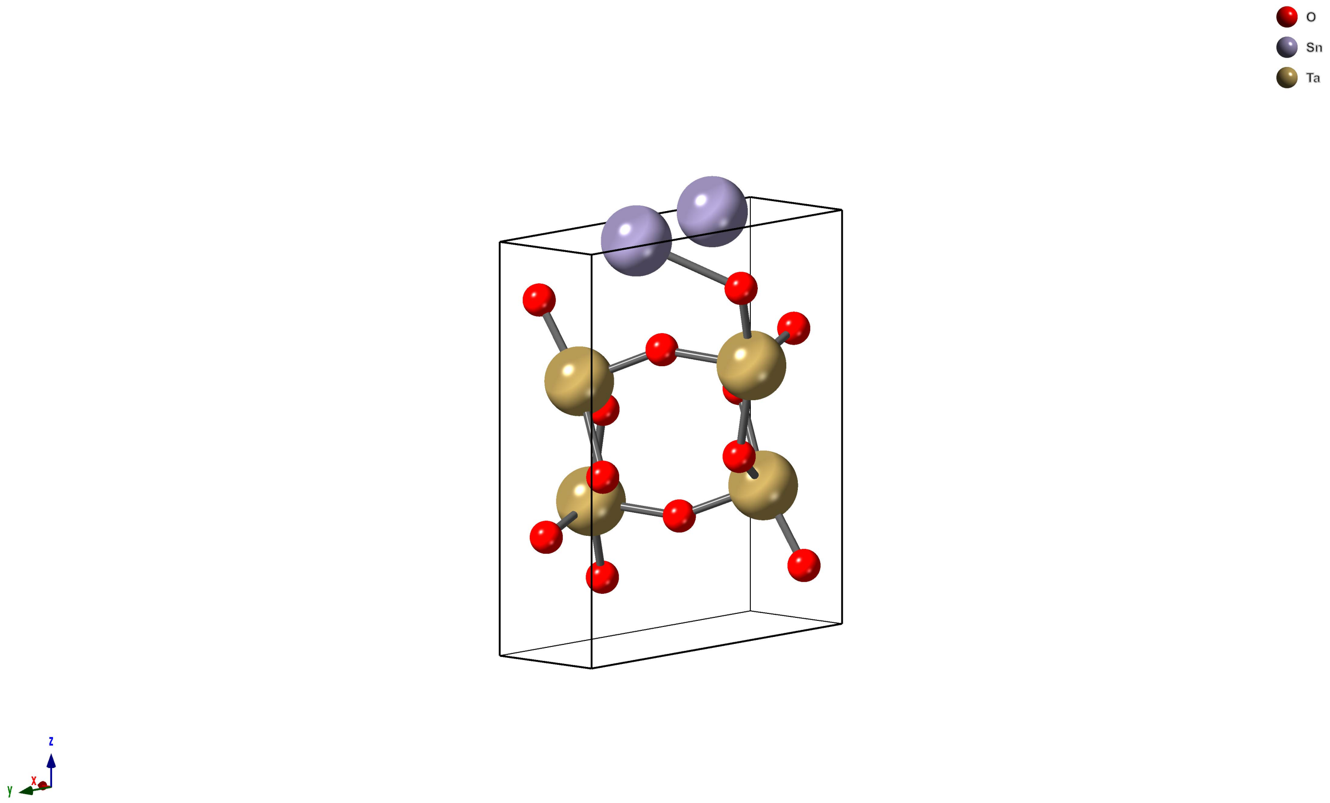

Supplementary Figure 8: Crystal structure of Ta_2SnO_6 (mp-556489)

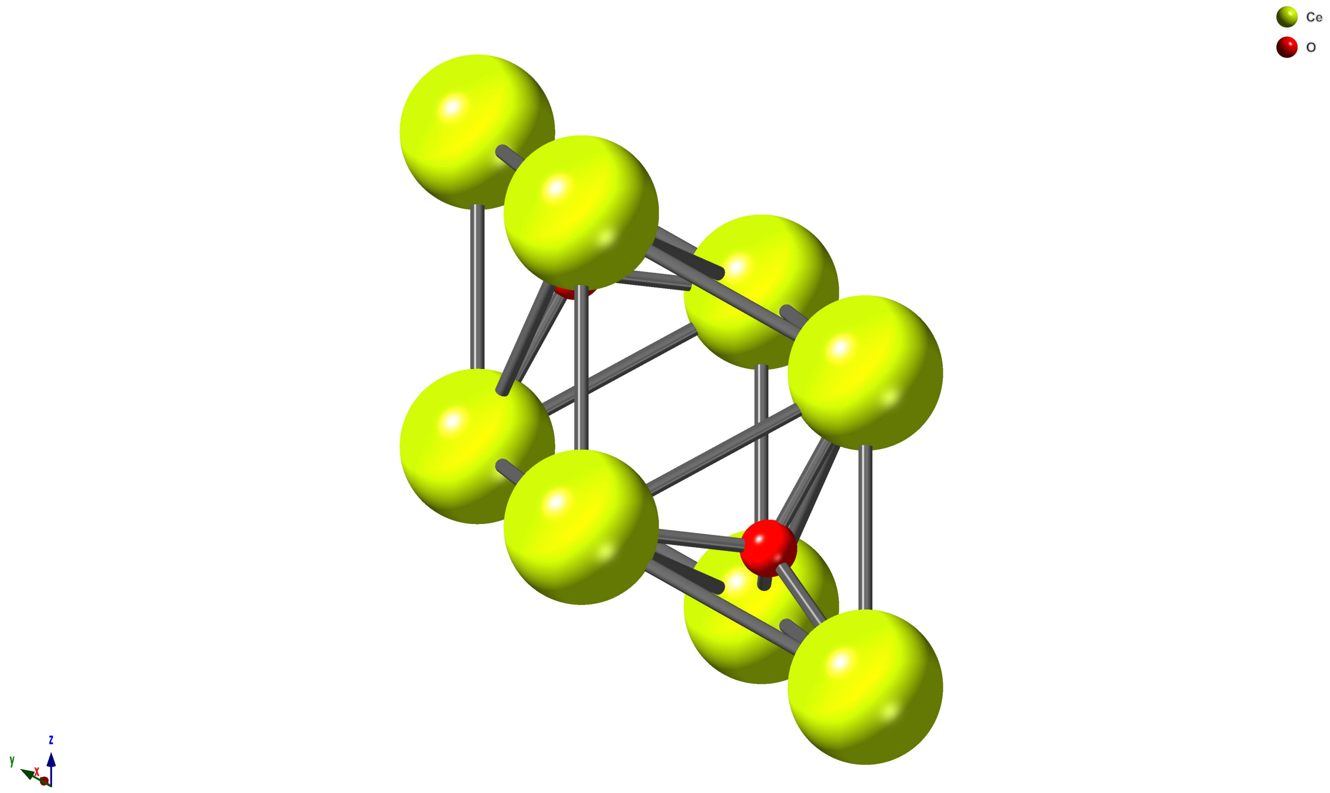

Supplementary Figure 9: Crystal structure of CeO_2 (mp-20194)

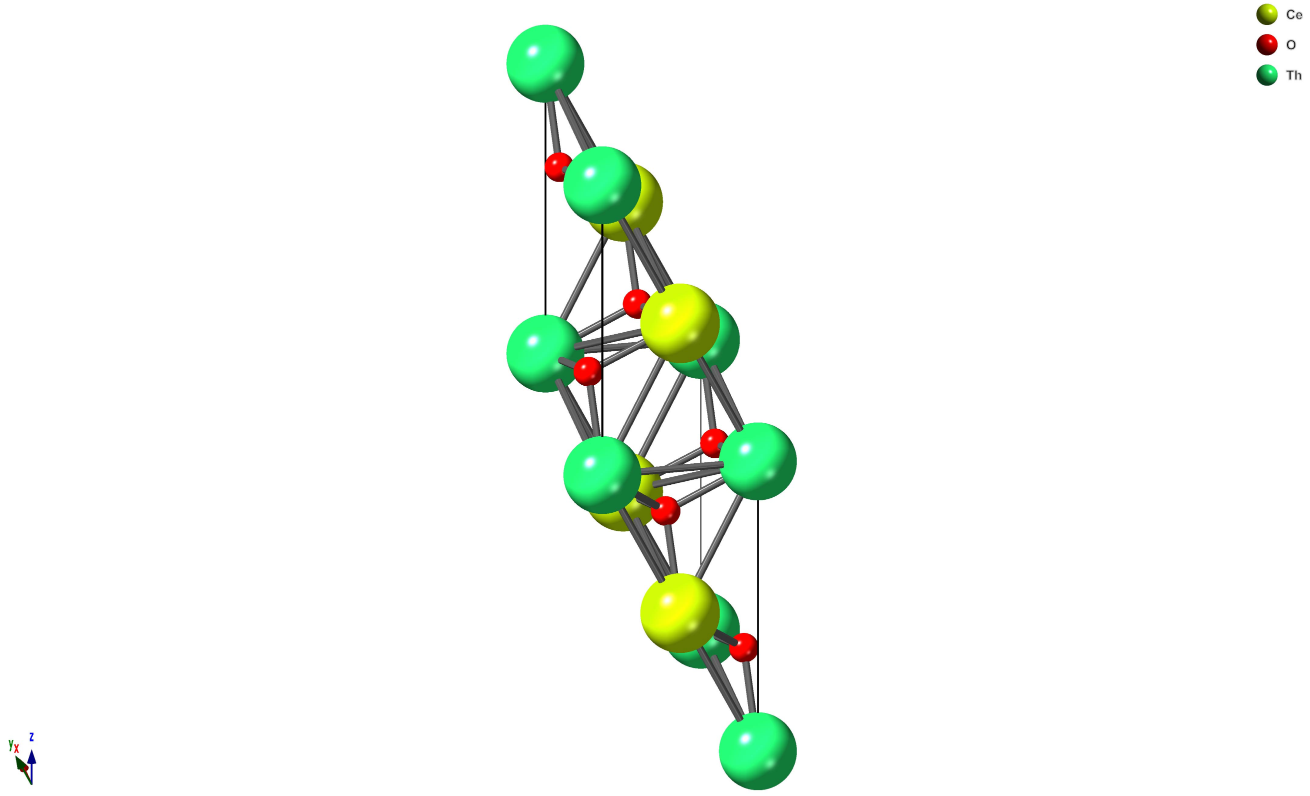

Supplementary Figure 10: Crystal structure of $CeThO_4$ (mp-36734)

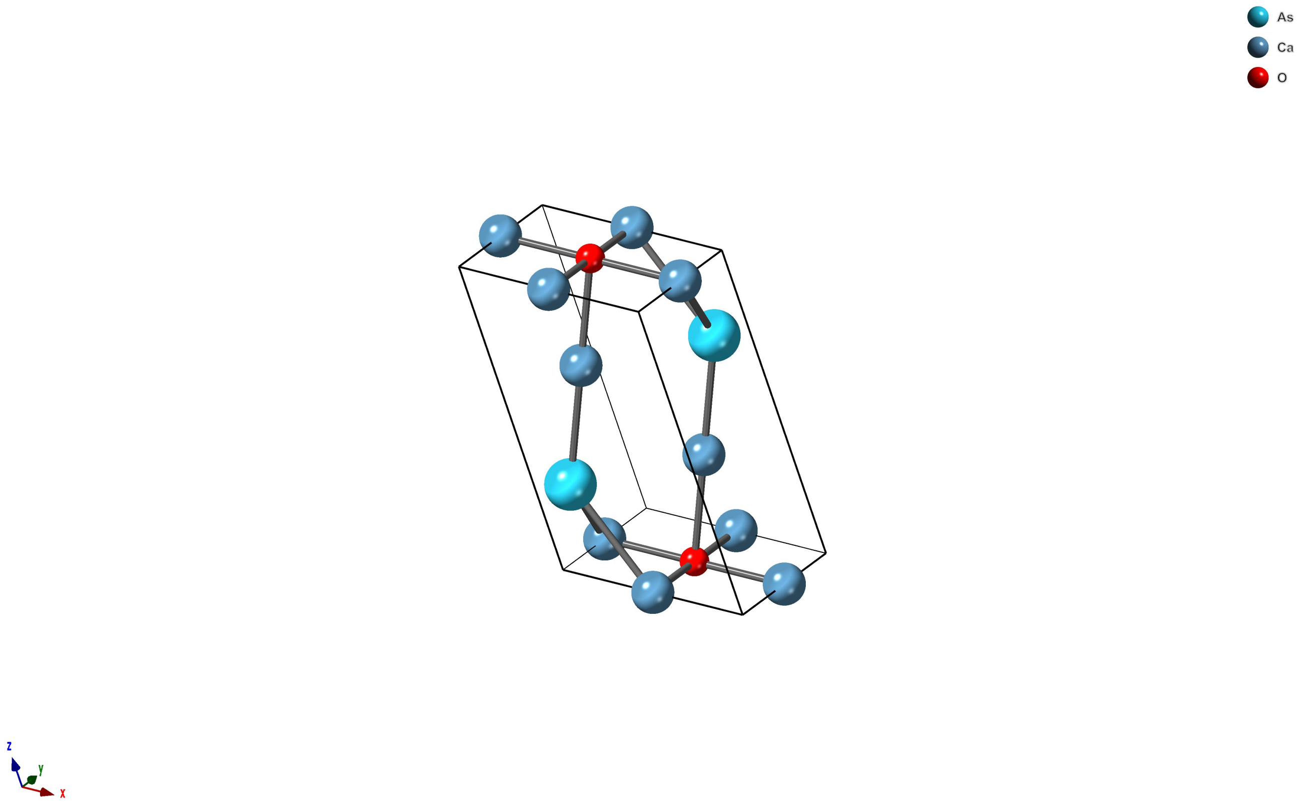

Supplementary Figure 11: Crystal structure of Ca_4As_2O (mp-8789)

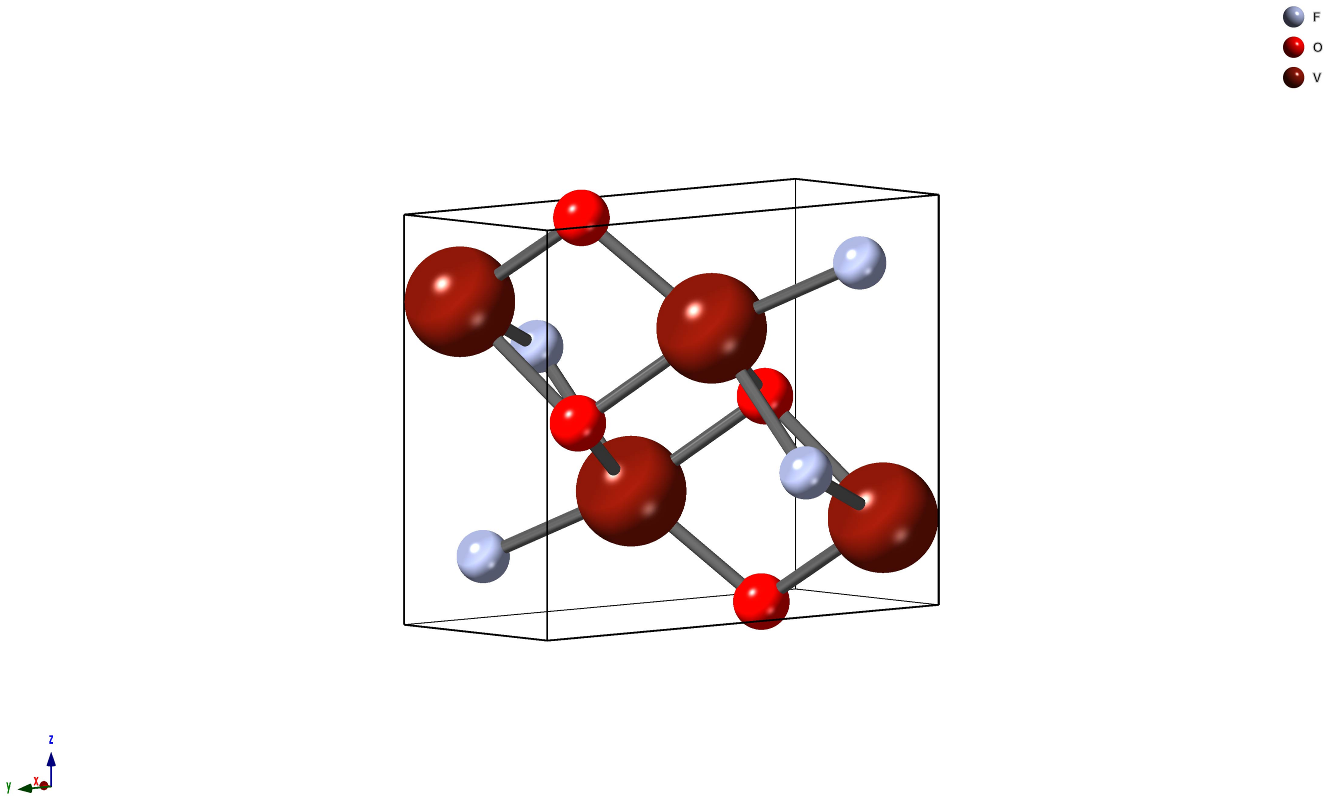

Supplementary Figure 12: Crystal structure of VOF (mp-768037)

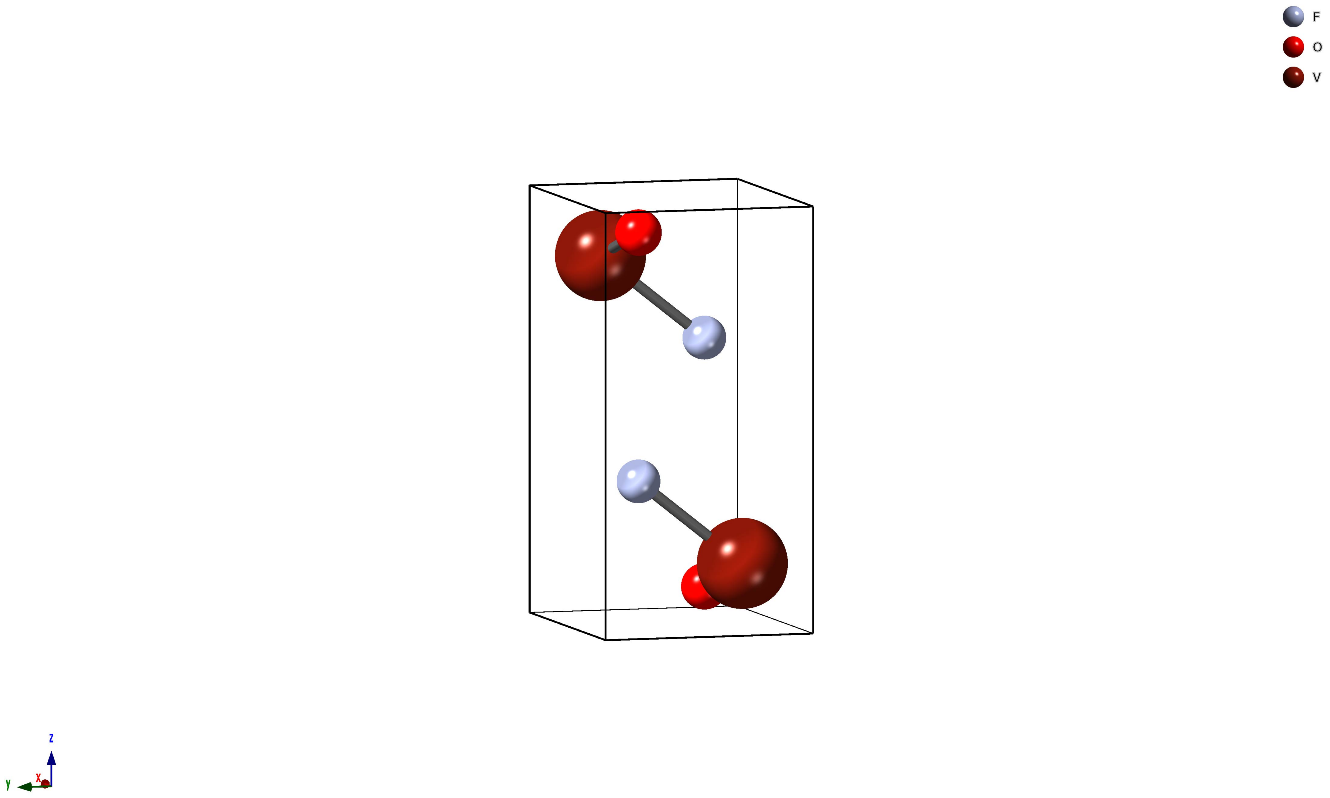

Supplementary Figure 13: Crystal structure of VOF (mp-764309)

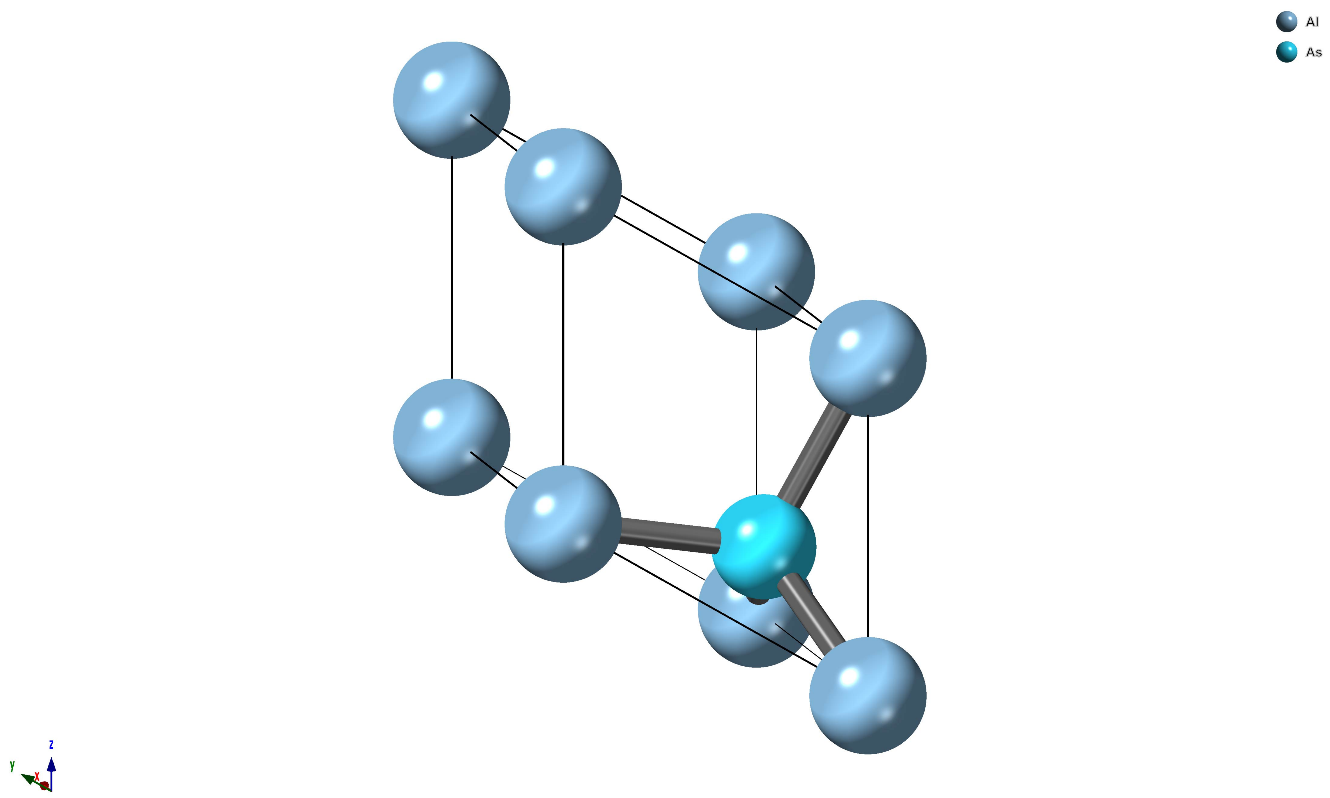

Supplementary Figure 14: Crystal structure of AlAs (mp-2172)

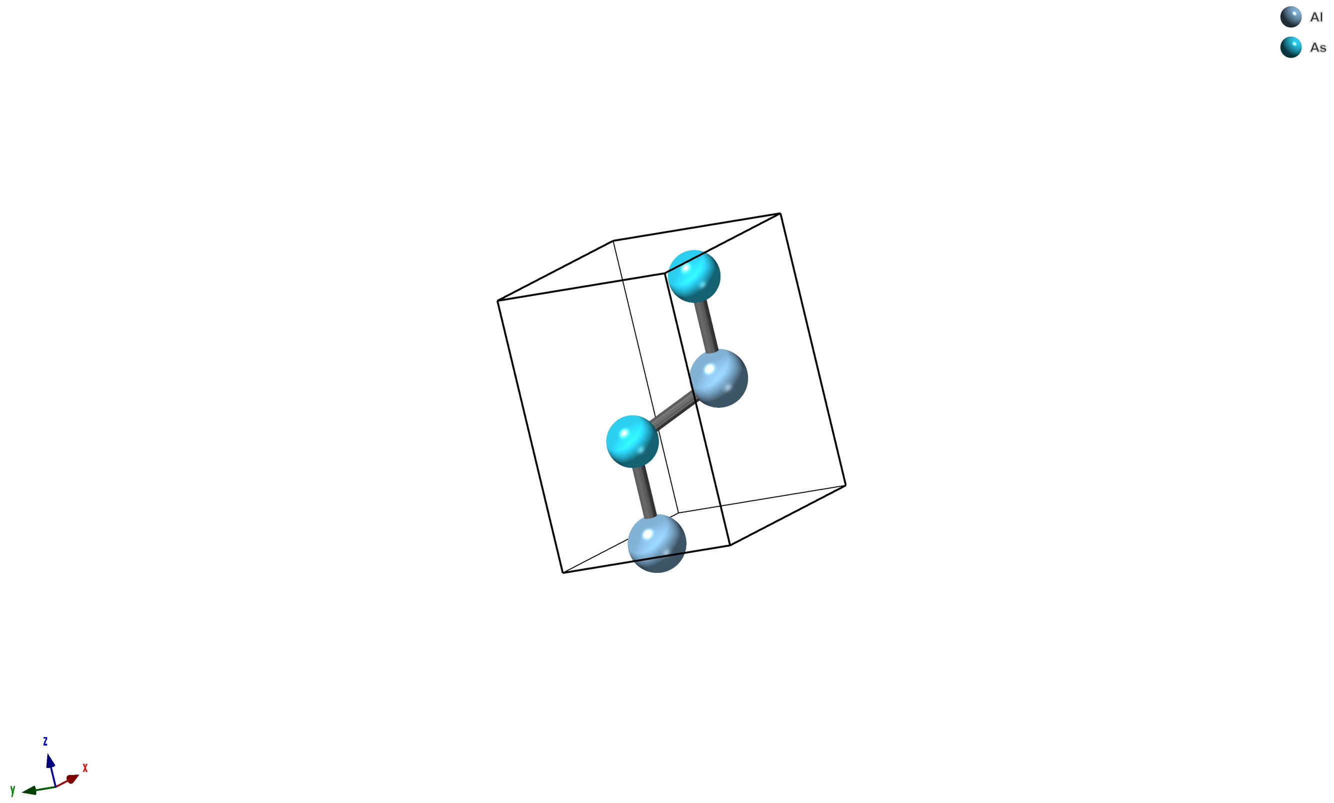

Supplementary Figure 15: Crystal structure of AlAs (mp-8881)

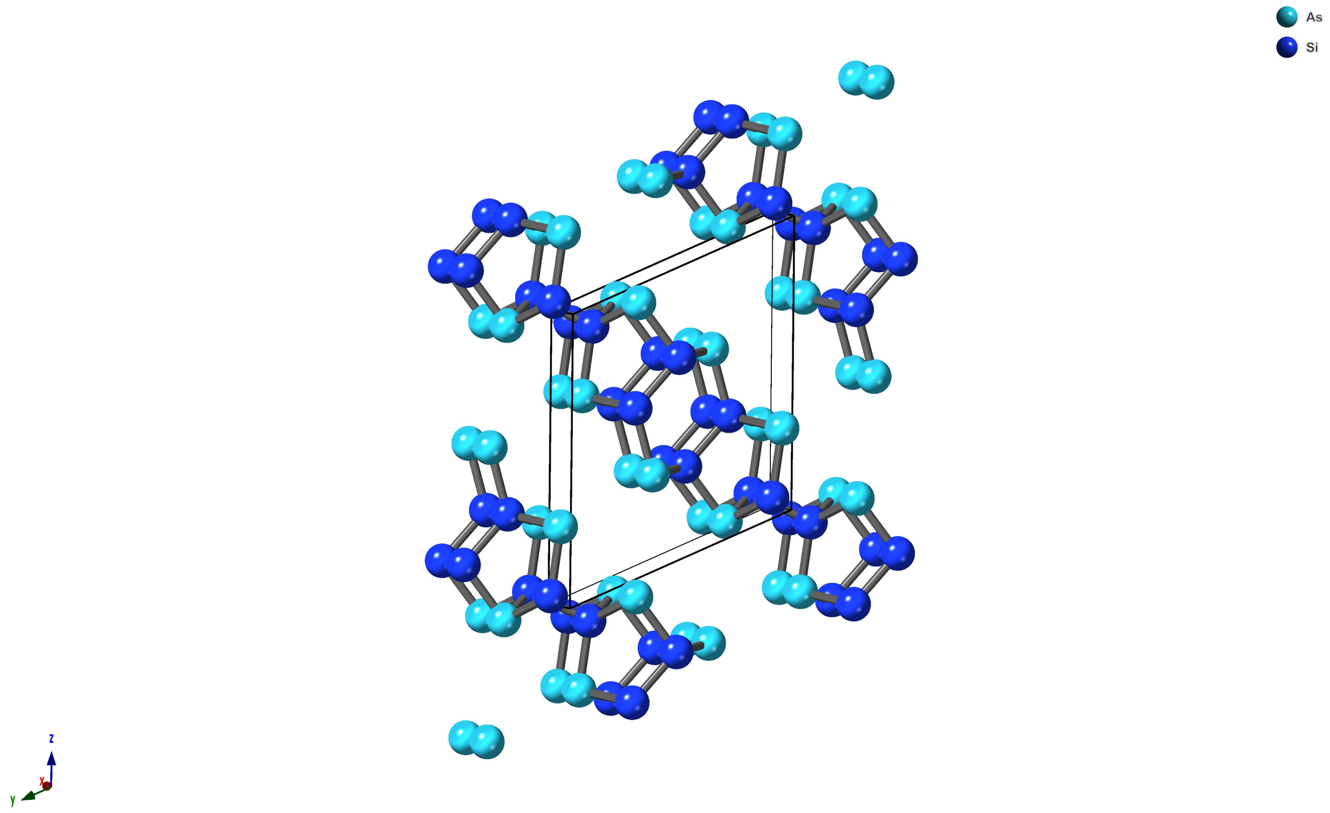

Supplementary Figure 16: Crystal structure of SiAs (mp-1863)

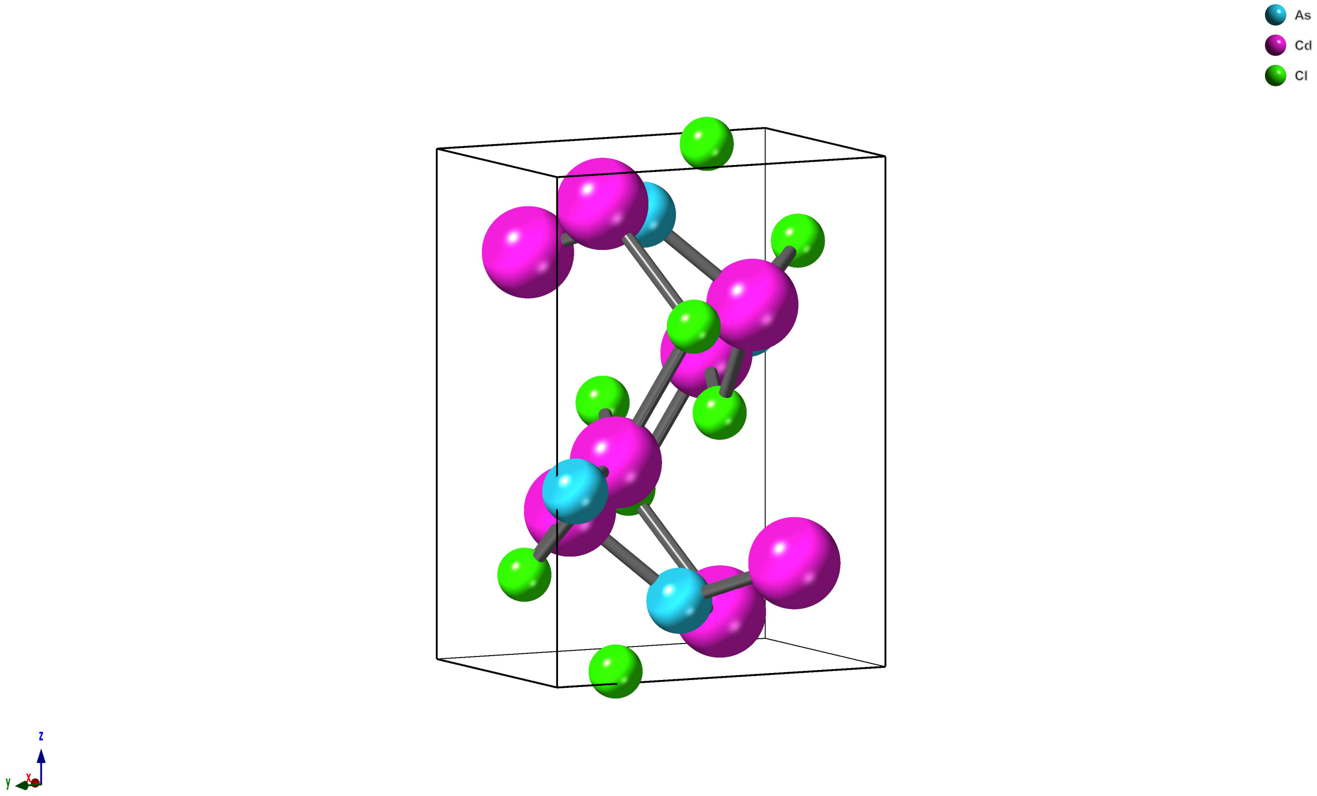

Supplementary Figure 17: Crystal structure of Cd_2AsCl_2 (mp-27776)

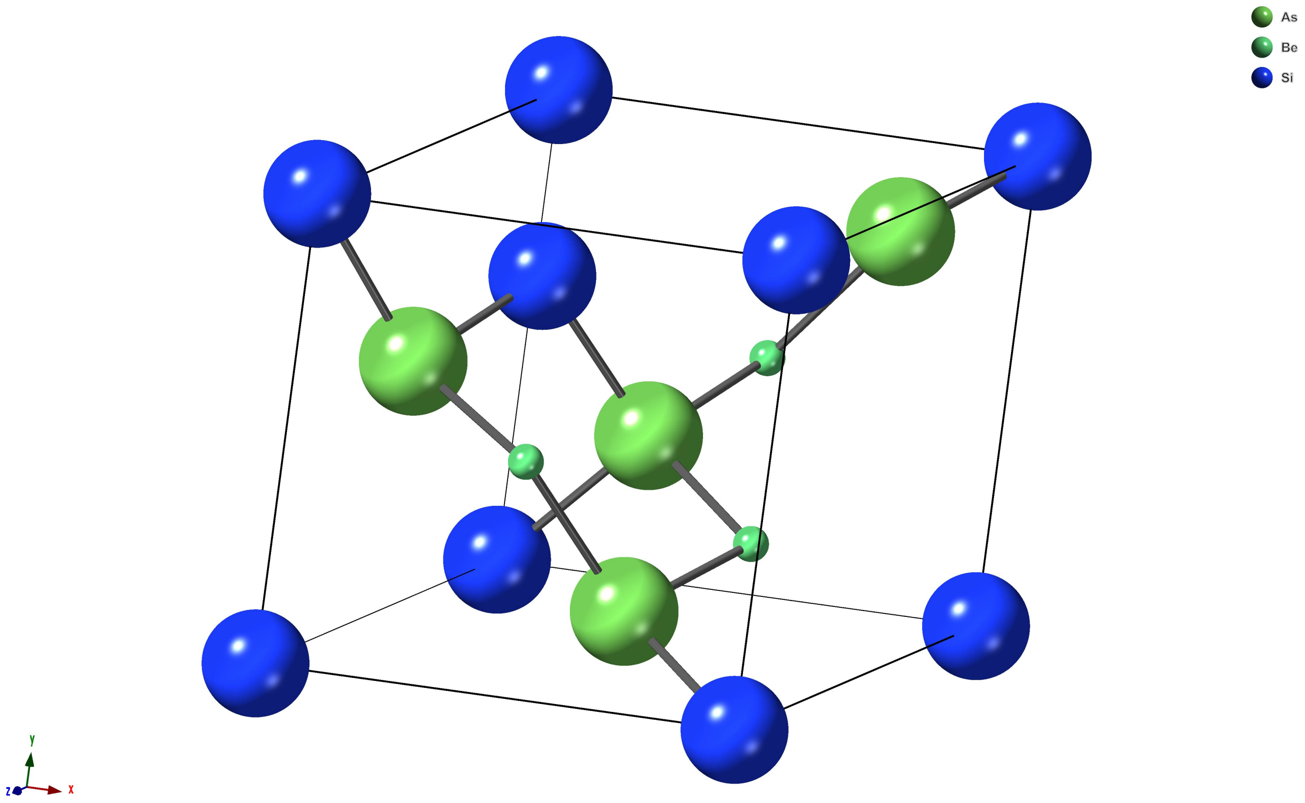

Supplementary Figure 18: Crystal structure of $BeSiAs_2$ (mp-1009087)

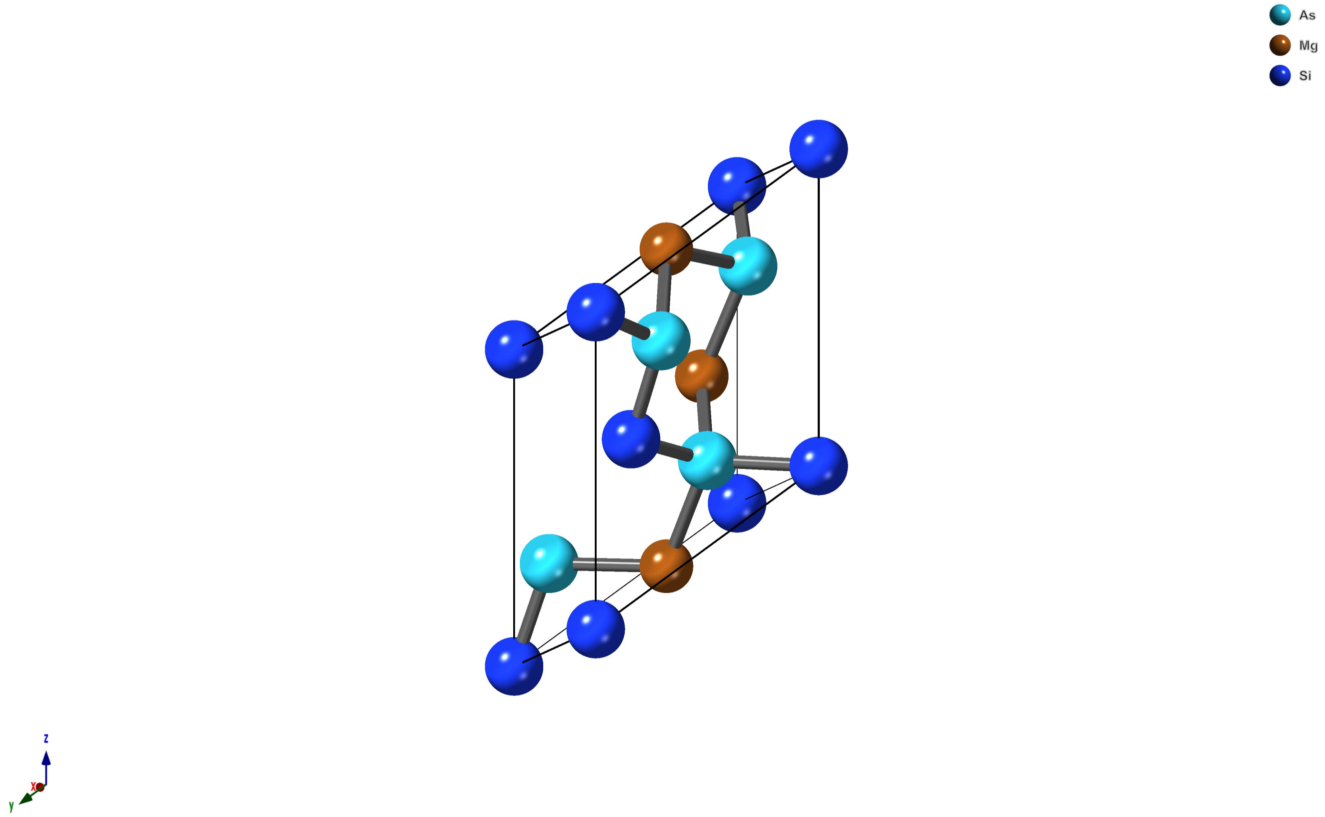

Supplementary Figure 19: Crystal structure of $MgSiAs_2$ (mp-1016197)

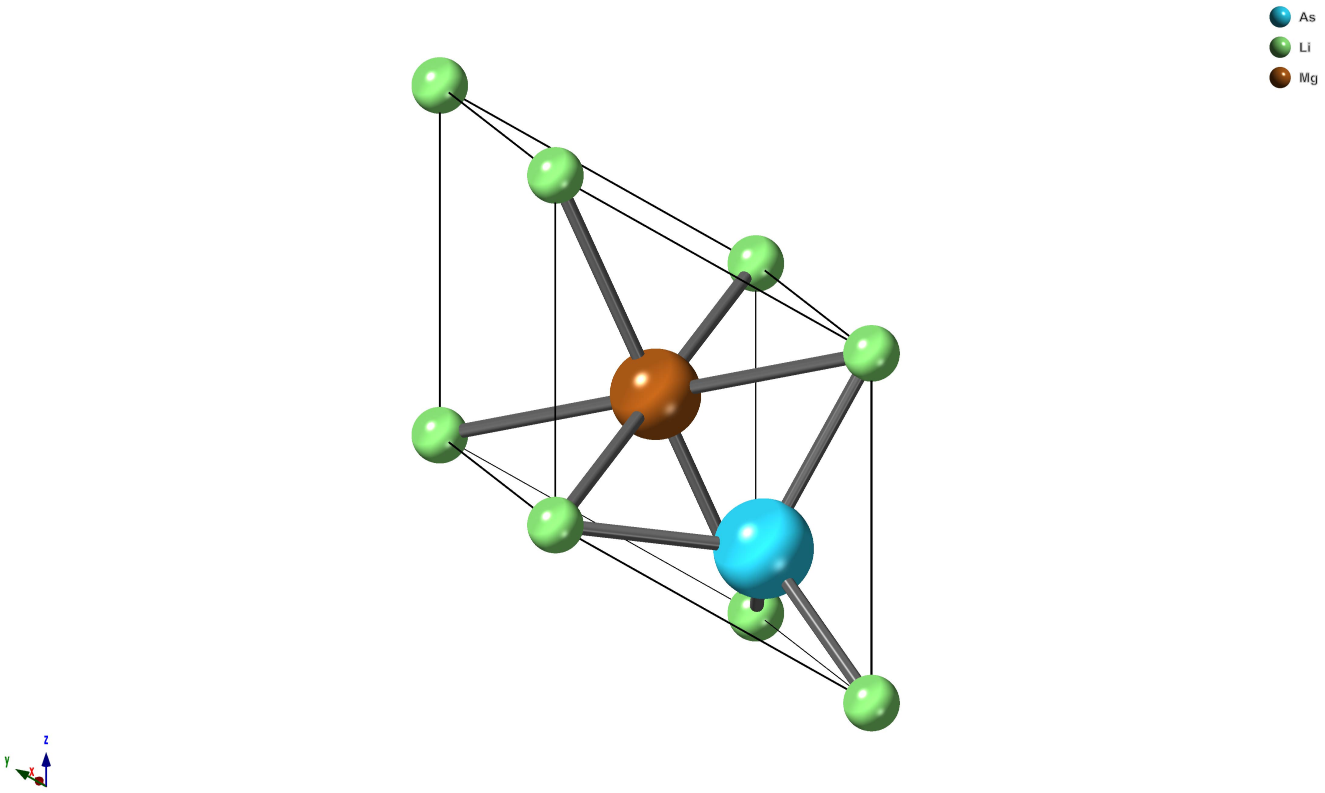

Supplementary Figure 20: Crystal structure of LiMgAs (mp-12558)

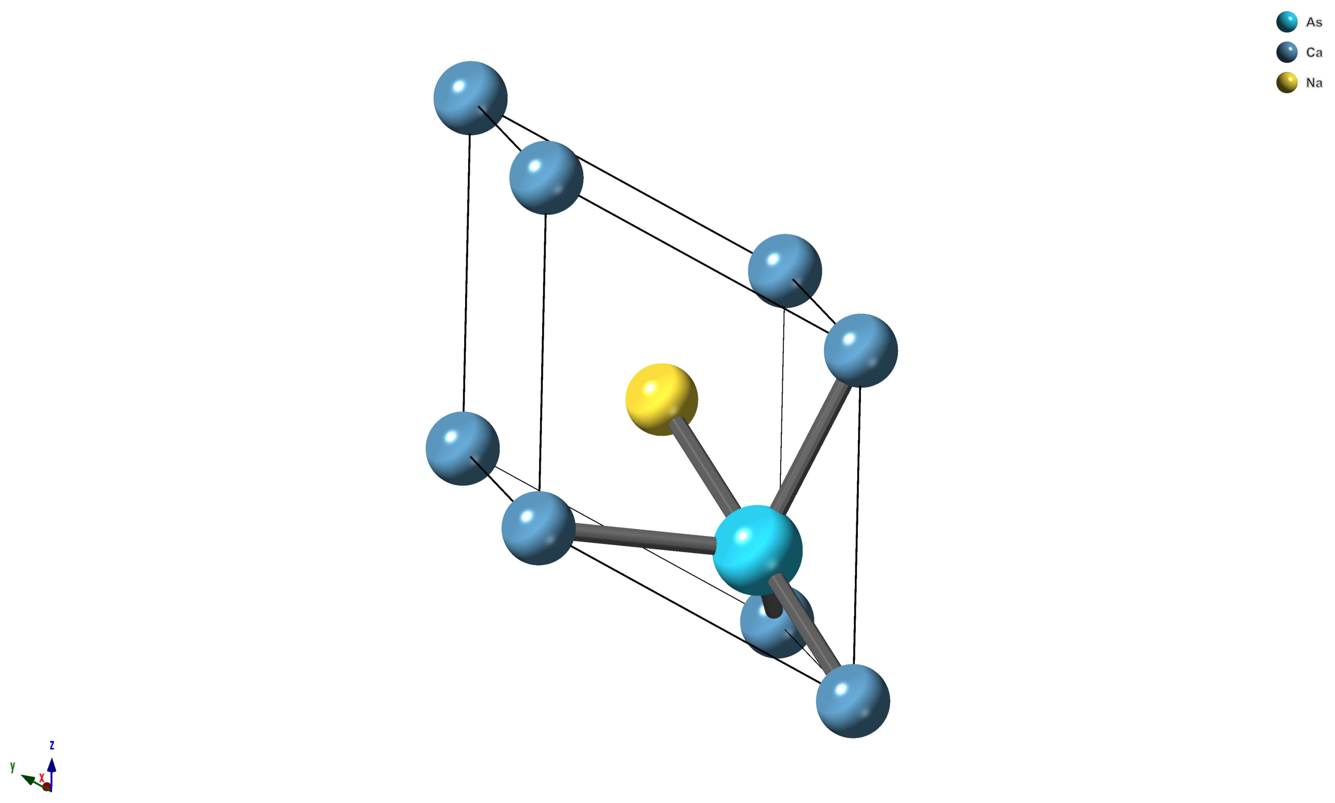

Supplementary Figure 21: Crystal structure of NaCaAs (mp-961685)

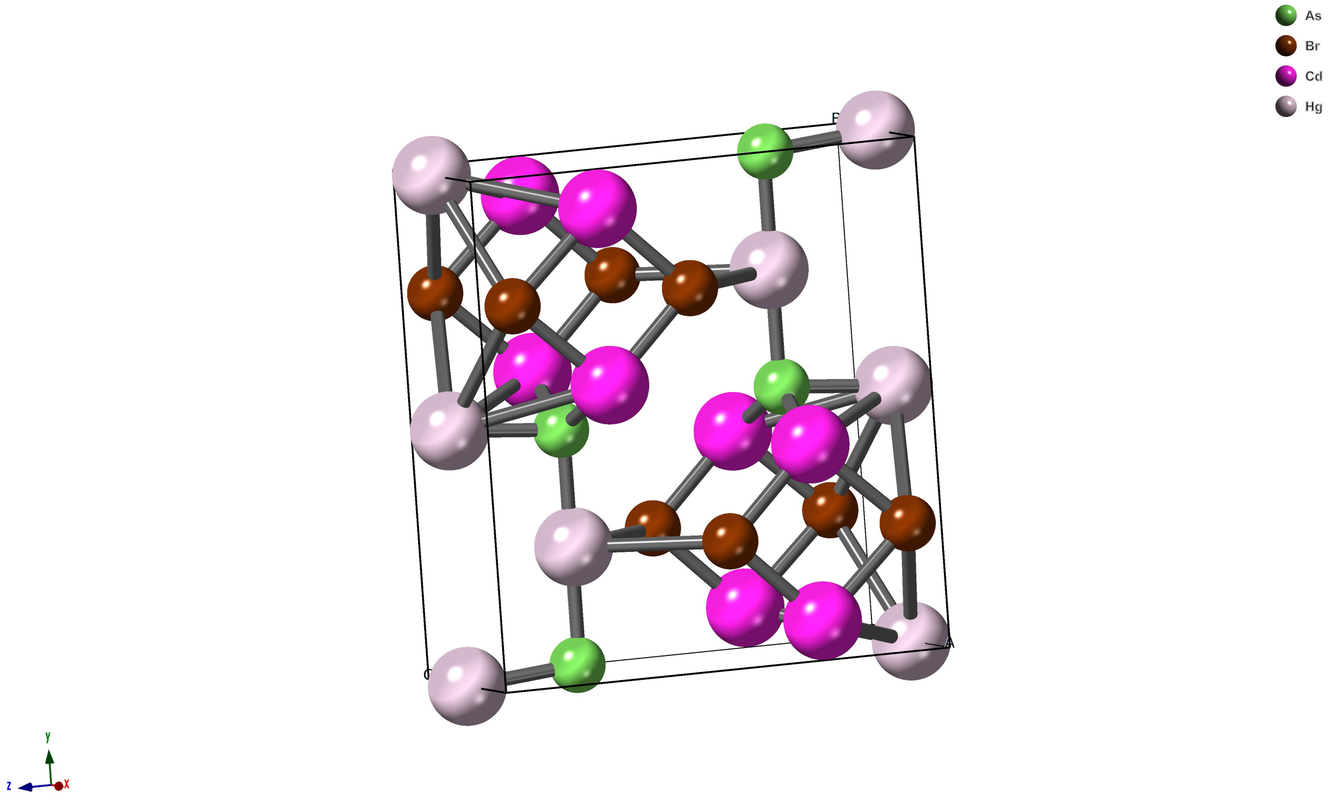

Supplementary Figure 22: Crystal structure of CdHgAsBr (mp-569454)

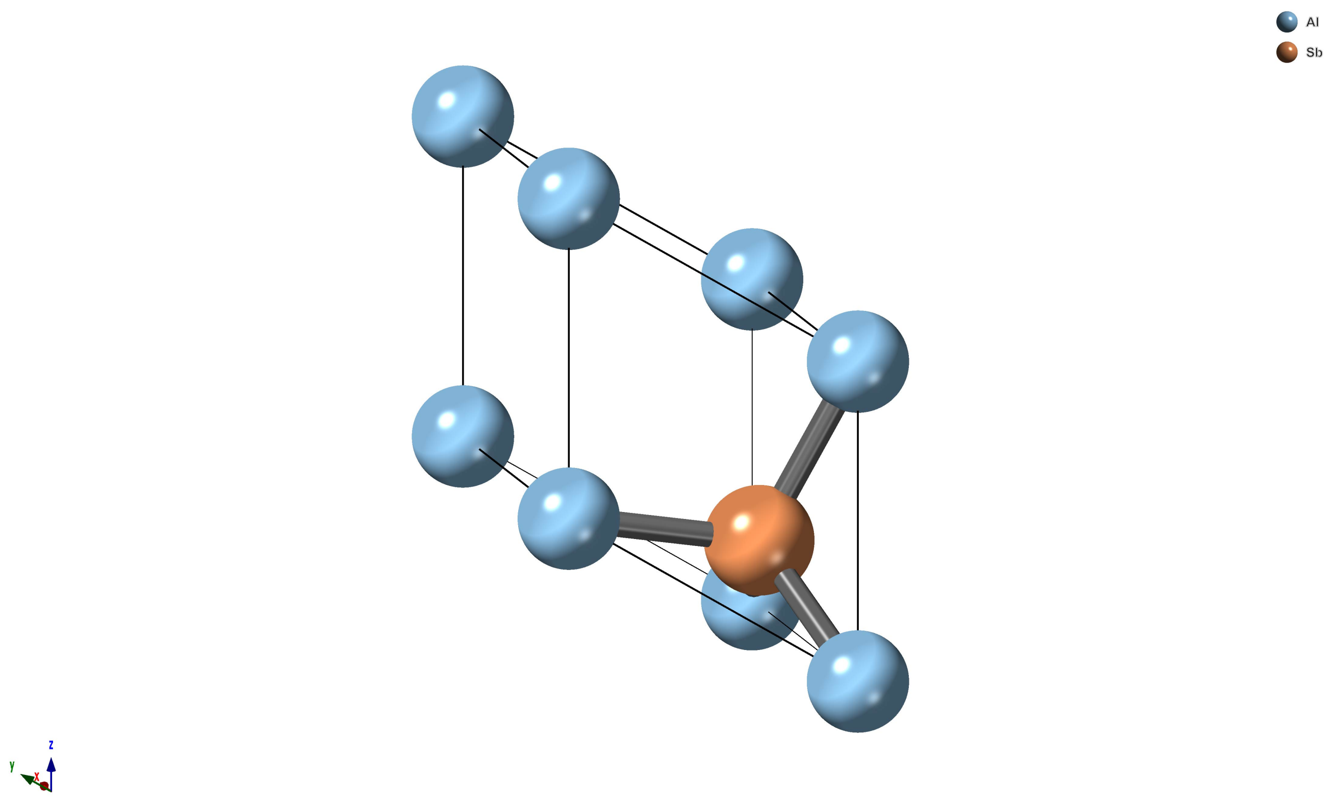

Supplementary Figure 23: Crystal structure of AlSb (mp-2624)

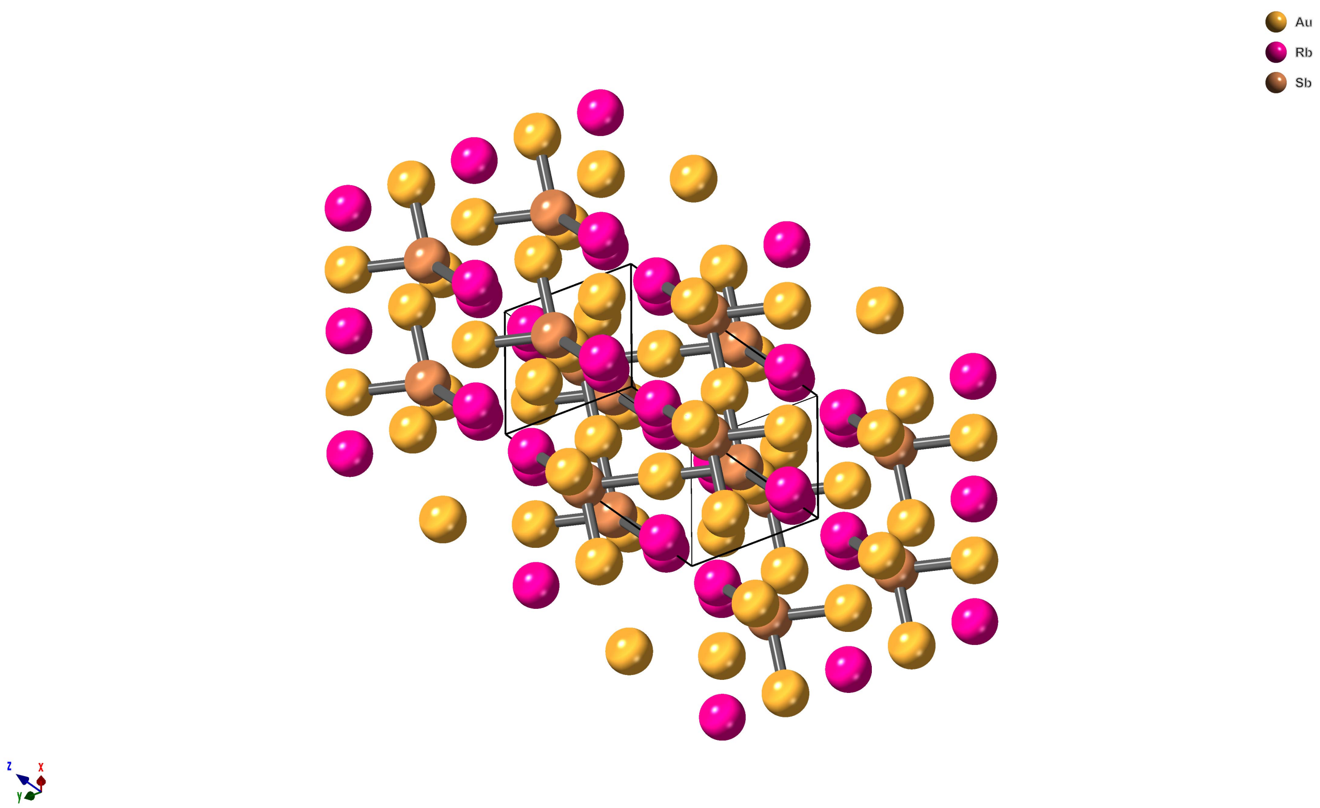

Supplementary Figure 24: Crystal structure of $Rb_3Sb_2Au_3$ (mp-9274)

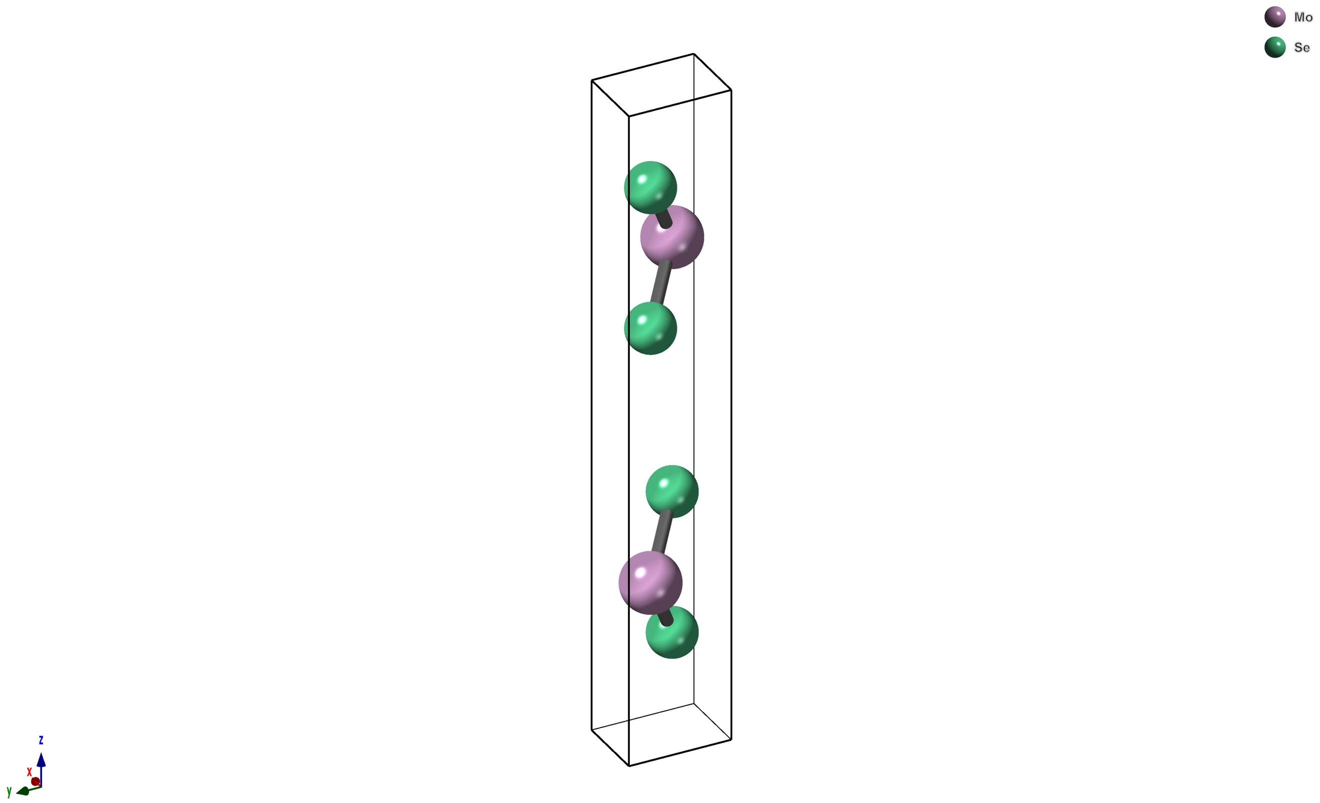

Supplementary Figure 25: Crystal structure of $MoSe_2$ (mp-1634)

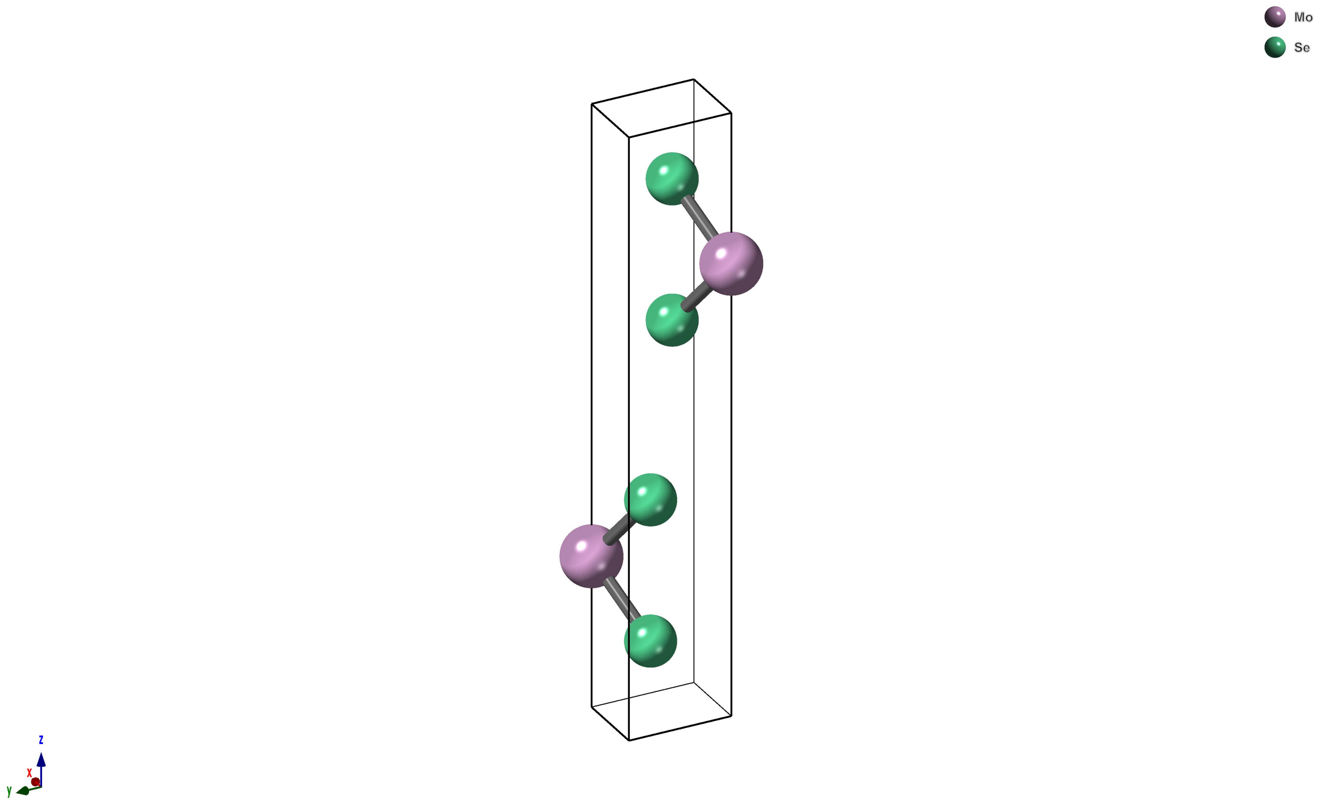

Supplementary Figure 26: Crystal structure of $MoSe_2$ (mp-1018807)

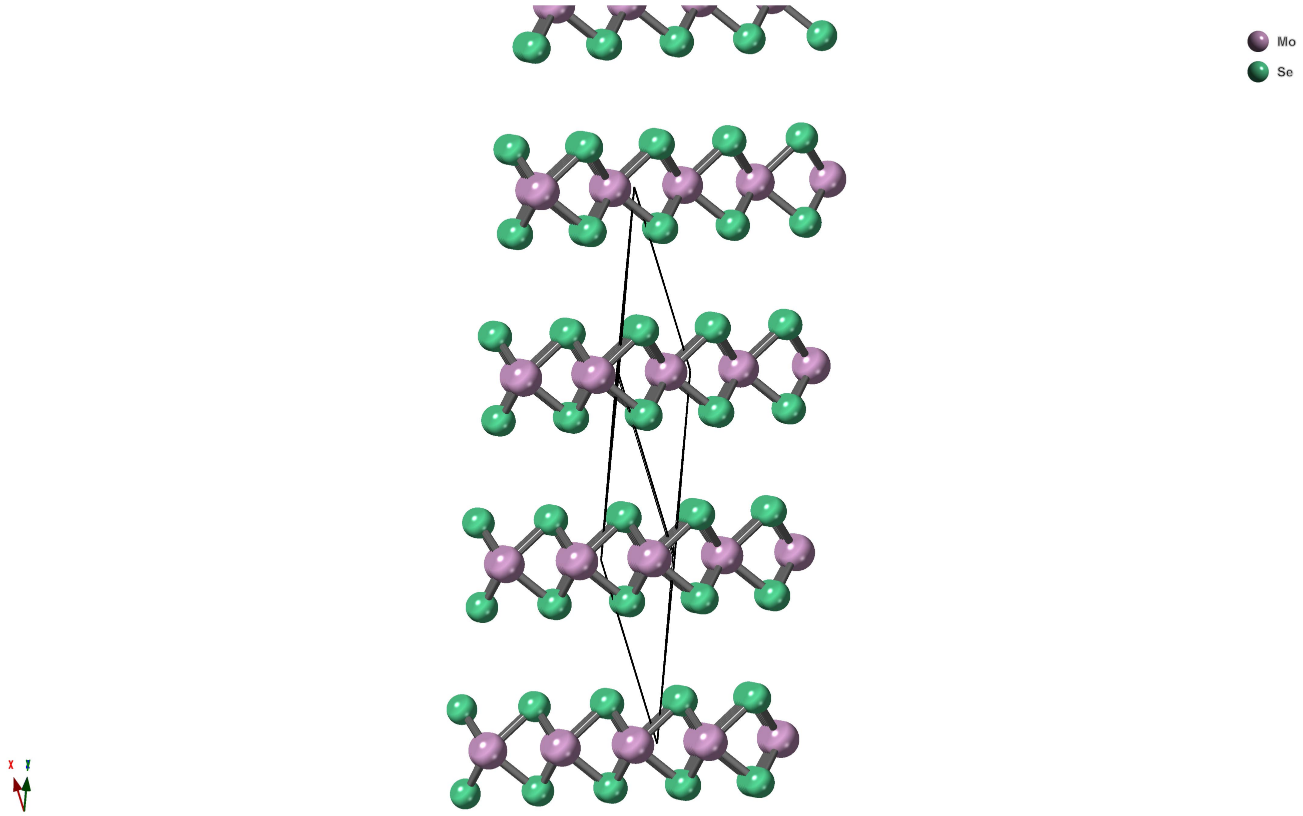

Supplementary Figure 27: Crystal structure of $MoSe_2$ (mp-7581)

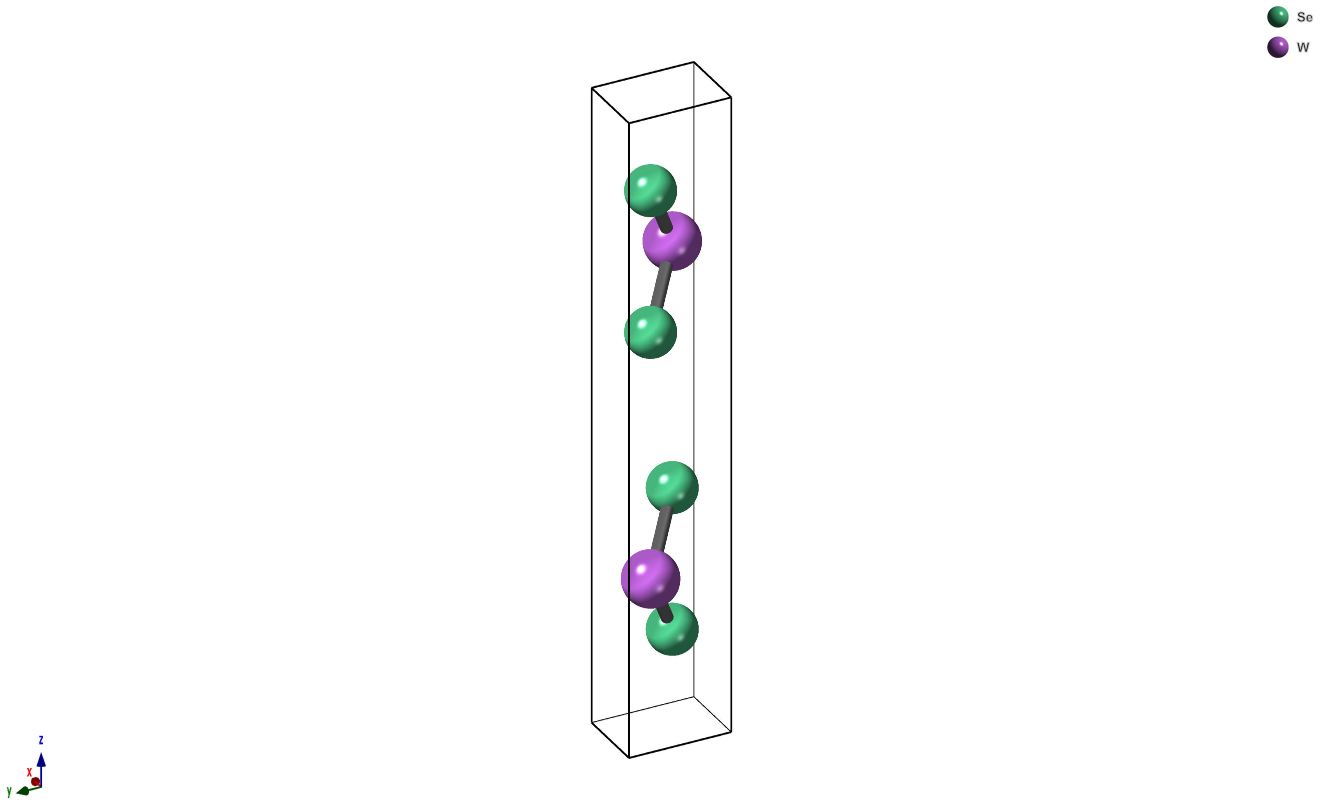

Supplementary Figure 28: Crystal structure of WSe_2 (mp-1821)

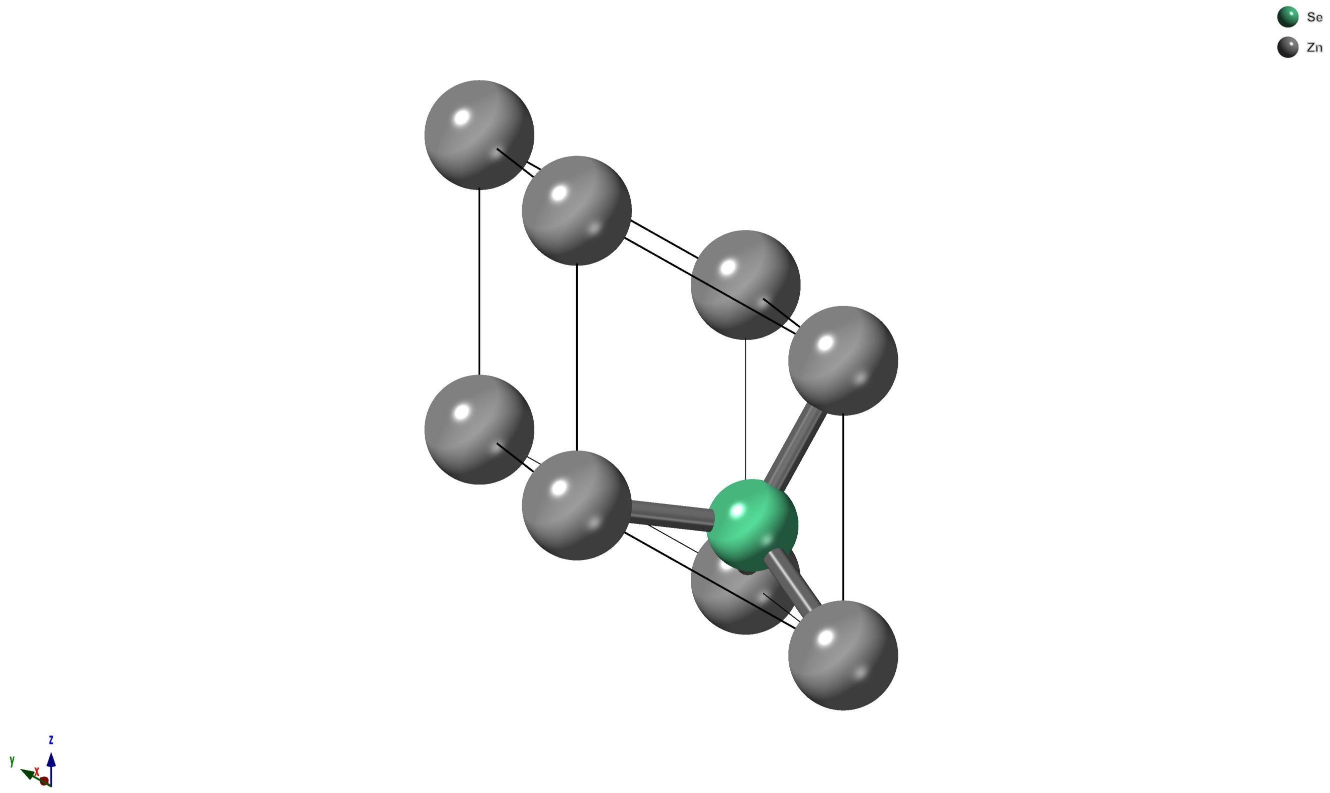

Supplementary Figure 29: Crystal structure of ZnSe (mp-1190)

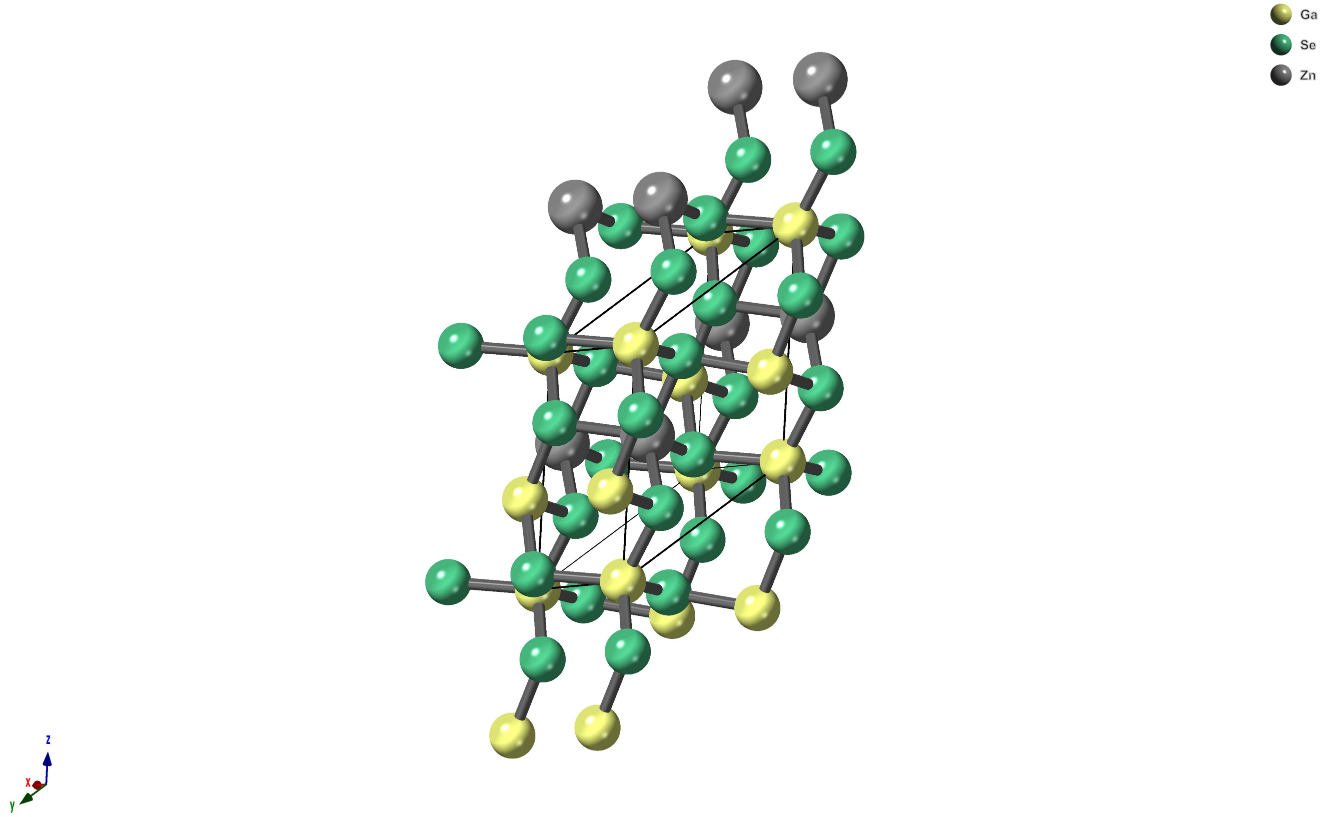

Supplementary Figure 30: Crystal structure of $Zn(GaSe_2)_2$ (mp-15776)

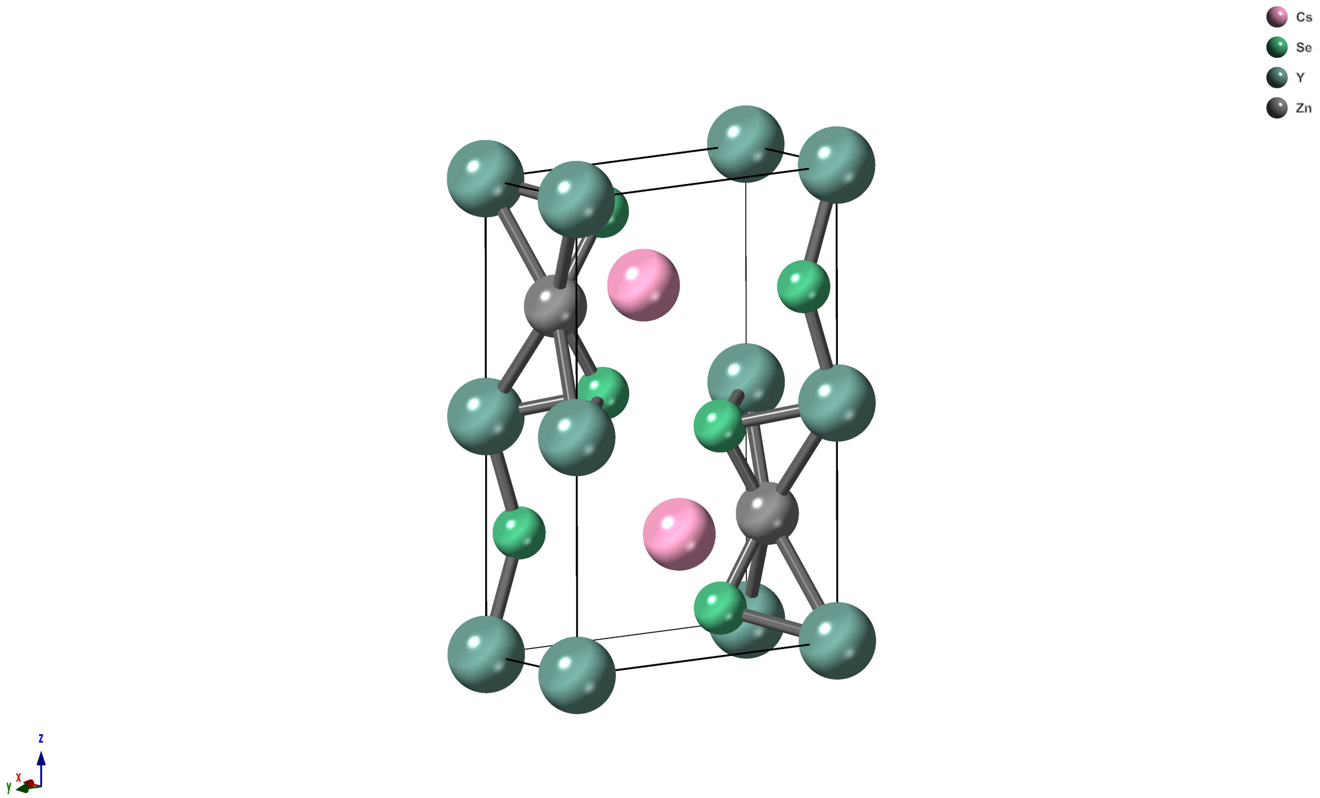

Supplementary Figure 31: Crystal structure of $CsYZnSe_3$ (mp-574620)

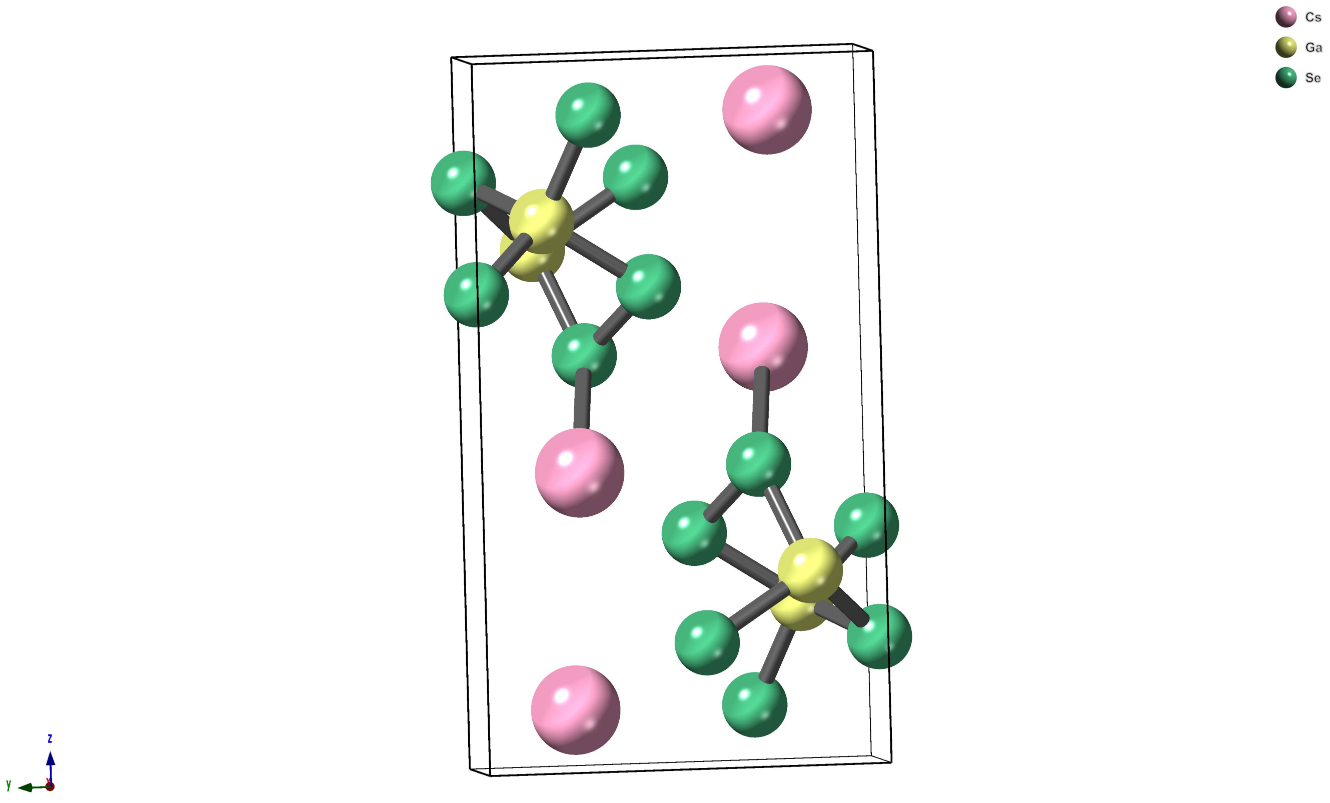

Supplementary Figure 32: Crystal structure of $CsGaSe_3$ (mp-510283)

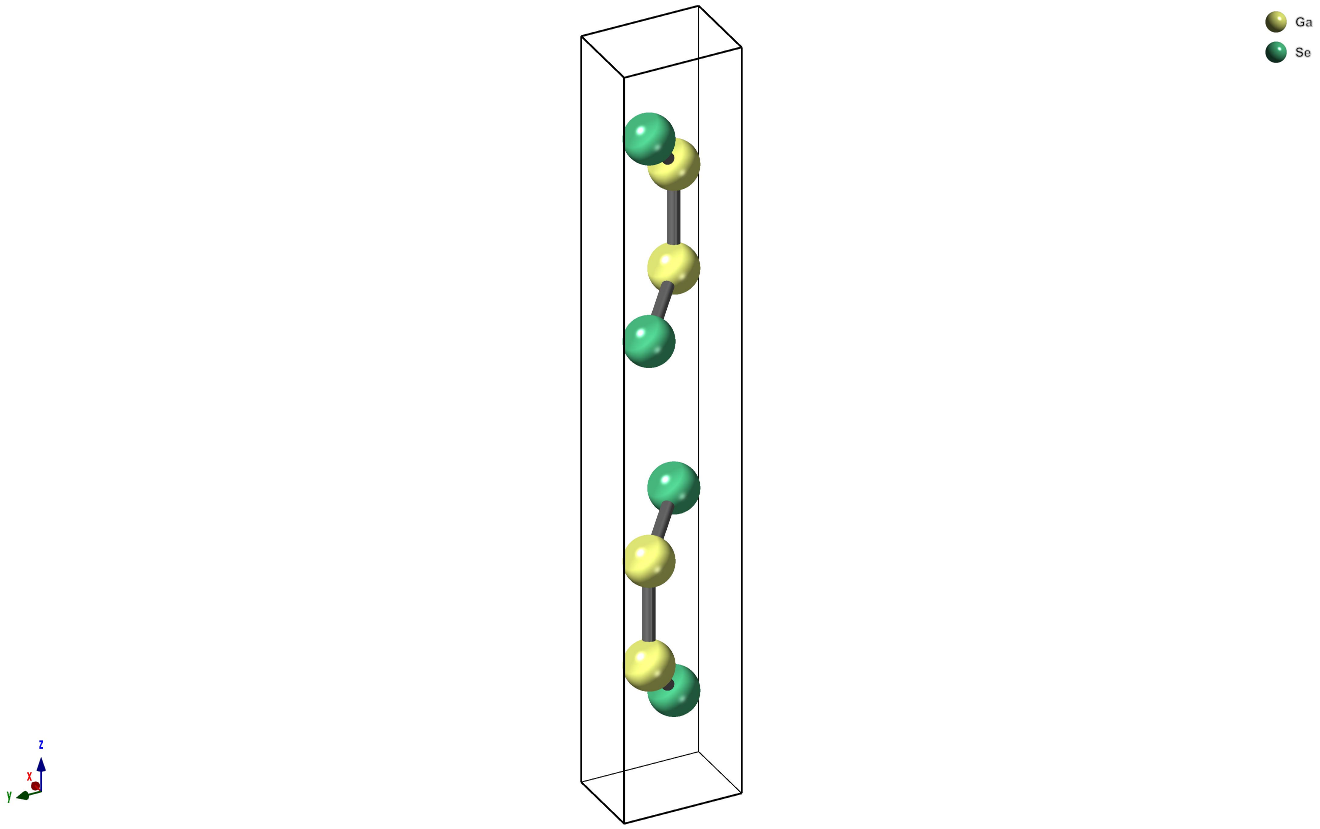

Supplementary Figure 33: Crystal structure of GaSe (mp-1943)

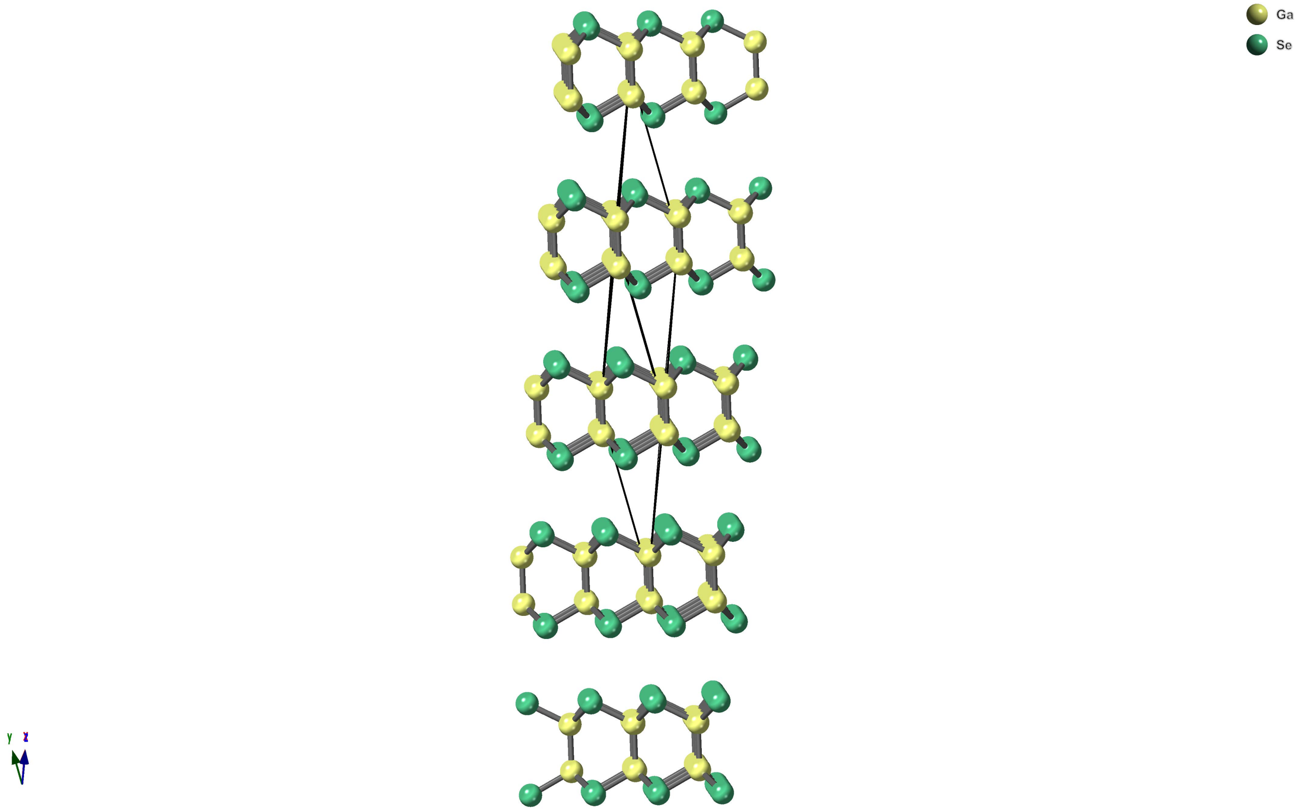

Supplementary Figure 34: Crystal structure of GaSe (mp-11342)

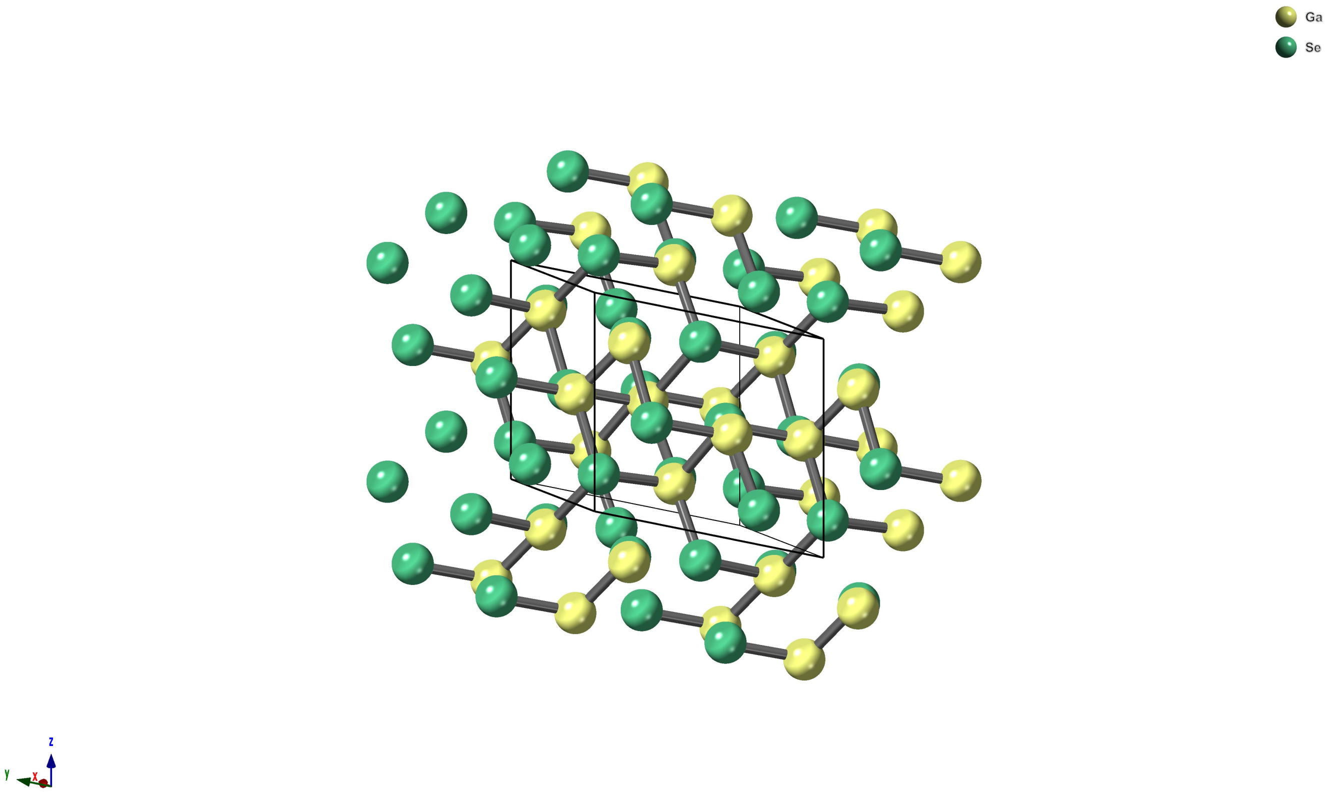

Supplementary Figure 35: Crystal structure of Ga_2Se_3 (mp-1340)

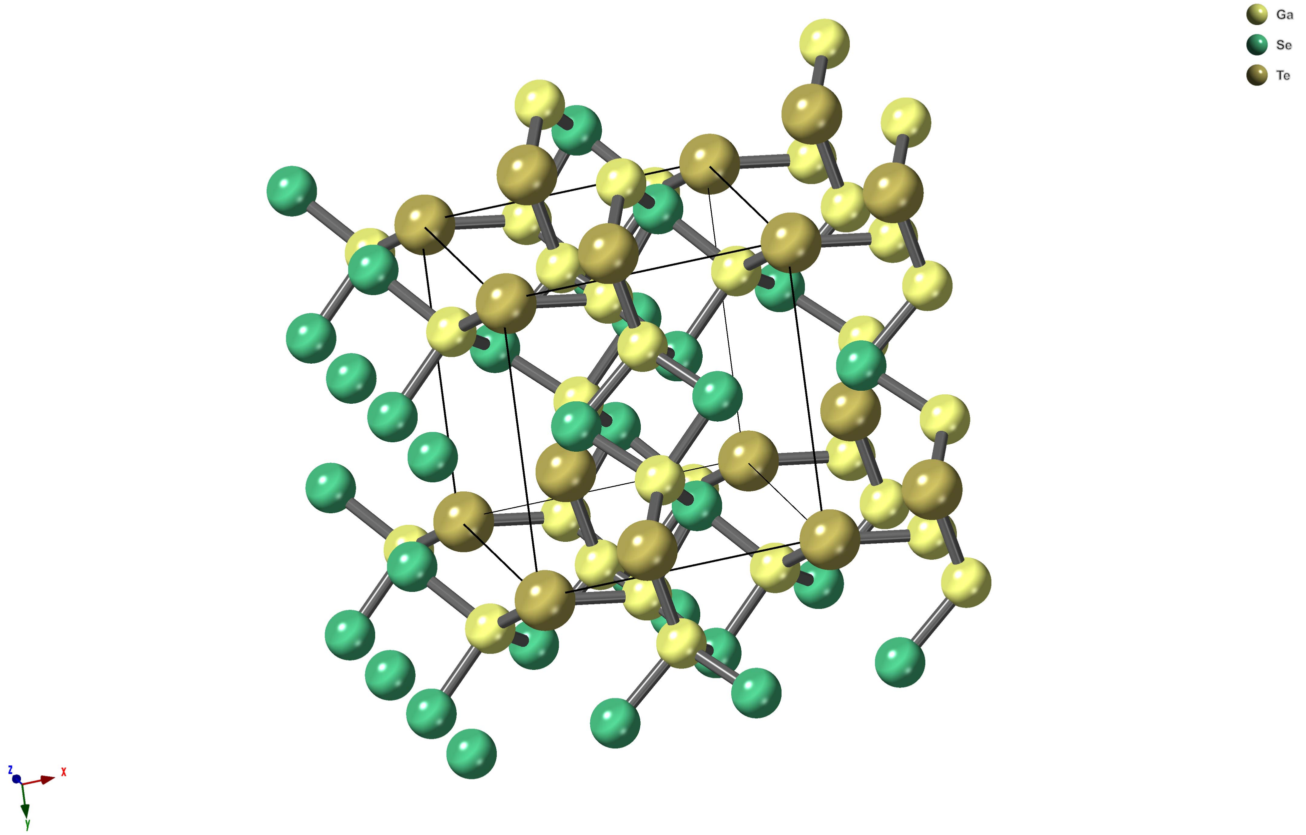

Supplementary Figure 36: Crystal structure of Ga_2TeSe_2 (mp-28423)

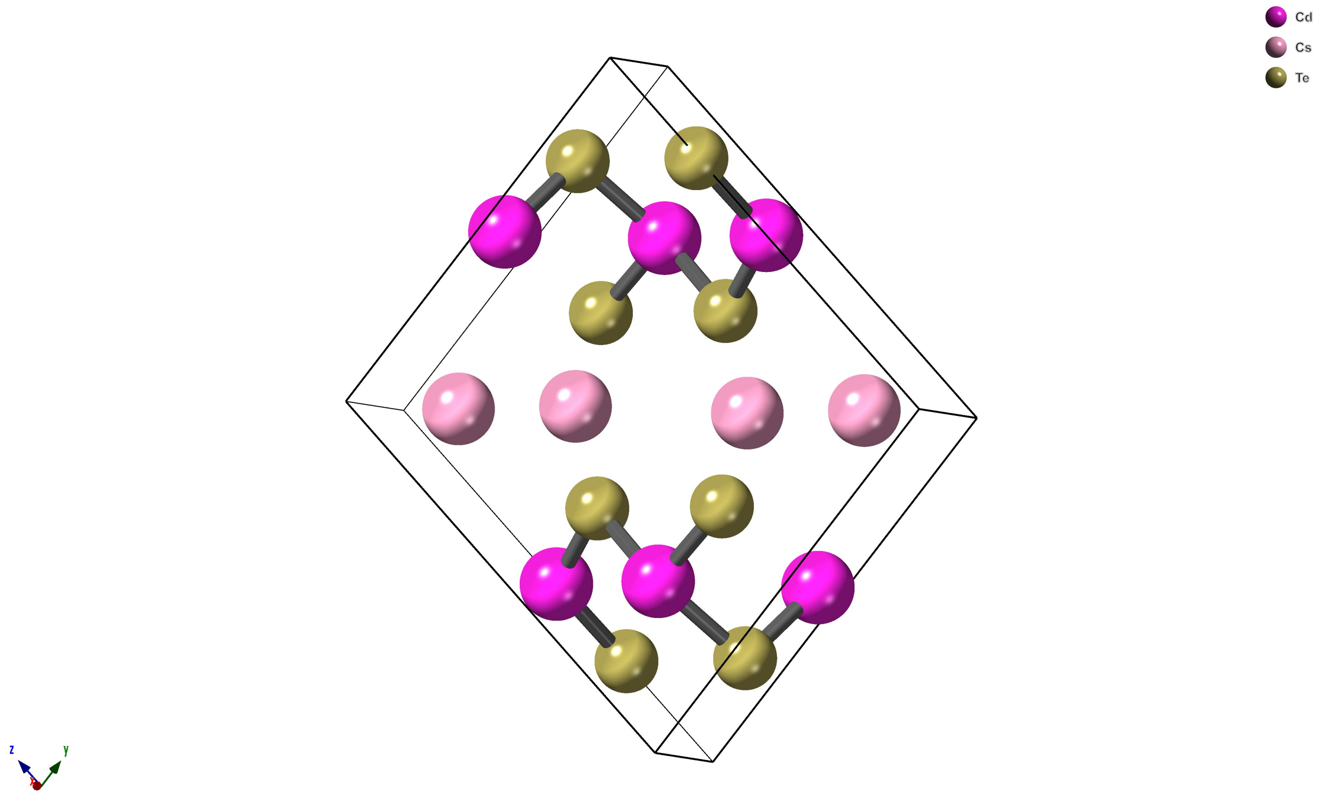

Supplementary Figure 37: Crystal structure of $Cs_2Cd_3Te_4$ (mp-567386)

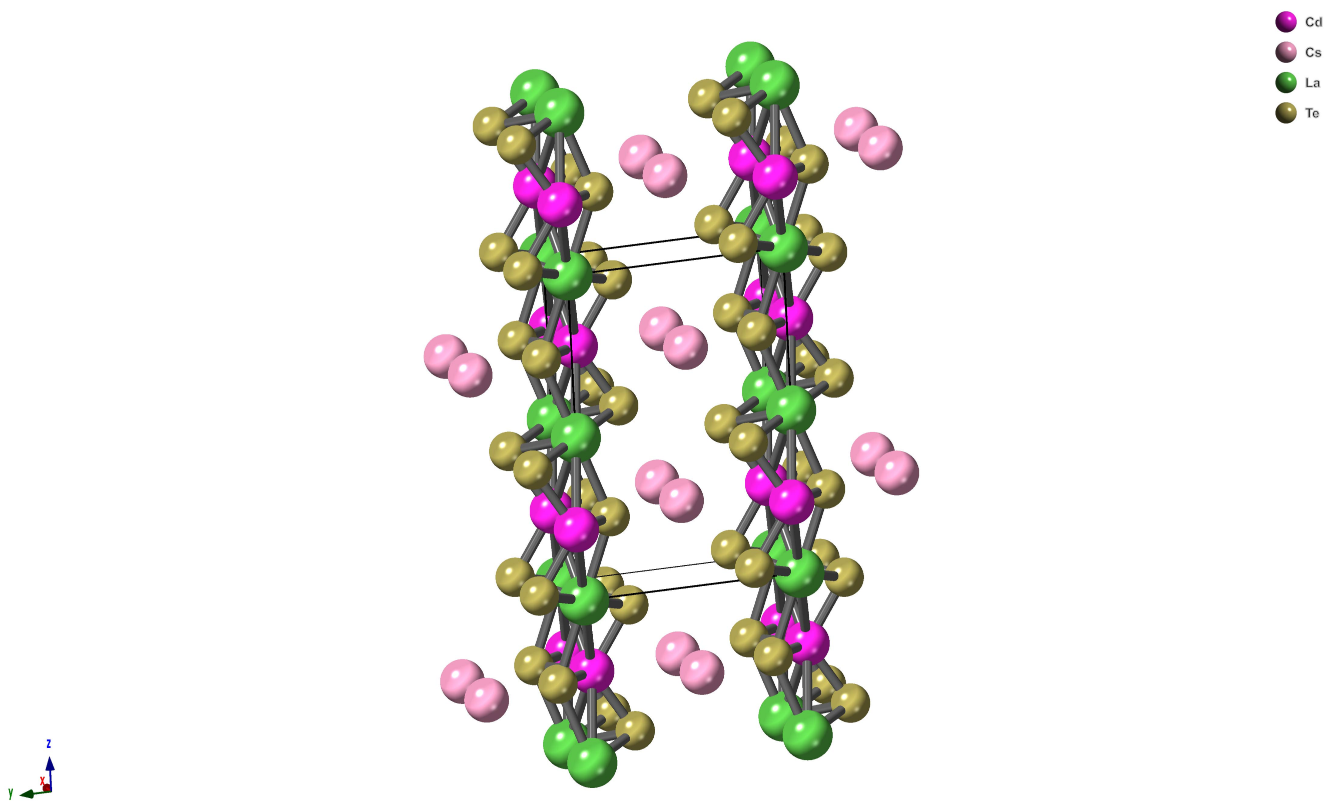

Supplementary Figure 38: Crystal structure of $CsLaCdTe_3$ (mp-12491)

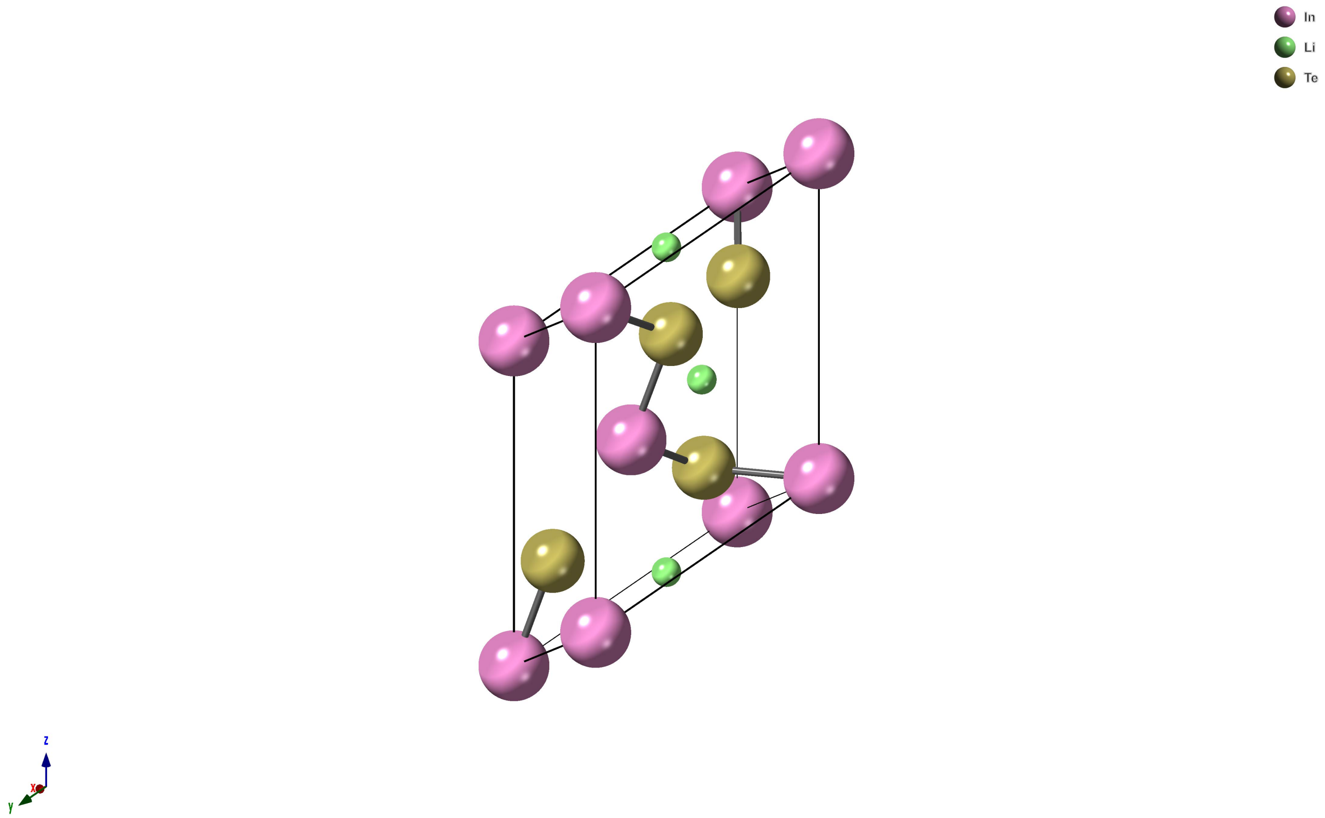

Supplementary Figure 39: Crystal structure of $LiInTe_2$ (mp-20782)

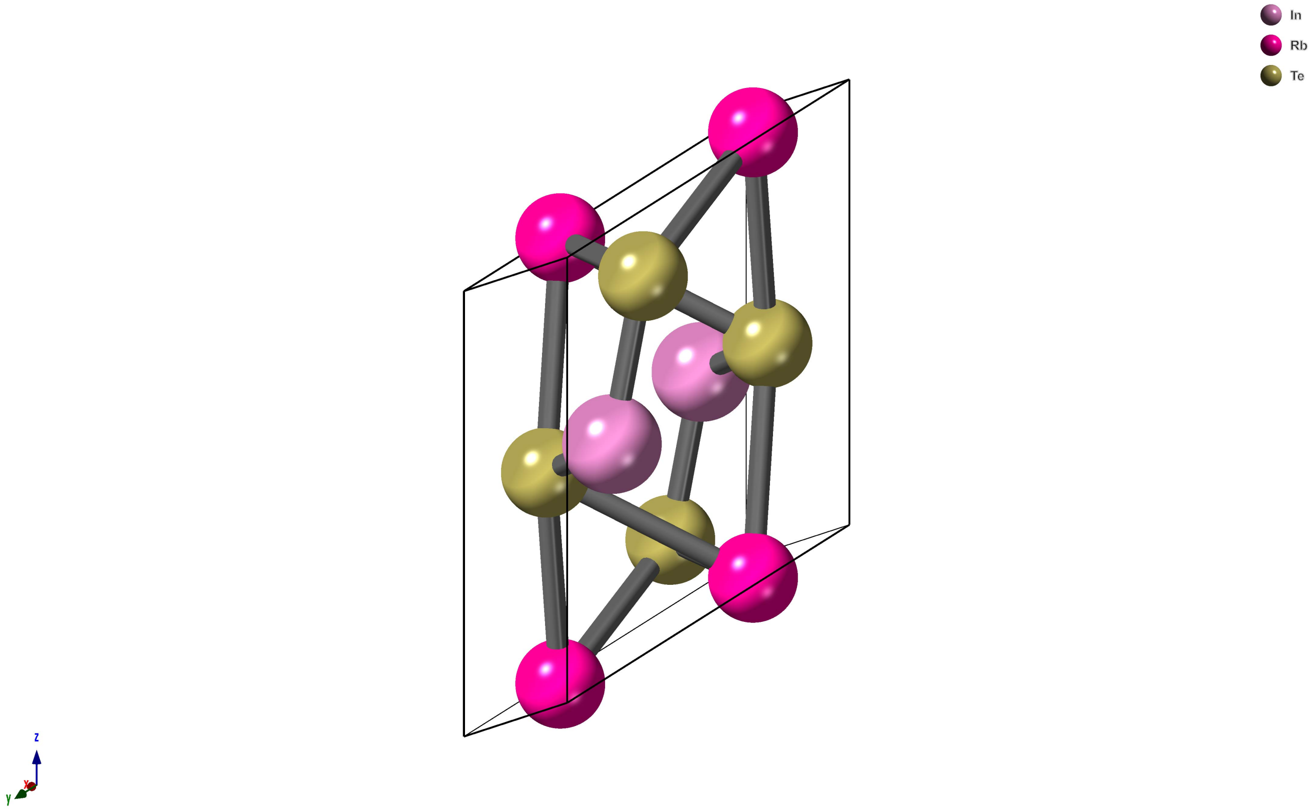

Supplementary Figure 40: Crystal structure of $RbInTe_2$ (mp-22255)

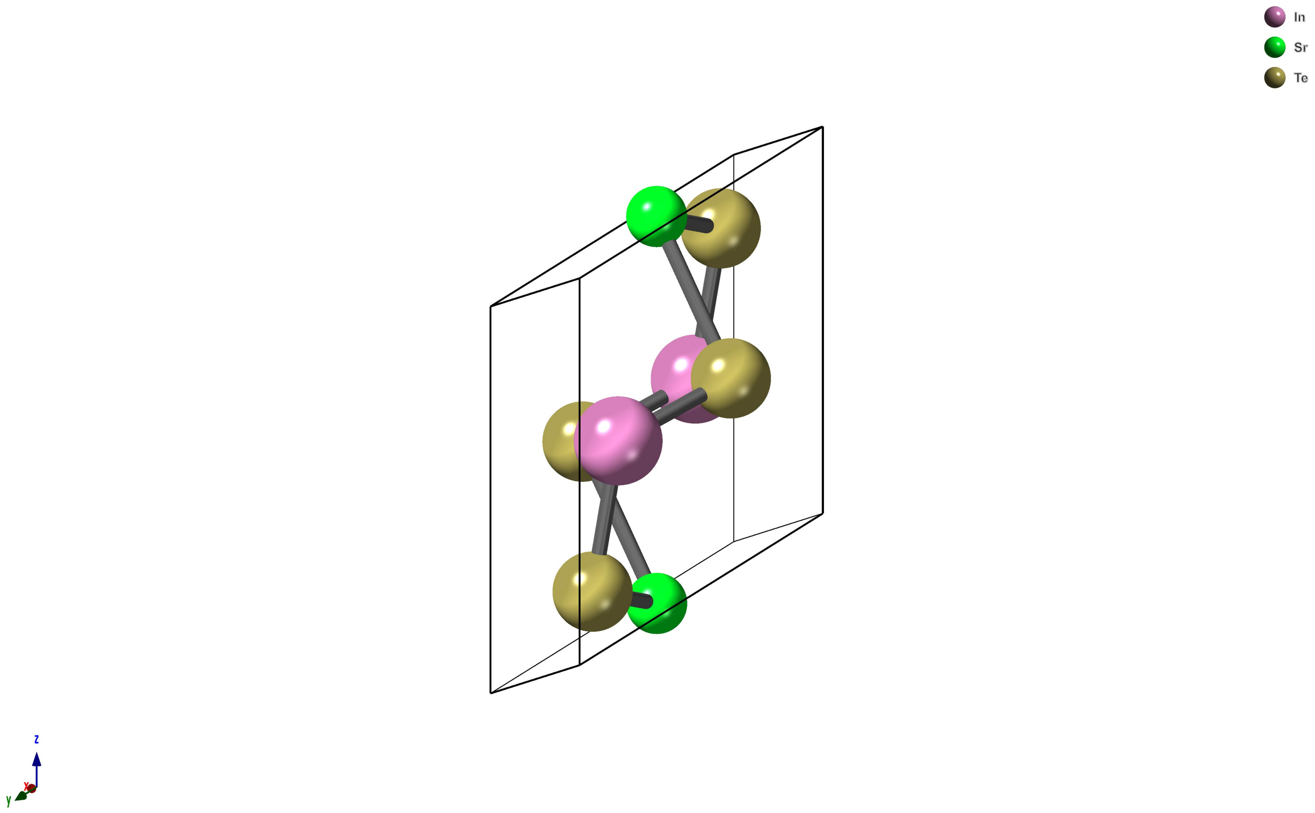

Supplementary Figure 41: Crystal structure of $Sr(InTe_2)_2$ (mp-35663)

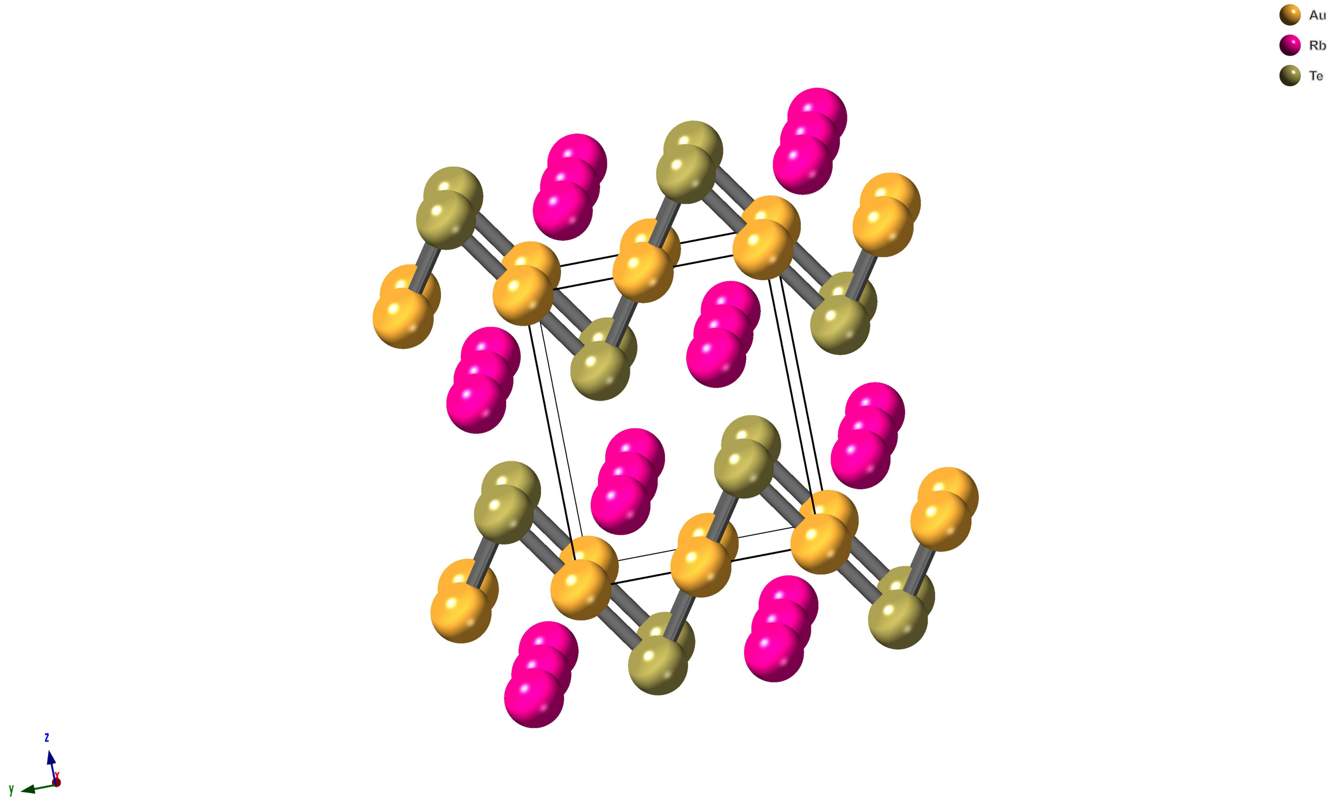

Supplementary Figure 42: Crystal structure of RbTeAu (mp-9008)

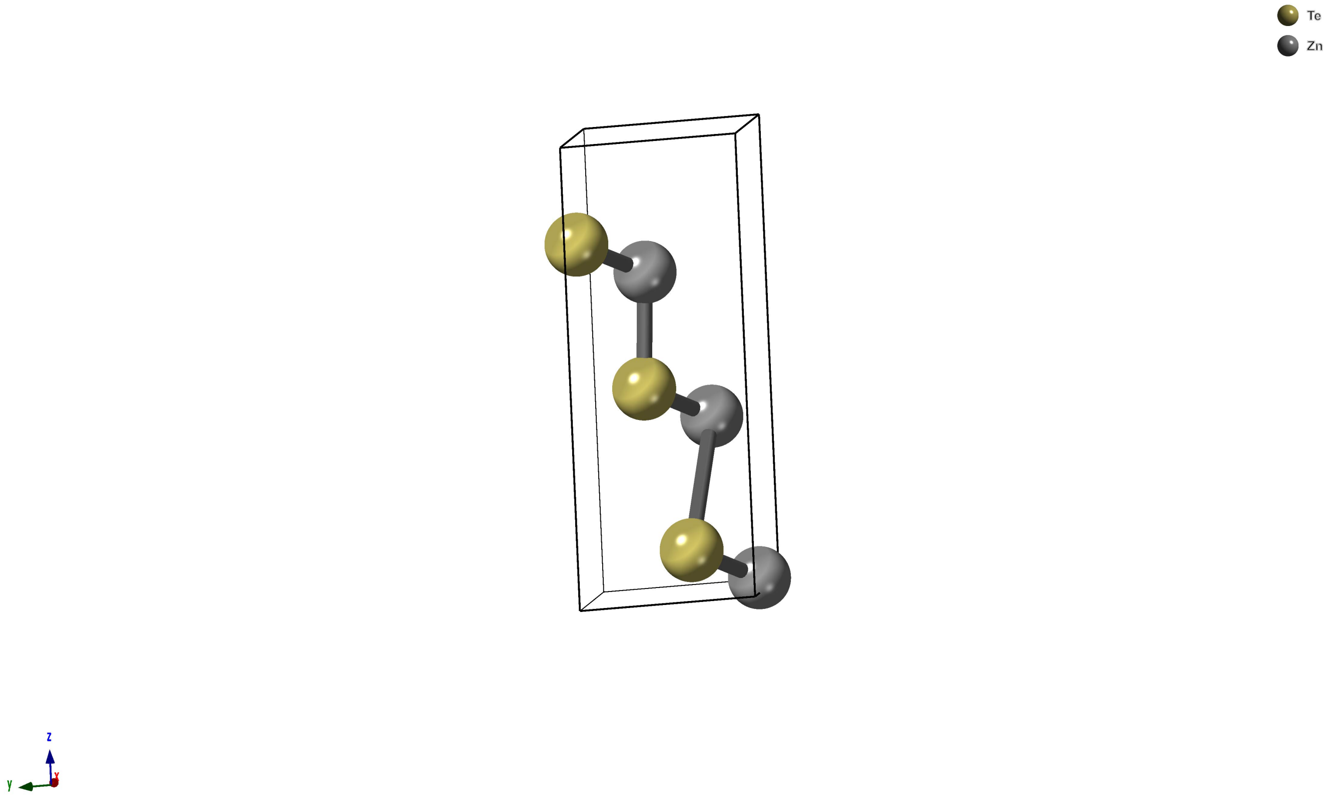

Supplementary Figure 43: Crystal structure of ZnTe (mp-571195)

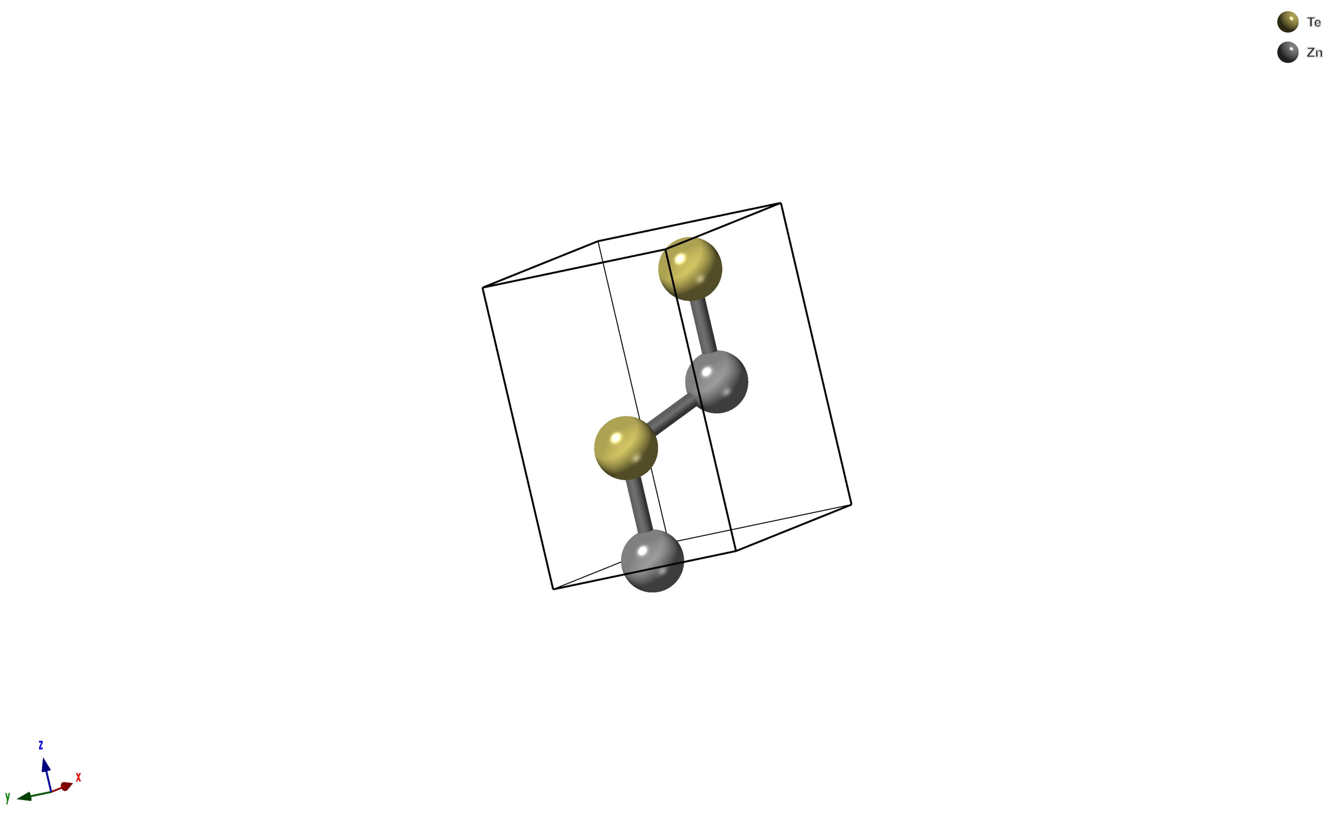

Supplementary Figure 44: Crystal structure of ZnTe (mp-8884)

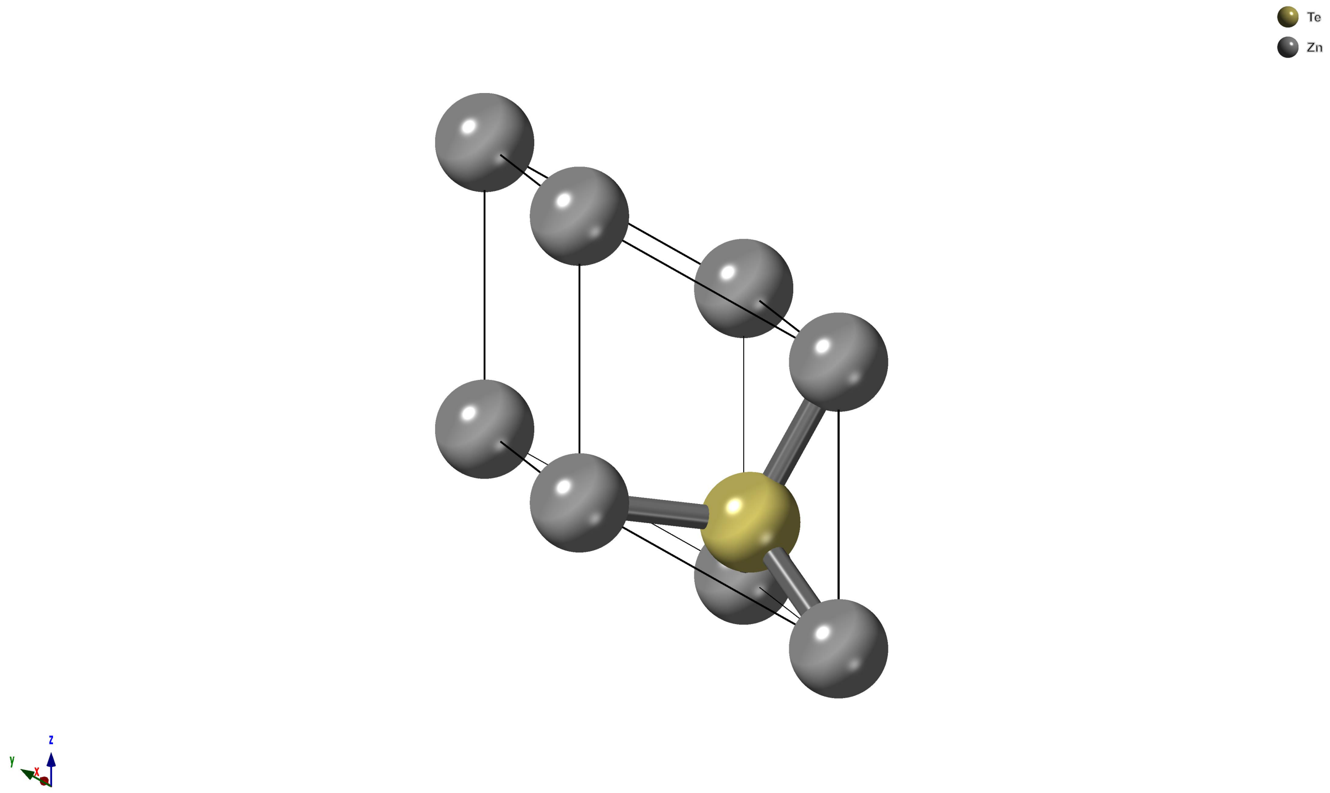

Supplementary Figure 45: Crystal structure of ZnTe (mp-2176)

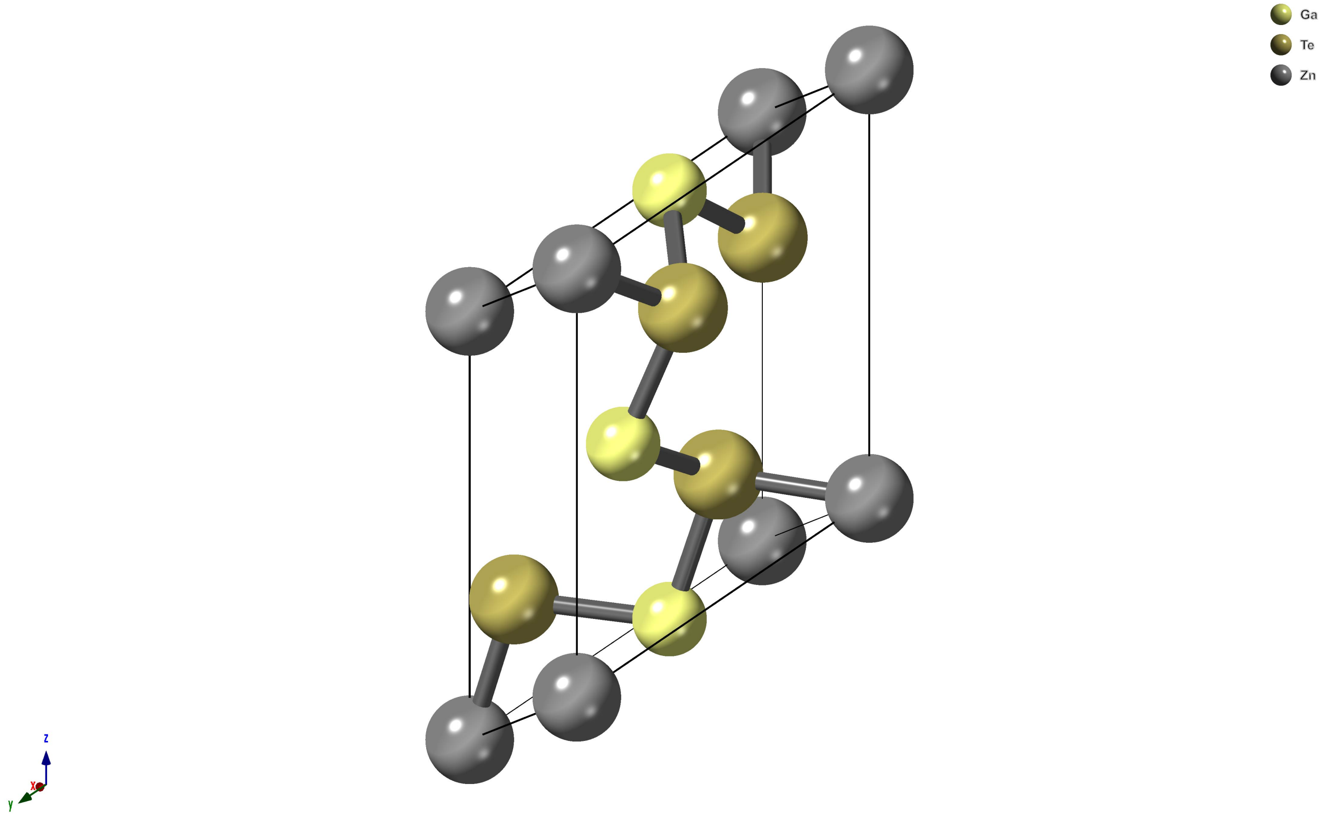

Supplementary Figure 46: Crystal structure of $Zn(GaTe_2)_2$ (mp-15777)

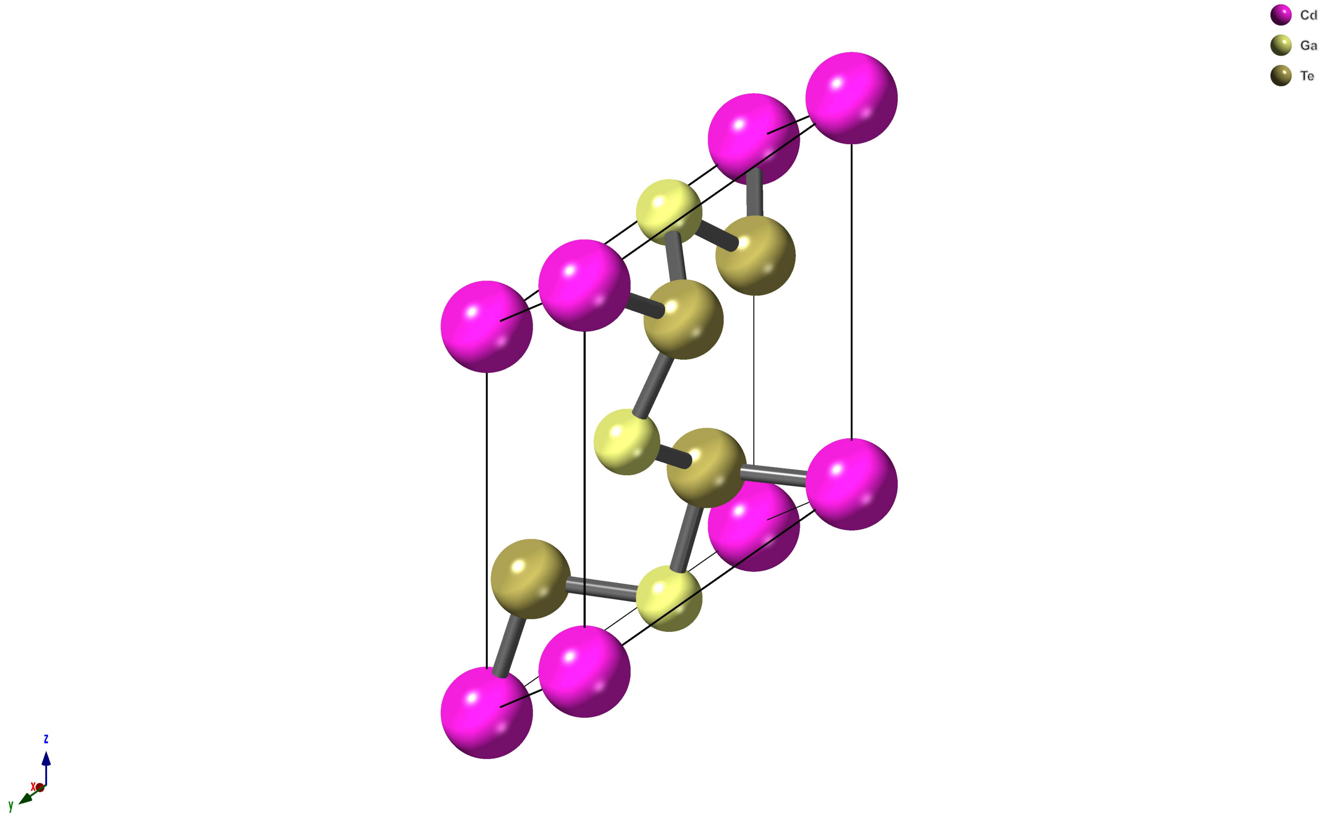

Supplementary Figure 47: Crystal structure of $Cd(GaTe_2)_2$ (mp-13949)

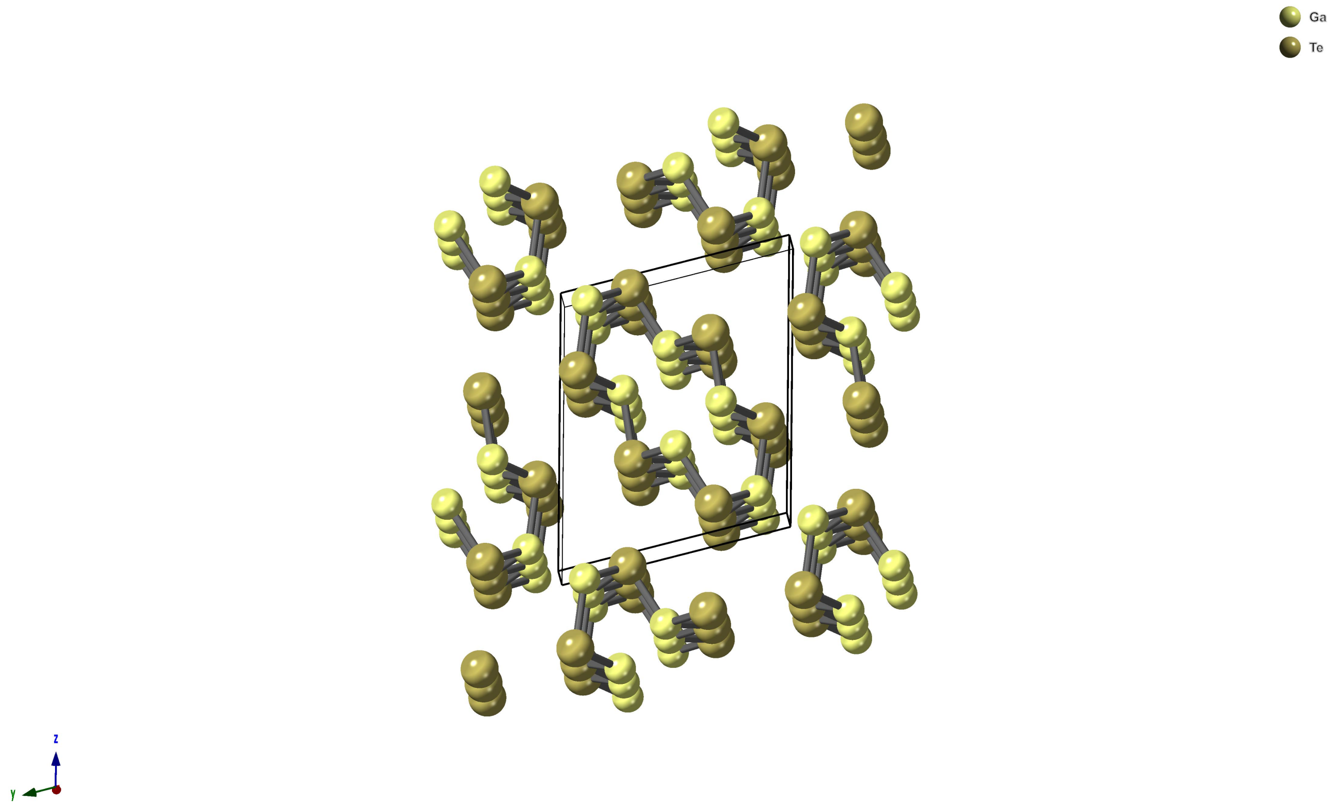

Supplementary Figure 48: Crystal structure of GaTe (mp-542812)

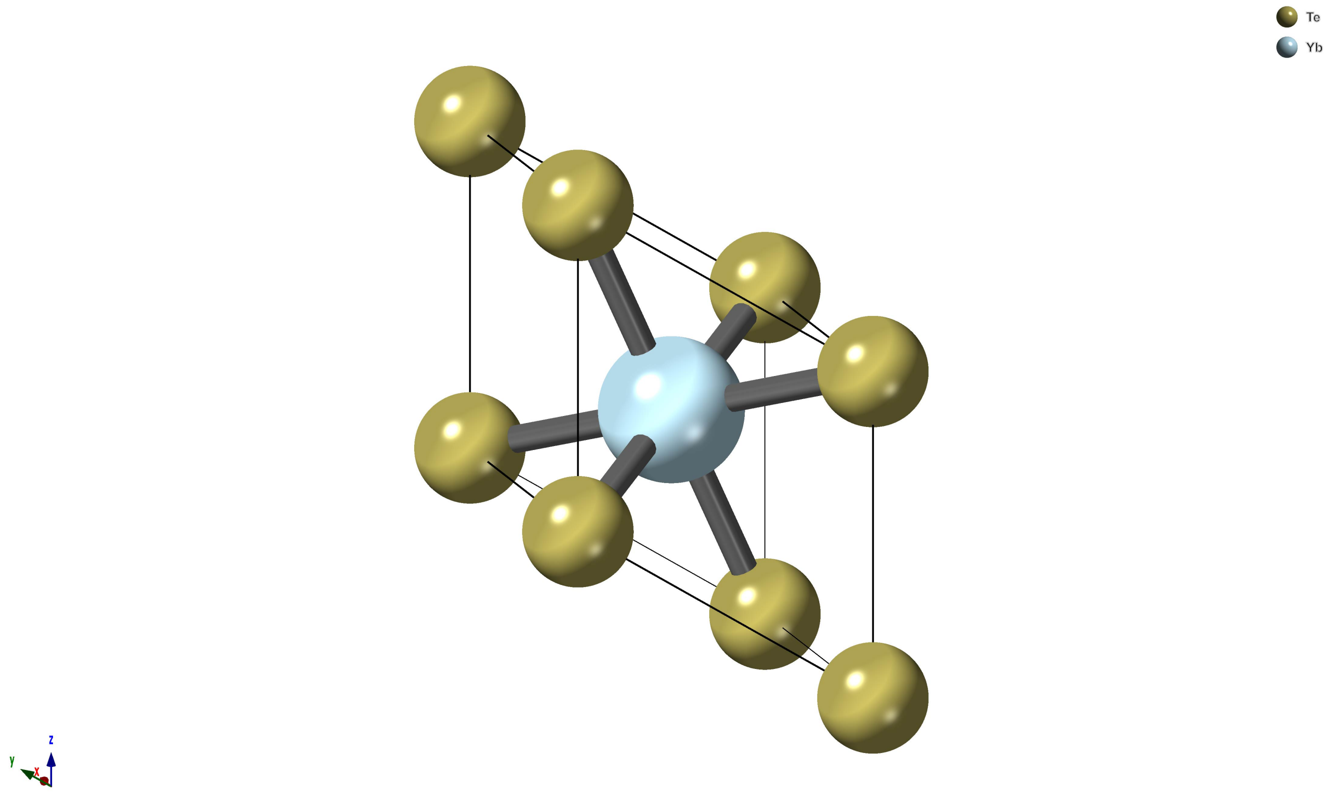

Supplementary Figure 49: Crystal structure of YbTe (mp-1779)

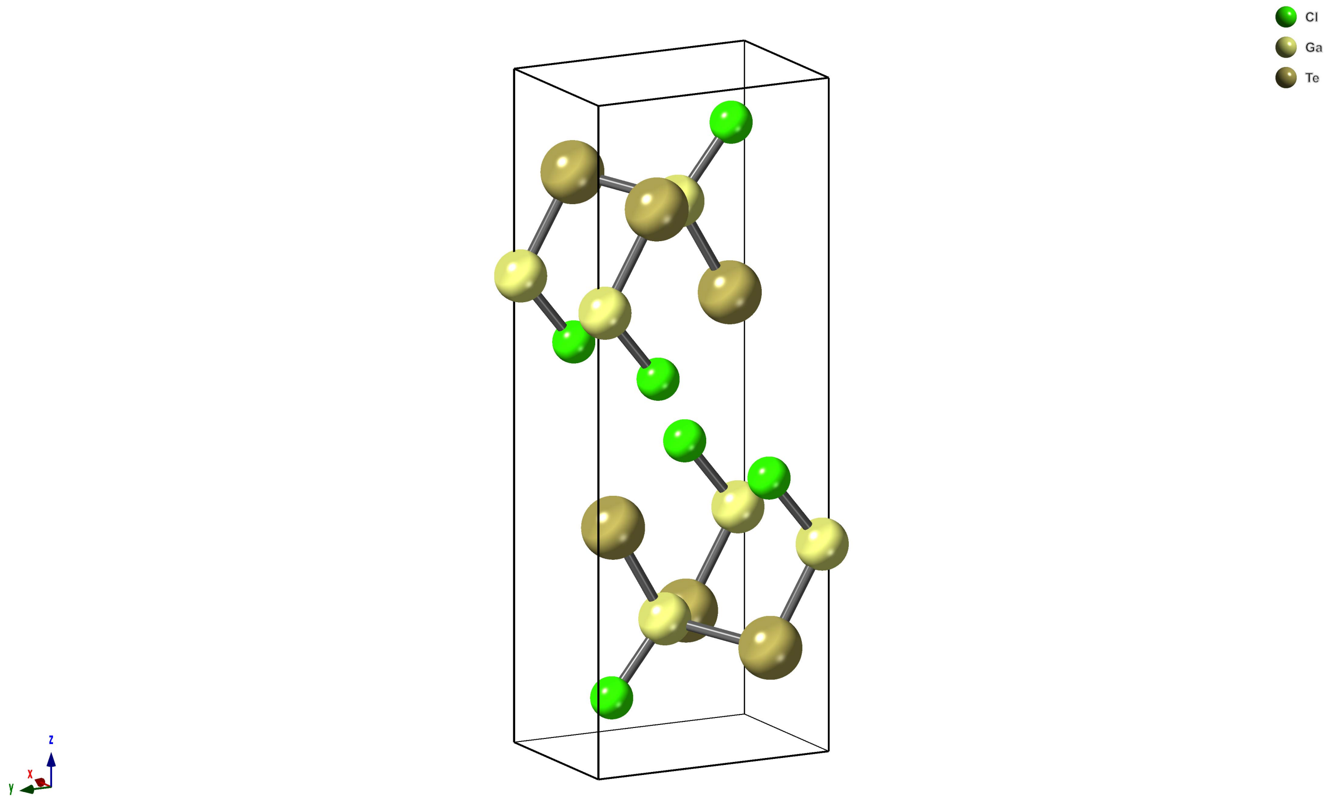

Supplementary Figure 50: Crystal structure of GaTeCl (mp-27449)

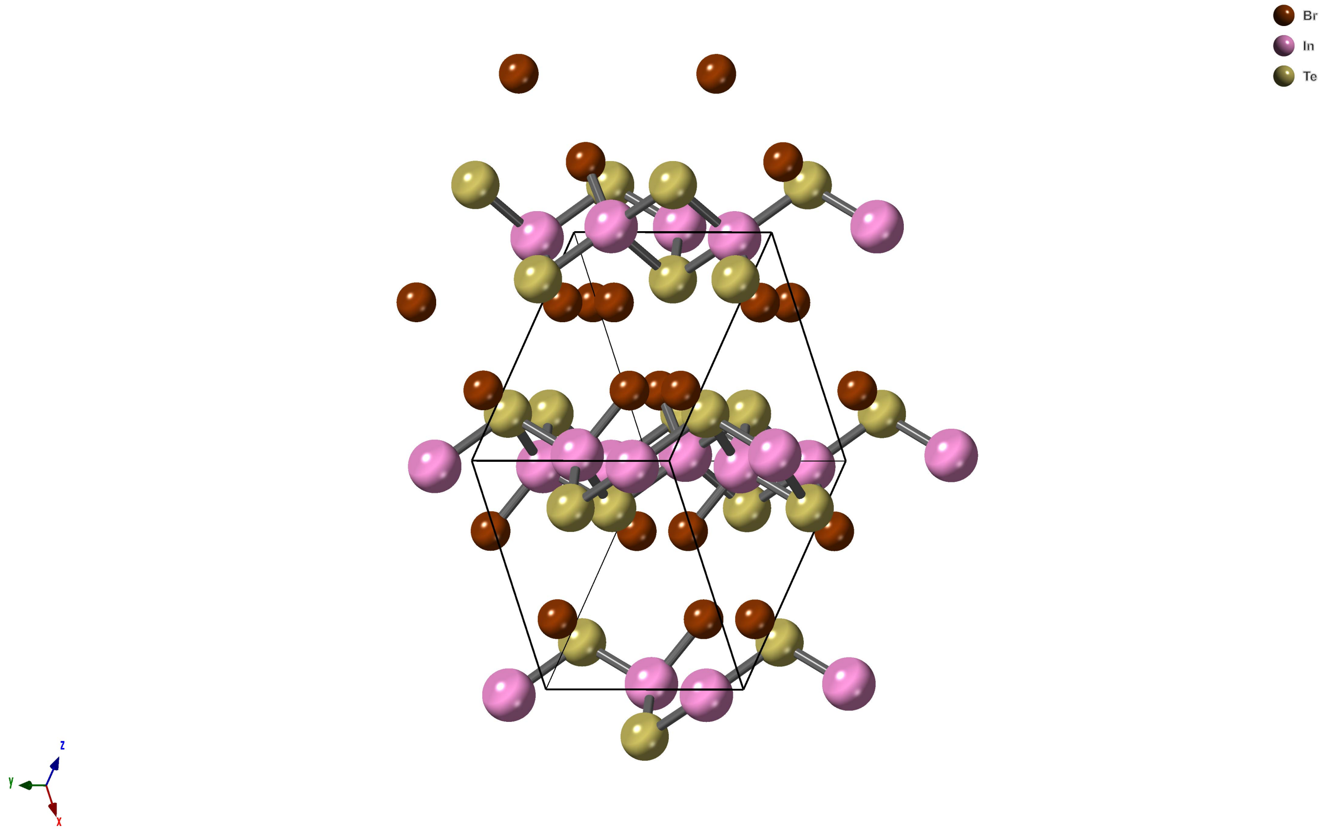

Supplementary Figure 51: Crystal structure of InTeBr (mp-29236)

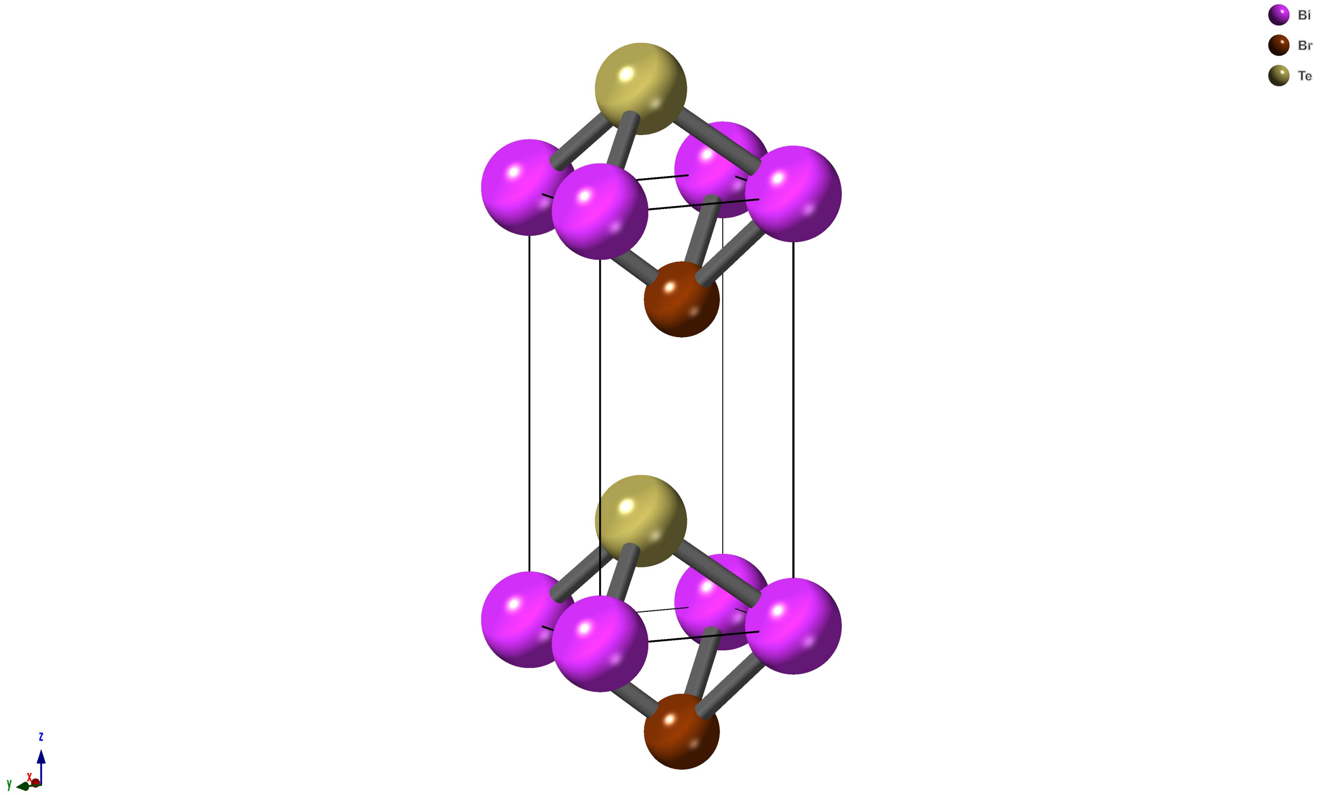

Supplementary Figure 52: Crystal structure of BiTeBr (mp-33723)

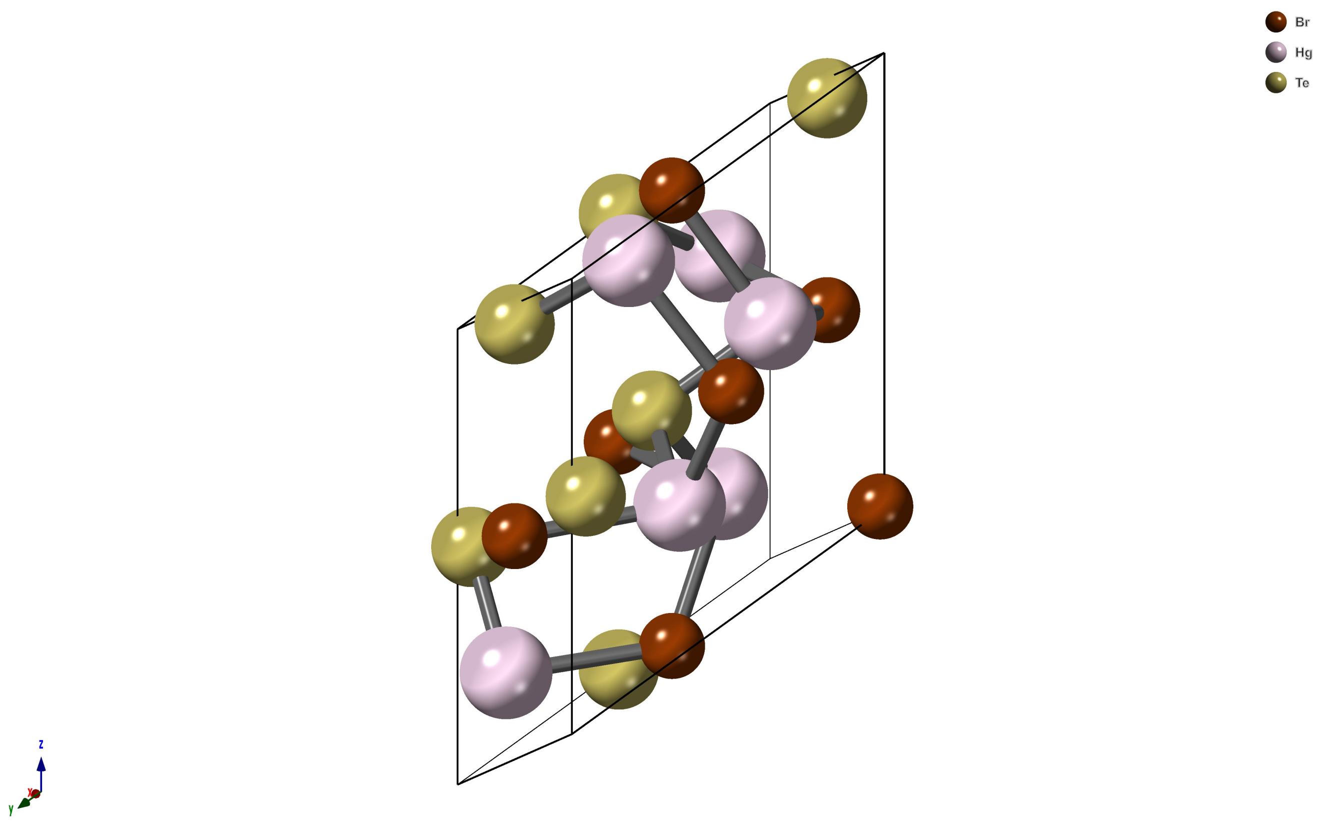

Supplementary Figure 53: Crystal structure of $Hg_3(TeBr)_2$ (mp-27853)

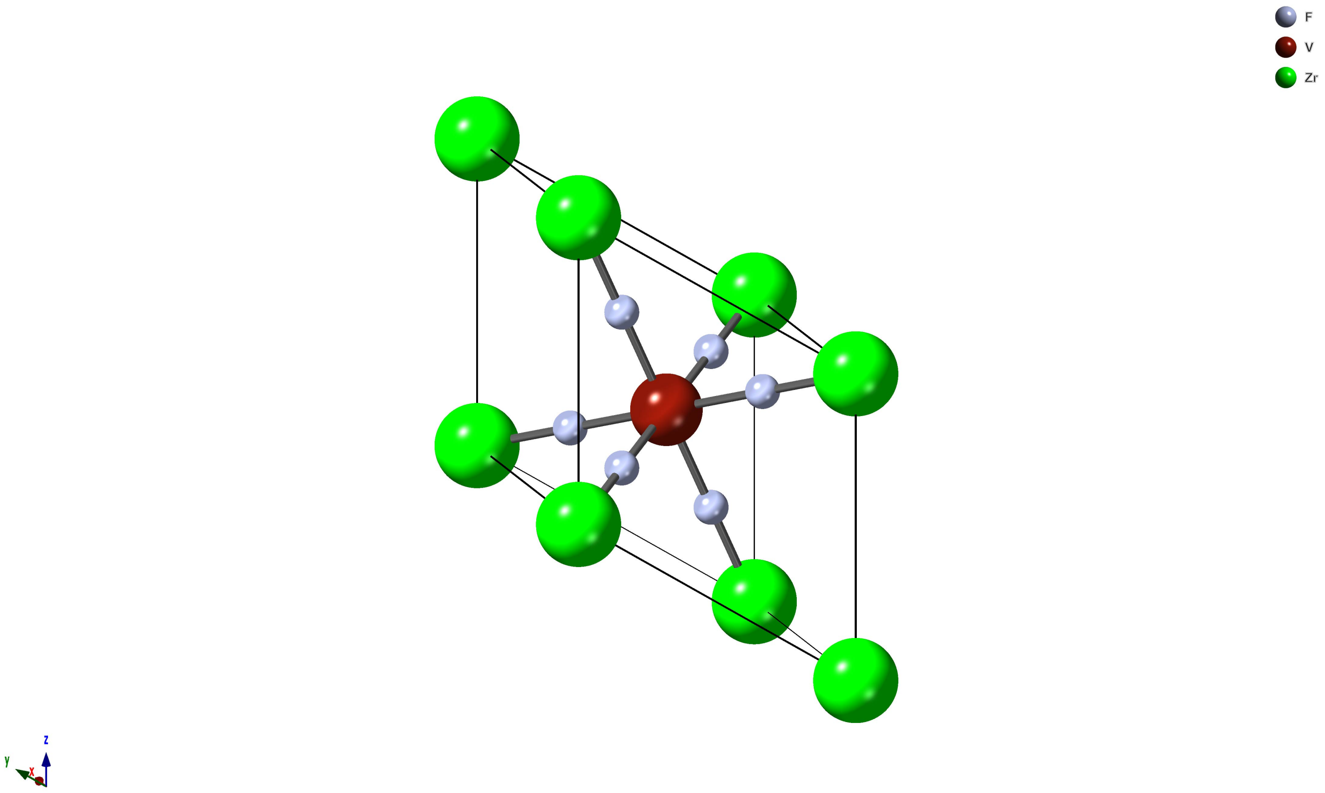

Supplementary Figure 54: Crystal structure of $ZrVF_6$ (mp-557686)

Supplementary Tables

Supplementary Table 1: A list of the properties of materials which pass tier 4 in our computational screening. HSE06 simulations were performed for these 235 water-stable materials. The properties queriable from the MP database as well as the properties specifically computed for this work are listed in the table. Note that this table includes the HSE06 computed bandgaps and band edges of the materials. The material-id is unique to every crystal and can be used to query https://materialsproject.org for properties not listed in the table. The MP material-ids (mp-id), chemical formula (Formula), ICSD-id (if any), and the energy above hull (ΔE^{hull} in eV/atom) is shown for all materials which are pass through tier 4 of the materials screening. Materials which have the Gibbs free energy with respect to Pourbaix stable phases, $\Delta G_{\mathrm{pbx}} < 0.2$ eV/atom at any pH between 0 and 14 and have at least one solid species as decomposition products are noted under the Aq column in the table. The aqueous stability is noted as T (for True) and F (for False) for -0.5 V, -1.0 V and -1.5 V vs RHE, as comma separated symbols. The ΔG_{pbx} in eV/atom, pH and decomposed species for the most negative potential which satisfies the criteria for aqueous stability are also listed. The bandgap computed from PBE or PBE+U ($E^{\mathrm{g}}_{\mathrm{PBE}}$ in eV) and from HSE06 ($E^{\mathrm{g}}_{\mathrm{HSE06}}$ in eV) and if the lowest energy band transition is direct (D) or indirect (I) is also noted in the table. The HSE06 conduction band minima energy in eV is shown for eight surface planes, as applicable.

Formula	mp-id	ICSD-id	ΔE^{hull}	Aq	ΔG_{pbx}	pH	Decomp	$E^{\mathrm{g}}_{\mathrm{PBE}}$	$E^{\mathrm{g}}_{\mathrm{HSE06}}$	CBM (eV)							
										(100)	(010)	(001)	(110)	(011)	(101)	(111)	$(11\bar{1})$
Ti_2FeO_5	mp-31857	37231	0.00	T, F, F	0.062	14	$Fe(s) + TiO_2(s)$	1.6	1.4(I)								
In_2Te_5	mp-582549	501	0.00	T, F, F	0.000	0	$In_2Te_5(s)$	1.0	1.4(I)								
TiCoSb	mp-5967	624919	0.00	F, T, T	0.000	0	TiCoSb(s)	1.1	1.4(I)								
$K_3Sb_2Au_3$	mp-9273	78977	0.00	F, T, T	0.000	14	$K_3Sb_2Au_3(s)$	1.1	1.5(D)								
NaMgSb	mp-7090	41797	0.00	F, F, T	0.000	14	NaMgSb(s)	1.0	1.5(I)								
K_2SbAu	mp-867335	380340	0.00	F, F, T	0.087	14	$Sb_2Au(s) + K_3Sb_2Au_3(s) + K^+$	1.0	1.5(I)								
Ta_2CrO_6	mp-31629	51175	0.00	T, F, F	0.005	7	$Ta_2O_5(s) + TaCrO_4(s)$	1.1	1.6(I)								
Y_2TeO_2	mp-755756	None	0.00	T, T, T	0.144	11	$Y_2O_3(s) + HTe^-$	1.0	1.6(I)								
$Ba(MgAs)_2$	mp-8280	30916	0.00	F, F, T	0.000	9	$Ba(MgAs)_2(s)$	1.1	1.6(I)								
LiBeAs	mp-9562	100004	0.00	F, F, T	0.137	12	$As(s) + Li^+ + BeO_2{}^{2-}$	1.0	1.6(I)								
$BeSiAs_2$	mp-1009087	None	0.00	F, F, T	0.000	6	$BeSiAs_2(s)$	1.0	1.7(D)	-3.38		-3.58			-2.95		
$ZnCdSe_2$	mp-1017534	188387	0.01	T, F, F	0.014	6	CdSe(s) + ZnSe(s)	1.5	1.7(D)	-4.32							
GaTe	mp-542812	153456	0.00	T, T, F	0.070	2	$Ga(s) + H_2Te(aq)$	1.0	1.7(D)				-3.27				
$CsPt_2Se_3$	mp-573316	69440	0.00	T, F, F	0.166	4	$Pt(s) + HSe^- + Cs^+$	1.0	1.7(D)			-1.60					
RbTeAu	mp-9008	71652	0.00	T, F, F	0.148	14	$Au(s) + HTe^- + Rb^+$	1.1	1.7(D)	-4.47	-2.83	-2.08					
$Rb_3Sb_2Au_3$	mp-9274	78978	0.00	F, T, T	0.000	13	$Rb_3Sb_2Au_3(s)$	1.2	1.7(D)			-3.55			-1.22		
K_2CuSb	mp-10381	53298	0.00	F, F, T	0.194	14	$Cu(s) + Sb(s) + K^+$	1.1	1.7(I)				-0.81				
$Sr(InTe_2)_2$	mp-35663	None	0.07	T, F, F	0.169	12	$In_4Te_3(s) + HTe^- + Sr^{2+}$	1.0	1.7(I)	-3.99							
Cs_2SiAs_2	mp-573721	71225	0.00	F, F, T	0.042	14	$SiAs(s) + Cs_3As_7(s) + Cs^+$	1.0	1.7(I)			-0.39		0.09	-1.46		
K_2SiAs_2	mp-6984	40426	0.00	F, F, T	0.046	14	$SiAs_2(s) + K^+$	1.0	1.7(I)			-1.02		-0.23	-2.13		

Continued on next page ...

K_2AgAs	mp-7642	1154	0.00	F, F, T	0.176	14	$Ag(s) + K_3As_{11}(s) + K^+$	1.1	1.7(I)			-1.26	-0.65		-0.63
K_2AgSb	mp-7643	1155	0.00	F, F, T	0.157	14	$Ag(s) + Sb(s) + K^+$	1.2	1.7(I)			-1.54	-1.08		-0.83
$K_2NaAlAs_2$	mp-9069	73280	0.00	F, F, T	0.131	14	$K_3As_{11}(s) + AlAs(s) + Na^+ + K^+$	1.0	1.7(I)			-0.42		-2.03	
$TaCu_3Te_4$	mp-9295	80282	0.00	T, T, F	0.186	2	$Cu(s) + TaO_2{}^+ + H_2Te(aq)$	1.1	1.7(I)						
$Cd(GaTe_2)_2$	mp-13949	25646	0.00	T, T, F	0.127	2	$Ga(s) + CdTe(s) + H_2Te(aq)$	1.0	1.8(D)	-3.13					
$K_3Ag_3As_2$	mp-14206	32016	0.00	F, F, T	0.069	14	$K_3As_{11}(s) + Ag(s) + K^+$	1.3	1.8(D)			-2.29	-1.40	-1.06	
PbSe	mp-22009	74334	0.07	T, F, F	0.160	11	$Pb(s) + HSe^-$	1.3	1.8(D)						
KMgAs	mp-1019089	610753	0.00	F, F, T	0.047	14	$K_3As_{11}(s) + Mg_3As_2(s) + K^+$	1.2	1.8(I)	-1.91		-0.13		-0.86	
K_2CuAs	mp-15684	43936	0.00	F, F, T	0.185	14	$K_3As_7(s) + K(Cu_2As)_2(s) + K^+$	1.1	1.8(I)				-0.23		
$Zn(GaTe_2)_2$	mp-15777	44888	0.00	T, F, F	0.000	0	$Zn(GaTe_2)_2(s)$	1.0	1.8(I)	-3.24					
InSe	mp-21405	32714	0.04	T, F, F	0.105	11	$In(s) + HSe^-$	1.1	1.8(I)						
$RbInTe_2$	mp-22255	75346	0.00	T, F, F	0.067	12	$In_4Te_3(s) + HTe^- + Rb^+$	1.1	1.8(I)	-3.68				-3.12	
AlSb	mp-2624	609290	0.00	F, F, T	0.000	4	AlSb(s)	1.2	1.8(I)	-4.13			-3.15		-3.67
Cd_2AsCl_2	mp-27776	26013	0.00	T, F, F	0.167	2	$Cd_3As_2(s) + Cd^{2+} + Cl^-$	1.1	1.8(I)			-3.57			
BiTeBr	mp-33723	None	0.00	T, F, F	0.095	0	$Bi_8Te_9(s) + Bi_4Te_3(s) + Br^-$	1.2	1.8(I)	-3.52		-4.61		-4.58	
YZnAsO	mp-546011	163780	0.00	T, T, F	0.000	12	YZnAsO(s)	1.2	1.8(I)	-1.98					
CdHgAsBr	mp-569454	240354	0.00	T, F, F	0.191	1	$CdAs_2(s) + Cd^{2+} + Br^- + Hg(aq)$	1.0	1.8(I)		-4.12	-3.36			
KMgSb	mp-7089	41709	0.00	F, F, T	0.000	14	KMgSb(s)	1.3	1.8(I)	-1.85		-0.39		-0.93	
$MgSiAs_2$	mp-1016197	182367	0.00	F, F, T	0.000	10	$SiAs(s) + SiAs_2(s) + Mg_3As_2(s)$	1.3	1.9(D)			-2.90		-3.42	
Ga_2Se_3	mp-1340	37168	0.00	T, F, F	0.113	3	$GaSe(s) + H_2Se(aq)$	1.0	1.9(D)	-3.08					
$MoSe_2$	mp-1634	49800	0.00	T, F, F	0.081	3	$Mo(s) + H_2Se(aq)$	1.4	1.9(D)			-3.81			
$CaTi_4(FeO_4)_3$	mp-24950	79277	0.03	T, F, F	0.189	14	$TiO_2(s) + Fe(s) + CaOH^+$	1.6	1.9(D)						
$K_3Al_2As_3$	mp-28347	60950	0.00	F, F, T	0.116	14	$AlAs(s) + K^+ + Al(OH)_4{}^-$	1.2	1.9(D)		-1.17	-0.38			
CsTeAu	mp-573755	71653	0.00	T, F, F	0.132	14	$Au(s) + HTe^- + Cs^+$	1.3	1.9(D)						
$CaTi_4(FeO_4)_3$	mvc-11639	None	0.03	T, F, F	0.191	14	$Fe(s) + TiO_2(s) + CaOH^+$	1.8	1.9(D)						
$MoSe_2$	mp-1018807	644346	0.00	T, F, F	0.083	3	$Mo(s) + H_2Se(aq)$	1.3	1.9(I)			-3.58			
Gd_2TeO_2	mp-16035	89563	0.00	T, T, T	0.031	11	$Gd_2O_3(s) + HTe^-$	1.1	1.9(I)						
WSe_2	mp-1821	40752	0.00	T, F, F	0.191	3	$W(s) + H_2Se(aq)$	1.4	1.9(I)			-3.52			
La_2MnVO_6	mp-565856	163761	0.00	T, F, F	0.170	14	$La_2O_3(s) + LaVO_3(s) + Mn(OH)_3{}^-$	1.3	1.9(I)						
$MoSe_2$	mp-7581	16948	0.00	T, F, F	0.081	3	$Mo(s) + H_2Se(aq)$	1.4	1.9(I)	-3.56		-4.42	-3.18		
VOF	mp-764689	None	0.04	T, F, F	0.144	0	$V_2O_3(s) + HF(aq)$	1.0	1.9(I)						
$Sr(MgAs)_2$	mp-867194	610831	0.00	F, F, T	0.000	8	$Sr(MgAs)_2(s)$	1.3	1.9(I)	-2.10		-1.94			
$Ca(MgAs)_2$	mp-9564	100041	0.00	F, F, T	0.000	8	$Ca(MgAs)_2(s)$	1.3	1.9(I)	-2.19		-1.95			
ZnTe	mp-571195	80076	0.00	T, T, F	0.003	0	ZnTe(s)	1.1	2(D)	-3.53					
LiMgAs	mp-12558	107954	0.00	F, F, T	0.018	14	$MgAs_4(s) + Mg_3As_2(s) + LiOH(aq)$	1.4	2(I)	-3.40			-2.67		-2.90
$TiFeO_3$	mp-19417	153491	0.00	T, F, F	0.130	14	$Fe(s) + TiO_2(s)$	2.0	2(I)						
LiV_4OF_{11}	mp-777077	None	0.03	T, F, F	0.195	3	$V_2O_3(s) + HF(aq) + Li^+$	1.0	2(I)						
$V_6O_7F_5$	mp-779909	None	0.02	T, F, F	0.183	3	$V_2O_3(s) + HF(aq)$	1.2	2(I)						
Ca_4As_2O	mp-8789	68203	0.00	F, F, T	0.134	14	$CaAs(s) + CaOH^+$	1.2	2(I)			-1.43	-2.05	-2.48	

Continued on next page ...

NaBeAs	mp-9573	100091	0.00	F, F, T	0.130	12	$As(s) + Na^{+} + BeO_2{}^{2-}$	1.3	2(I)			-5.34				
$K_3Cu_3As_2$	mp-14205	32015	0.00	F, F, T	0.101	14	$K(Cu_2As)_2(s) + K_3As_7(s) + K^{+}$	1.3	2.1(D)							
$LiInTe_2$	mp-20782	639906	0.00	T, F, F	0.182	12	$In_4Te_3(s) + Li^{+} + HTe^{-}$	1.4	2.1(D)			-3.73			-3.34	
ZnTe	mp-2176	41984	0.00	T, T, F	0.000	0	ZnTe(s)	1.1	2.1(D)	-4.62			-3.31			
ZnTe	mp-8884	67779	0.01	T, T, F	0.006	0	ZnTe(s)	1.1	2.1(D)	-3.33					-3.81	
GaSe	mp-11342	73388	0.00	T, F, F	0.000	3	GaSe(s)	1.2	2.1(I)						-3.63	
GaSe	mp-1572	71082	0.00	T, F, F	0.000	3	GaSe(s)	1.2	2.1(I)							
YbTe	mp-1779	653185	0.00	T, T, T	0.027	0	$YbO(s) + H_2Te(aq)$	1.5	2.1(I)	-2.66			-2.45			
SiAs	mp-1863	43227	0.00	F, F, T	0.000	0	SiAs(s)	1.5	2.1(I)	-2.56		-2.91				
$TiFeO_3$	mp-19270	246515	0.02	T, F, F	0.187	14	$Fe(s) + TiO_2(s)$	2.0	2.1(I)							
GaSe	mp-1943	63122	0.00	T, F, F	0.000	3	GaSe(s)	1.2	2.1(I)			-3.93				
AlAs	mp-2172	606009	0.00	F, F, T	0.000	0	AlAs(s)	1.5	2.1(I)				-3.45			
BiTeCl	mp-28944	79362	0.00	T, F, F	0.126	0	$Bi_4Te_3(s) + Bi_8Te_9(s) + Cl^{-}$	1.5	2.1(I)							
$SrLiNiF_6$	mp-559663	32733	0.00	T, F, F	0.195	0	$Ni(s) + HF(aq) + Li^{+} + Sr^{2+}$	2.3	2.1(I)							
GaSe	mp-568263	2002	0.00	T, F, F	0.001	3	GaSe(s)	1.2	2.1(I)							
VOF	mp-764830	None	0.00	T, F, F	0.089	0	$V_2O_3(s) + HF(aq)$	1.1	2.1(I)							
RbCaSb	mp-9846	409178	0.00	F, F, T	0.157	14	$CaSb_2(s) + Ca_{11}Sb_{10}(s) + Rb^{+}$	1.5	2.1(I)	-1.42		-0.43			-0.86	
LaCuTeO	mp-546790	416522	0.01	T, F, F	0.059	12	$Cu(s) + La_2TeO_2(s) + HTe^{-}$	1.2	2.2(D)							
$VCrO_3$	mp-769640	None	0.01	T, F, F	0.019	9	$V_2O_3(s) + Cr_2O_3(s)$	1.3	2.2(D)							
VOF	mp-764900	None	0.00	T, F, F	0.088	0	$V_2O_3(s) + HF(aq)$	1.4	2.2(I)							
$VCrO_3$	mp-768065	None	0.01	T, F, F	0.020	9	$Cr_2O_3(s) + V_2O_3(s)$	1.3	2.2(I)							
$VCrO_3$	mp-770843	None	0.01	T, F, F	0.020	9	$V_2O_3(s) + Cr_2O_3(s)$	1.2	2.2(I)							
NaCaAs	mp-961685	None	0.00	F, F, T	0.175	14	$CaAs(s) + Na^{+}$	1.6	2.2(I)				-1.66			-3.53
ZnSe	mp-1190	652224	0.00	T, F, F	0.000	6	ZnSe(s)	1.2	2.3(D)				-3.78			
$Zn(GaSe_2)_2$	mp-15776	168594	0.00	T, F, F	0.069	4	$Ga_2O_3(s) + HSe^{-} + Zn^{2+}$	1.4	2.3(D)							-3.68
$Cd(GaSe_2)_2$	mp-3772	93761	0.00	T, F, F	0.055	3	$GaSe(s) + CdSe(s) + H_2Se(aq)$	1.3	2.3(D)							
ZnSe	mp-380	652217	0.00	T, F, F	0.005	6	ZnSe(s)	1.2	2.3(D)							
$CsLaZnTe_3$	mp-510460	170184	0.00	T, F, F	0.208	12	$La_2TeO_2(s) + ZnTe(s) + HTe^{-} + Cs^{+}$	1.9	2.3(D)		-1.20					
$Cs_2Cd_3Te_4$	mp-567386	90369	0.00	T, T, F	0.162	3	$CdTe(s) + HTe^{-} + Cs^{+}$	1.6	2.3(D)			-2.78				
YVO_3	mvc-14992	None	0.00	T, F, F	0.001	11	$YVO_3(s)$	1.5	2.3(D)							
Ta_2FeO_6	mp-31755	401264	0.00	T, F, F	0.000	7	$Ta_2FeO_6(s)$	2.4	2.3(I)							
Pr_2PbSe_4	mp-675146	None	0.02	T, F, F	0.165	7	$Pb(s) + Pr(OH)_2{}^{+} + HSe^{-}$	1.7	2.3(I)							
MgV_2O_4	mvc-6900	None	0.07	T, F, F	0.170	14	$V_2O_3(s) + MgOH^{+}$	1.4	2.3(I)							
$CsLaCdTe_3$	mp-12491	173316	0.00	T, F, F	0.199	12	$CdTe(s) + La_2TeO_2(s) + HTe^{-} + Cs^{+}$	1.7	2.4(D)				-2.80			
BaAgTeF	mp-16742	419382	0.00	T, F, F	0.115	11	$Ag(s) + Ba^{2+} + F^{-} + HTe^{-}$	1.5	2.4(D)						-1.93	
$TmVO_3$	mp-778918	None	0.00	T, F, F	0.000	10	$TmVO_3(s)$	1.6	2.4(D)							
V_2ZnO_4	mvc-15001	None	0.00	T, F, F	0.001	8	$V_2ZnO_4(s)$	1.6	2.4(D)							
$LiTi_2V_3O_{10}$	mp-768019	None	0.07	T, F, F	0.206	3	$V_2O_3(s) + Li^{+} + Ti^{2+}$	1.4	2.4(I)							
VOF	mp-768032	None	0.04	T, F, F	0.139	0	$V_2O_3(s) + HF(aq)$	1.4	2.4(I)							

Continued on next page ...

$LiV_4O_5F_7$	mp-849984	None	0.05	T, F, F	0.138	3	$V_2O_3(s) + HF(aq) + Li^+$	1.4	2.4(I)					
AlAs	mp-8881	67771	0.01	F, F, T	0.006	0	AlAs(s)	1.7	2.4(I)					-3.40
LiCaAs	mp-961684	None	0.00	F, F, T	0.167	14	CaAs(s) + LiOH(aq)	1.8	2.4(I)			-1.69		-2.13
$Hg_3(TeBr)_2$	mp-27853	27402	0.00	T, F, F	0.201	0	$HgTe(s) + Hg(aq) + Br^-$	1.7	2.5(D)				-3.36	
Ga_2TeSe_2	mp-28423	64617	0.00	T, F, F	0.095	3	$GaTe(s) + GaSe(s) + H_2Se(aq)$	1.6	2.5(D)				-3.26	
MgV_2O_4	mvc-11622	None	0.00	T, F, F	0.001	14	$V_2O_3(s) + MgOH^+$	1.5	2.5(D)					
MgV_2O_4	mvc-9996	None	0.05	T, F, F	0.119	14	$V_2O_3(s) + MgOH^+$	1.3	2.5(D)					
V_2ZnO_4	mp-18879	55442	0.00	T, F, F	0.000	8	$V_2ZnO_4(s)$	1.7	2.5(I)					
VClO	mp-25118	27011	0.00	T, F, F	0.204	0	$V_2O_3(s) + Cl^-$	1.6	2.5(I)		-4.71			
$Ce_5Y_2O_{13}$	mp-753355	None	0.04	T, F, F	0.125	13	$Y_2O_3(s) + CeO_2(s)$	1.3	2.5(I)					
$ScVO_3$	mp-769785	None	0.05	T, F, F	0.126	5	$V_2O_3(s) + Sc_2O_3(s)$	1.5	2.5(I)					
VOF	mp-778746	None	0.00	T, F, F	0.085	0	$V_2O_3(s) + HF(aq)$	1.7	2.5(I)					
InTeBr	mp-29236	100705	0.00	T, F, F	0.094	0	$In_7Te_{10}(s) + In^+ + Br^-$	1.6	2.6(D)					
VF_3	mp-765007	None	0.00	T, F, F	0.144	0	$V_2O_3(s) + HF(aq)$	1.7	2.6(D)					
$TiMnO_3$	mp-565904	158732	0.06	T, F, F	0.140	12	$TiMnO_3(s)$	1.5	2.6(I)					
$Ce_5Gd_2O_{13}$	mp-753088	None	0.04	T, F, F	0.105	13	$CeO_2(s) + Gd_2O_3(s)$	1.2	2.6(I)					
$LiV_6O_7F_5$	mp-765863	None	0.05	T, F, F	0.198	3	$V_2O_3(s) + HF(aq) + Li^+$	1.5	2.6(I)					
$VCrO_3$	mp-770779	None	0.01	T, F, F	0.015	9	$V_2O_3(s) + Cr_2O_3(s)$	1.6	2.6(I)					
$CaLaTaCrO_6$	mvc-10008	None	0.02	T, F, F	0.171	14	$LaTaO_4(s) + Cr_2O_3(s) + CaOH^+$	2.0	2.6(I)					
VOF	mp-768037	None	0.02	T, F, F	0.113	0	$V_2O_3(s) + HF(aq)$	1.6	2.7(D)		-4.12			
$InSnCl_3$	mp-998560	None	0.02	T, F, F	0.106	11	$Sn(s) + Cl^- + In^+$	2.1	2.7(D)					
VOF	mp-764309	None	0.02	T, F, F	0.117	0	$V_2O_3(s) + HF(aq)$	1.7	2.7(I)	-3.03				
$TaVO_4$	mp-772246	None	0.00	T, F, F	0.000	4	$TaVO_4(s)$	1.8	2.7(I)					
$AcCrO_3$	mp-866647	None	0.00	T, F, F	0.000	5	$AcCrO_3(s)$	1.8	2.7(I)					
VF_3	mp-559931	30624	0.00	T, F, F	0.142	0	$V_2O_3(s) + HF(aq)$	1.5	2.8(D)					
La_2TeO_2	mp-4547	27004	0.00	T, T, T	0.000	12	$La_2TeO_2(s)$	2.0	2.8(I)				0.19	
$CsGaSe_3$	mp-510283	98670	0.00	T, F, F	0.143	3	$GaSe(s) + Cs^+$	1.9	2.8(I)	-1.77	-2.58			
Gd_2SeO_2	mp-13973	25808	0.00	T, T, T	0.195	11	$Gd_2O_3(s) + HSe^-$	1.3	2.9(I)					
$Ce_3Y_4O_{12}$	mp-755046	None	0.07	T, F, F	0.186	13	$Y_2O_3(s) + CeO_2(s)$	1.5	2.9(I)					
$LiTiCrO_4$	mp-771738	None	0.00	T, F, F	0.107	14	$TiO_2(s) + Cr_2O_3(s) + LiOH(aq)$	1.7	2.9(I)					
La_2MgVO_6	mvc-11733	None	0.00	T, F, F	0.146	14	$LaVO_3(s) + La_2O_3(s) + MgOH^+$	1.7	2.9(I)					
GaTeCl	mp-27449	15582	0.00	T, F, F	0.119	0	$GaTe(s) + Cl^-$	2.2	3(D)	-2.66	-3.91			
VOF	mp-764286	None	0.03	T, F, F	0.134	0	$V_2O_3(s) + HF(aq)$	2.0	3(D)					
$TiMnO_3$	mp-19376	65359	0.03	T, F, F	0.084	12	$TiMnO_3(s)$	1.9	3(I)					
VF_3	mp-766116	None	0.05	T, F, F	0.191	0	$V_2O_3(s) + HF(aq)$	1.7	3(I)					
Ta_2MnO_6	mp-767031	None	0.01	T, F, F	0.024	8	$Ta_2MnO_6(s)$	2.2	3(I)					
VOF	mp-767190	None	0.05	T, F, F	0.164	0	$V_2O_3(s) + HF(aq)$	1.5	3(I)					
VF_2	mp-555934	32552	0.00	T, T, F	0.175	3	V(s) + HF(aq)	2.3	3.1(D)					
$CsYZnSe_3$	mp-574620	280847	0.00	T, F, F	0.164	10	$Zn(s) + HSe^- + Y(OH)^{2+} + Cs^+$	2.1	3.1(D)	-0.37		-2.44		

Continued on next page ...

LiV_2F_7	mp-776622	None	0.01	T, F, F	0.196	3	$V_2O_3(s) + HF(aq) + Li^+$	2.1	3.1(D)								
$NaTaO_3$	mp-4170	88378	0.01	T, T, F	0.145	14	$NaTa_4O_8(s) + Na^+$	2.3	3.1(I)	-3.14							
Ta_2SnO_6	mp-556489	54078	0.00	T, F, F	0.073	14	$Sn(s) + Ta_2O_5(s)$	2.3	3.1(I)			-3.95					
KVF_3	mp-557257	28145	0.00	T, F, F	0.200	3	$V_2O_3(s) + HF(aq) + K^+$	2.1	3.1(I)								
$NaZr_2VF_{11}$	mp-558441	78868	0.00	T, F, F	0.189	3	$V_2O_3(s) + ZrO^{2+} + HF(aq) + Na^+$	2.2	3.1(I)								
VOF	mp-767367	None	0.04	T, F, F	0.143	0	$V_2O_3(s) + HF(aq)$	2.0	3.1(I)								
TiO_2	mp-2657	202240	0.04	T, F, F	0.110	4	$TiO_2(s)$	1.8	3.2(D)						-4.22		
$LiVF_4$	mp-782719	None	0.06	T, F, F	0.076	0	$V_2O_3(s) + HF(aq) + Li^+$	2.1	3.2(D)		-4.40						
$CeThO_4$	mp-36734	None	0.01	T, T, F	0.117	14	$ThO_2(s) + Ce^{3+}$	1.7	3.2(I)	-2.35					-3.43		
$SrTiO_3$	mp-5229	80872	0.00	T, T, F	0.160	14	$Ti_2O_3(s) + SrOH^+$	2.1	3.2(I)	-2.99							
La_2UO_6	mp-760513	None	0.00	T, T, F	0.200	14	$La_6UO_{12}(s) + UO_2(s)$	1.6	3.2(I)		-1.42						
$NbCrO_4$	mp-765437	None	0.01	T, F, F	0.161	9	$Cr_2O_3(s) + NbO_3^-$	2.0	3.2(I)								
$LiVF_4$	mp-780742	None	0.00	T, F, F	0.208	3	$V_2O_3(s) + HF(aq) + Li^+$	2.1	3.2(I)								
$SrTiO_3$	mp-4651	182248	0.00	T, T, F	0.158	14	$Ti_2O_3(s) + SrOH^+$	1.8	3.3(I)						-1.89		
$ZrVF_6$	mp-557686	73354	0.00	T, T, F	0.185	3	$V(s) + ZrO^{2+} + HF(aq)$	2.4	3.3(I)	-2.63			-2.52			-3.47	
LiV_2F_7	mp-776623	None	0.07	T, T, F	0.182	0	$V(s) + HF(aq) + Li^+$	2.1	3.3(I)								
TiO_2	mvc-6590	None	0.04	T, F, F	0.115	4	$TiO_2(s)$	1.9	3.3(I)		-4.34						
$LaCrO_3$	mp-19281	91270	0.00	T, F, F	0.000	12	$LaCrO_3(s)$	2.2	3.4(D)								
$Ce_4Y_2O_{11}$	mp-760491	None	0.06	T, F, F	0.170	13	$Y_2O_3(s) + CeO_2(s)$	1.8	3.4(D)								
$LaCrO_3$	mp-19357	167590	0.01	T, F, F	0.023	12	$LaCrO_3(s)$	2.3	3.4(I)								
$KTaO_3$	mp-3614	39905	0.00	T, T, F	0.179	14	$Ta(s) + K^+$	2.1	3.4(I)	-2.13							
$CsSr_2Ta_3O_{10}$	mp-7181	93677	0.00	T, T, F	0.156	14	$SrTa_2O_6(s) + SrOH^+ + Cs^+$	2.0	3.4(I)								
$CeTh_3O_8$	mp-753823	None	0.01	T, T, T	0.191	14	$ThO_2(s) + Ce^{3+}$	1.8	3.4(I)			-1.90					
Cr_2O_3	mp-776999	None	0.06	T, F, F	0.149	9	$Cr_2O_3(s)$	2.1	3.4(I)								
$CaVF_5$	mvc-14225	None	0.00	T, F, F	0.168	3	$V_2O_3(s) + HF(aq) + Ca^{2+}$	2.3	3.4(I)								
$CeZrO_4$	mp-1019596	None	0.04	T, F, F	0.134	13	$ZrO_2(s) + CeO_2(s)$	1.8	3.5(I)								
$TiMnO_3$	mp-19082	247553	0.00	T, F, F	0.000	12	$TiMnO_3(s)$	2.3	3.5(I)								
CeO_2	mp-20194	164225	0.00	T, T, F	0.000	13	$CeO_2(s)$	1.9	3.5(I)				-2.00			-3.55	
TiO_2	mp-34688	None	0.01	T, T, F	0.169	14	$Ti_2O_3(s)$	2.0	3.5(I)								-3.69
TiO_2	mp-390	202242	0.01	T, T, F	0.145	14	$Ti_2O_3(s)$	2.1	3.5(I)			-3.49					
$CaLaTiCrO_6$	mp-39159	None	0.00	T, F, F	0.103	14	$La_2Ti_2O_7(s) + Cr_2O_3(s) + CaOH^+$	2.4	3.5(I)								
$RbVF_3$	mp-556424	28146	0.00	T, F, F	0.195	3	$V_2O_3(s) + HF(aq) + Rb^+$	2.4	3.5(I)								
La_2SeO_2	mp-7233	25804	0.00	T, T, T	0.100	13	$La_2O_3(s) + HSe^-$	2.4	3.5(I)			-1.09					
$CeHfO_4$	mp-752669	None	0.06	T, F, F	0.170	12	$CeO_2(s) + HfO_2(s)$	1.8	3.5(I)								
$CeTh_2O_6$	mp-752798	None	0.01	T, T, F	0.083	14	$ThO_2(s) + Ce^{3+}$	1.8	3.5(I)			-1.62					
$Li_2La_2Ti_3O_{10}$	mp-9406	82907	0.02	T, T, F	0.186	14	$Ti_2O_3(s) + La_2Ti_2O_7(s) + LiOH(aq)$	2.1	3.5(I)								
$CaTiO_3$	mp-556003	50364	0.01	T, T, F	0.178	14	$Ti_2O_3(s) + CaOH^+$	2.5	3.6(D)								
La_6UO_{12}	mp-19992	202585	0.00	T, T, T	0.075	14	$La_2O_3(s) + U^{3+}$	2.0	3.6(I)								
$CaUO_4$	mp-3960	23195	0.00	T, F, F	0.144	14	$UO_2(s) + CaOH^+$	2.2	3.6(I)								

Continued on next page ...

$MnCr_2O_4$	mp-541022	167400	0.00	T, F, F	0.000	9	$MnCr_2O_4(s)$	2.4	3.6(I)
$SrLi_2Ta_2O_7$	mp-6259	88464	0.00	T, T, F	0.176	14	$SrTa_2O_6(s) + LiOH(aq)$	2.2	3.6(I)
Ta_3O_7F	mp-753747	None	0.00	T, T, F	0.187	4	$Ta(s) + F^-$	2.2	3.6(I)
$TaCrO_4$	mp-766842	None	0.00	T, F, F	0.000	7	$TaCrO_4(s)$	2.3	3.6(I)
Cr_2O_3	mp-776526	None	0.04	T, F, F	0.095	9	$Cr_2O_3(s)$	2.3	3.7(D)
Y_6UO_{12}	mp-27698	23966	0.00	T, T, T	0.205	14	$Y_2O_3(s) + U^{3+}$	1.9	3.7(I)
$CaTiO_3$	mp-3442	162919	0.02	T, T, F	0.196	14	$Ti_2O_3(s) + CaOH^+$	2.2	3.7(I)
TiO_2	mp-430	154035	0.07	T, F, F	0.196	4	$TiO_2(s)$	2.2	3.7(I)
$NaTaO_3$	mp-4675	88377	0.00	T, T, F	0.120	14	$NaTa_4O_8(s) + Na^+$	2.3	3.7(I)
$NaTaO_3$	mp-4699	88376	0.00	T, T, F	0.112	14	$NaTa_4O_8(s) + Na^+$	2.4	3.7(I)
$RbLaTa_2O_7$	mp-541600	86208	0.02	T, T, F	0.183	14	$LaTaO_4(s) + Ta(s) + Rb^+$	2.3	3.7(I)
$La_2Th_5O_{13}$	mp-756142	None	0.03	T, T, T	0.090	14	$La_2O_3(s) + ThO_2(s)$	2.4	3.7(I)
TiO_2	mp-766454	None	0.06	T, F, F	0.176	4	$TiO_2(s)$	2.3	3.7(I)
$ScCrO_3$	mp-18961	85141	0.03	T, F, F	0.086	9	$Cr_2O_3(s) + Sc_2O_3(s)$	2.5	3.8(D)
$MnSnF_6$	mp-558761	25010	0.00	T, F, F	0.084	1	$Sn(s) + HF(aq) + Mn^{2+}$	2.4	3.8(D)
$YbTiO_3$	mp-754225	None	0.00	T, T, T	0.132	14	$Ti_2O(s) + YbO(s)$	2.3	3.8(D)
Ga_2O_3	mp-886	34243	0.00	T, F, F	0.000	5	$Ga_2O_3(s)$	2.0	3.8(D)
$CaTiO_3$	mvc-11484	None	0.00	T, T, F	0.150	14	$Ti_2O_3(s) + CaOH^+$	2.5	3.8(D)
Cr_2CdO_4	mp-19262	37428	0.00	T, F, F	0.169	14	$Cd(s) + Cr_2O_3(s)$	2.5	3.8(I)
Cr_2O_3	mp-19399	201102	0.00	T, F, F	0.000	9	$Cr_2O_3(s)$	2.4	3.8(I)
$K_4CaU_3O_{12}$	mp-16137	91783	0.00	T, F, F	0.123	14	$UO_2(s) + K^+ + CaOH^+$	2.1	3.9(I)
$NaCaTaTiO_6$	mp-39712	None	0.01	T, T, F	0.162	14	$Ti_2O_3(s) + CaTa_2O_6(s) + Na^+ + CaOH^+$	2.4	3.9(I)
$TiGa_2O_5$	mp-752733	None	0.08	T, F, F	0.205	5	$Ga_2O_3(s) + TiO_2(s)$	2.3	3.9(I)
$Ti(GaO_2)_4$	mp-553991	155638	0.00	T, F, F	0.007	5	$TiO_2(s) + Ga_2O_3(s)$	2.4	4(D)
$Mg(GaO_2)_2$	mp-753846	None	0.02	T, F, F	0.135	11	$Ga_2O_3(s) + Mg^{2+}$	2.4	4(D)
TiO_2	mp-9173	75179	0.06	T, F, F	0.178	4	$TiO_2(s)$	2.5	4(I)
$Zn(GaO_2)_2$	mp-5794	81106	0.00	T, F, F	0.000	7	$Zn(GaO_2)_2(s)$	2.3	4.1(I)
$Mg(GaO_2)_2$	mp-38951	None	0.00	T, F, F	0.081	11	$Ga_2O_3(s) + Mg^{2+}$	2.4	4.2(D)
Ga_2O_3	mp-1243	166199	0.03	T, F, F	0.074	5	$Ga_2O_3(s)$	2.4	4.2(I)
Ta_2O_5	mp-10390	280396	0.01	T, F, F	0.026	7	$Ta_2O_5(s)$	1.2	
$CsNdZnTe_3$	mp-12342	170186	0.00	T, F, F	0.197	11	$Nd_2TeO_2(s) + ZnTe(s) + HTe^- + Cs^+$	1.4	
Nd_2SeO_2	mp-13971	25806	0.00	T, T, T	0.118	12	$Nd_2O_3(s) + HSe^-$	2.1	
Sm_2SeO_2	mp-13972	25807	0.00	T, T, F	0.178	11	$Sm_2O_3(s) + HSe^-$	2.1	
Sm_2TeO_2	mp-16033	89561	0.00	T, T, T	0.000	11	$Sm_2TeO_2(s)$	1.4	
Tb_2TeO_2	mp-16036	89564	0.00	T, T, T	0.066	9	$Tb_2O_3(s) + HTe^-$	1.2	
Dy_2TeO_2	mp-16037	89565	0.00	T, T, T	0.121	10	$Dy_2O_3(s) + HTe^-$	1.1	
$YCrO_3$	mp-18725	109352	0.00	T, F, F	0.000	11	$YCrO_3(s)$	2.5	
Sm_2TiMnO_6	mp-18794	90453	0.02	T, F, F	0.111	12	$Sm_2TiO_5(s) + Mn(OH)_3^-$	1.9	
YVO_3	mp-18883	246953	0.00	T, F, F	0.000	11	$YVO_3(s)$	1.6	

Continued on next page ...

$HoVO_3$	mp-19024	10018	0.00	T, F, F	0.000	10	$HoVO_3(s)$	1.6
$ErVO_3$	mp-19205	40393	0.00	T, F, F	0.000	10	$ErVO_3(s)$	1.6
$NdCrO_3$	mp-19269	156320	0.00	T, F, F	0.000	11	$NdCrO_3(s)$	2.3
$DyCrO_3$	mp-19381	28248	0.00	T, F, F	0.000	10	$DyCrO_3(s)$	2.5
$GdCrO_3$	mp-19598	9985	0.00	T, F, F	0.000	11	$GdCrO_3(s)$	2.4
Lu_6UO_{12}	mp-20402	23967	0.00	T, T, F	0.085	14	$UO_2(s) + Lu_2O_3(s)$	1.9
$DyVO_3$	mp-25144	40392	0.00	T, F, F	0.000	10	$DyVO_3(s)$	1.5
Nd_2TeO_2	mp-5459	89560	0.00	T, T, T	0.000	11	$Nd_2TeO_2(s)$	1.6
$NdVO_3$	mp-556421	63522	0.00	T, F, F	0.000	11	$NdVO_3(s)$	1.0
$TbVO_3$	mp-565331	155481	0.00	T, F, F	0.000	9	$TbVO_3(s)$	1.5
$Ce_3Dy_2O_9$	mp-675192	None	0.07	T, F, F	0.189	13	$Dy_2O_3(s) + CeO_2(s)$	1.9
$Ce_3Nd_2O_9$	mp-676077	None	0.06	T, F, F	0.154	13	$Nd_2O_3(s) + CeO_2(s)$	2.0
$CsErZnSe_3$	mp-7155	280844	0.00	T, F, F	0.167	9	$Zn(s) + HSe^- + Er^{3+} + Cs^+$	2.5
$Ce_5Dy_2O_{13}$	mp-753763	None	0.04	T, F, F	0.126	13	$Dy_2O_3(s) + CeO_2(s)$	1.3
$Ce_5Sm_2O_{13}$	mp-753792	None	0.04	T, F, F	0.106	13	$CeO_2(s) + Sm_2O_3(s)$	1.3
$LuVO_3$	mp-769783	None	0.01	T, F, F	0.013	8	$Lu_2O_3(s) + V_2O_3(s)$	1.6
$HoCrO_3$	mp-769818	None	0.00	T, F, F	0.000	10	$HoCrO_3(s)$	2.5
$TbCrO_3$	mp-769920	None	0.00	T, F, F	0.000	9	$TbCrO_3(s)$	2.5
NdCuTeO	mp-974307	416523	0.02	T, F, F	0.069	11	$Cu(s) + Nd_2TeO_2(s) + HTe^-$	1.4
ErZnAsO	mp-983603	420203	0.00	T, T, F	0.018	10	$Zn_3As_2(s) + Er_2O_3(s) + ZnAs_2(s)$	1.6

Supplementary Table 2: The screening strategy described in this work was applied to the 45 materials compositions of photocathodes reported in the literature. See main text for the list of the materials compositions as well as references where successful CO_2 reduction has been demonstrated from these materials. The properties of materials which pass tier 3 are listed in this table. Materials which pass the subsequent tiers 4, 5 and 6 are also noted in the table. The chemical formula (Formula), MP material ids (mp-id), ICSD-id (if any), number of atoms in the unit cell, n_{sites}, and the energy above hull (ΔE^{hull} in eV/atom) is shown for all experimentally known photocathode materials which pass tier 3. Materials which have the Gibbs free energy with respect to Pourbaix stable phases, $\Delta G_{\mathrm{pbx}} < 0.2$ eV/atom at any pH between 0 and 14 and have at least one solid species as a decomposition product are noted under the Aq column in the table. The aqueous stability is noted as T (for True) and F (for False) for -0.5 V, -1.0 V and -1.5 V vs RHE, as comma separated symbols. The ΔG_{pbx} in eV/atom, pH and decomposed species for the most negative potential which satisfies the criteria for aqueous stability are also listed. The bandgaps computed from PBE or PBE+U ($E^{\mathrm{g}}_{\mathrm{PBE}}$ in eV) and from HSE06 ($E^{\mathrm{g}}_{\mathrm{HSE06}}$ in eV) are also noted in the table. The HSE06 conduction band minima energy in eV is shown for the surfaces of the materials. See Supplementary Table 1 for the indices of the planes. The last column shows if the material passes tier 3, 4, 5, 6 as four comma-separated Y (for yes) or N (for no).

Formula	mp-id	ICSD-id	ΔE^{hull}	Aq	ΔG_{pbx}	pH	Decomp	$E^{\mathrm{g}}_{\mathrm{PBE}}$	$E^{\mathrm{g}}_{\mathrm{HSE06}}$	Band Edges	n_{sites}	Tier 3,4,5,6
$MoSe_2$	mp-7581	16948	0.00	T, F, F	0.08	3	Mo(s) + H_2Se(aq)	1.4	1.9	-3.56, -4.41, -3.18	3	Y, Y, Y, Y
$MoSe_2$	mp-1018807	644346	0.00	T, F, F	0.08	3	Mo(s) + H_2Se(aq)	1.3	1.9	-3.58	6	Y, Y, Y, Y
$MoSe_2$	mp-1634	49800	0.00	T, F, F	0.08	3	Mo(s) + H_2Se(aq)	1.4	1.9	-3.81	6	Y, Y, Y, Y
WSe_2	mp-1821	40752	0.00	T, F, F	0.19	3	W(s) + H_2Se(aq)	1.45	1.9	-3.52	6	Y, Y, Y, Y
ZnTe	mp-571195	80076	0.00	T, T, F	0.00	0	ZnTe(s)	1.1	2	-3.53	6	Y, Y, Y, Y
ZnTe	mp-2176	41984	0.00	T, T, F	0.00	0	ZnTe(s)	1.1	2.1	-4.62, -3.31	2	Y, Y, Y, Y
ZnTe	mp-8884	67779	0.01	T, T, F	0.01	0	ZnTe(s)	1.1	2.1	-3.33, -3.81	4	Y, Y, Y, Y
ZnSe	mp-1190	652224	0.00	T, F, F	0.00	6	ZnSe(s)	1.17	2.3	-3.775	2	Y, Y, Y, Y
ZnSe	mp-380	652217	0.0	T, F, F	0.01	6	ZnSe(s)	1.20	2.3		4	Y, Y, Y, N
$NaTaO_3$	mp-4170	88378	0.01	T, T, F	0.15	14	$NaTa_4O_8$(s) + Na^+	2.3	3.1	-3.14	5	Y, Y, Y, Y
$SrTiO_3$	mp-5229	80872	0.00	T, T, F	0.16	14	Ti_2O_3(s) + $SrOH^+$	2.1	3.2	-2.99	5	Y, Y, Y, Y
TiO_2	mp-2657	202240	0.04	T, F, F	0.11	4	TiO_2(s)	1.8	3.2	-4.22	6	Y, Y, Y, Y
$SrTiO_3$	mp-4651	182248	0.00	T, T, F	0.16	14	Ti_2O_3(s) + $SrOH^+$	1.8	3.3	-1.89	10	Y, Y, Y, N
TiO_2	mvc-6590	None	0.04	T, F, F	0.12	4	TiO_2(s)	1.9	3.3	-4.34	12	Y, Y, Y, N
CeO_2	mp-20194	164225	0.00	T, T, F	0.00	13	CeO_2(s)	1.9	3.5	-2, -3.55	3	Y, Y, Y, Y
TiO_2	mp-34688	None	0.01	T, T, F	0.17	14	Ti_2O_3(s)	2.0	3.5	-3.69	6	Y, Y, Y, Y
TiO_2	mp-390	202242	0.01	T, T, F	0.15	14	Ti_2O_3(s)	2.1	3.5	-3.49	6	Y, Y, Y, Y
TiO_2	mp-766454	None	0.06	T, F, F	0.18	4	TiO_2(s)	2.3	3.7		9	Y, Y, N, N
TiO_2	mp-430	154035	0.07	T, F, F	0.20	4	TiO_2(s)	2.2	3.7		12	Y, Y, N, N
$NaTaO_3$	mp-4675	88377	0.00	T, T, F	0.12	14	$NaTa_4O_8$(s) + Na^+	2.3	3.7		10	Y, Y, N, N
$NaTaO_3$	mp-4699	88376	0.00	T, T, F	0.11	14	$NaTa_4O_8$(s) + Na^+	2.4	3.7		20	Y, Y, N, N
Ga_2O_3	mp-886	34243	0.00	T, F, F	0.00	5	Ga_2O_3(s)	2.0	3.8		10	Y, Y, N, N
TiO_2	mp-9173	75179	0.06	T, F, F	0.18	4	TiO_2(s)	2.5	4		12	Y, Y, N, N
$Zn(GaO_2)_2$	mp-5794	81106	0.00	T, F, F	0.00	7	$Zn(GaO_2)_2$(s)	2.3	4.1		14	Y, Y, N, N
Ga_2O_3	mp-1243	166199	0.03	T, F, F	0.07	5	Ga_2O_3(s)	2.4	4.2		10	Y, Y, N, N
Ta_2O_5	mp-10390	280396	0.01	T, F, F	0.03	7	Ta_2O_5(s)	1.2			14	Y, Y, N, N
$SrTiO_3$	mp-776018	None	0.04	T, F, F	0.19	14	TiO_2(s) + Sr^{2+}	1.7			30	Y, N, N, N
TiO_2	mp-754769	None	0.05	T, F, F	0.16	4	TiO_2(s)	2.3			24	Y, N, N, N
TiO_2	mp-775938	None	0.04	T, F, F	0.12	4	TiO_2(s)	2.1			36	Y, N, N, N
Si	mp-971662	None	0.06	F, F, T	0.06	0	Si(s)	1.3			46	Y, N, N, N

Supplementary Table 3: List of references where successful synthesis of the identified photocathodes has been reported. The chemical formula (Formula), the MP material-id (mp-id) for the identified photocathodes which have an ICSD-id. If the material has been used for CO_2 reduction before it is noted as Y in the "Experimentally Tested" column and N otherwise. The reference where a recipe for the synthesis of the materials can be found or a reference with demonstrated CO_2 reduction is listed in the last column. Note that wurtzite-AlAs structure has an ICSD-id which directs to a computational study. The wurtzite-AlAs has no reported synthesis.

Formula	mp-id	ICSD-id	Experimentally Tested	Reference
TiO_2	mp-2657	202240	Y	1
TiO_2	mp-390	202242	Y	[2]
$SrTiO_3$	mp-5229	80872	Y	[3]
$NaTaO_3$	mp-4170	88378	Y	[4]
Ta_2SnO_6	mp-556489	54078	N	[5]
CeO_2	mp-20194	164225	Y	[6]
Ca_4As_2O	mp-8789	68203	N	[7]
AlAs	mp-2172	606009	N	[8]
AlAs	mp-8881	67771	N	None
SiAs	mp-1863	43227	N	[9]
Cd_2AsCl_2	mp-27776	26013	N	[10]
$MgSiAs_2$	mp-1016197	182367	N	[11]
LiMgAs	mp-12558	107954	N	[12]
CdHgAsBr	mp-569454	240354	N	[13]
AlSb	mp-2624	609288	N	[14]
$Rb_3Sb_2Au_3$	mp-9274	78978	N	[15]
$MoSe_2$	mp-1634	49800	Y	[16]
$MoSe_2$	mp-1018807	644346	N	[17]
$MoSe_2$	mp-7581	16948	N	[18]
WSe_2	mp-1821	40752	Y	[16]
ZnSe	mp-1190	652224	Y	[19]
$Zn(GaSe_2)_2$	mp-15776	168594	N	[20]
$CsYZnSe_3$	mp-574620	280847	N	[21]
$CsGaSe_3$	mp-510283	98670	N	[22]
GaSe	mp-1943	63122	N	[23]
GaSe	mp-11342	73388	N	[24]
Ga_2Se_3	mp-1340	37168	N	[25]
Ga_2TeSe_2	mp-28423	64617	N	[26]
$Cs_2Cd_3Te_4$	mp-567386	90369	N	[27]
$CsLaCdTe_3$	mp-12491	173316	N	[28]
$LiInTe_2$	mp-20782	639906	N	[29]
$RbInTe_2$	mp-22255	75346	N	[30]
RbAuTe	mp-9008	71652	N	[31]
ZnTe	mp-571195	80076	N	[32]
ZnTe	mp-8884	67779	N	[33]
ZnTe	mp-2176	41984	Y	[34]
$Zn(GaTe_2)_2$	mp-15777	44888	N	[35]
$Cd(GaTe_2)_2$	mp-13949	25646	N	[36]
GaTe	mp-542812	153456	N	[37]
YbTe	mp-1779	653185	N	[38]
GaTeCl	mp-27449	15582	N	[39]
InTeBr	mp-29236	100705	N	[40]
$Hg_3(TeBr)_2$	mp-27853	27402	N	[41]
$ZrVF_6$	mp-557686	73354	N	[42]

Supplementary Notes

Supplementary Note 1

Phonon Spectra of the Hypothetical Identified Photocathodes: Supplementary Figure 1 shows the phonon spectra of the eight hypothetical photocathodes identified in this work. The Γ-point frequency calculations, performed using density-functional perturbation theory simulations, showed that $BeSiAs_2$ (mp-1009087), $CeThO_4$ (mp-36734), BiTeBr (mp-33723), NaCaAs (mp-961685) and TiO_2 (mp-34688) exhibit small negative frequencies (at most -0.4 cm^{-1}) for the acoustic modes close to the gamma point. For these five materials all other normal modes have positive frequencies indicating that they maybe dynamically stable. We further computed the phonon dispersion along the high-symmetry paths in the Brillouin zone using the finite difference method on their 3×3×3 supercell, see Figure 1 (a)-(e). The number of atoms for the force displacement simulations ranged from 81 to 216 and the total number of displacement simulations per material ranged from 3 to 18. $BeSiAs_2$, $CeThO_4$, BiTeBr and NaCaAs are found to be stable throughout the Brillouin zone. Small imaginary phonon modes were observed for the monoclinic TiO_2 phase along the Γ-Y/X/N/M direction which indicates a lattice instability.

The remaining three materials, $SrIn_2Te_4$ (mp-35663), VOF with material-id as mp-768037 and VOF with material-id as mp-764309 have significantly more imaginary acoustic modes at the Γ point, up to -43 cm^{-1}, -25 cm^{-1} and -228 cm^{-1}, respectively. The phonon spectra of these three materials obtained from the single unit-cell simulations of these materials are shown in Figure 1 (f)-(h). These three materials are clearly dynamically unstable.

Supplementary Note 2

Relation between HSE06 and PBE/PBE+U bandgaps: Supplementary Figure 2 compares the bandgaps computed from HSE06, $E^{\mathrm{g}}_{\mathrm{HSE06}}$, with the gaps computed from PBE or PBE+U functional, $E^{\mathrm{g}}_{\mathrm{PBE}}$. The $E^{\mathrm{g}}_{\mathrm{HSE06}} = 1.6\ \times E^{\mathrm{g}}_{\mathrm{PBE}}$ relation fits the data for the 205 materials with

only six outliers beyond the 95 % prediction bounds. This is particularly remarkable since the data is fit to 205 materials which have diverse crystal structures and materials compositions. The outliers are Ti_2FeO_5 (mp-31857), $TiFeO_3$ (mp-19417), $TiFeO_3$ (mp-19270), $SrLiNiF_6$ (mp-559663), and $CaTi_4(FeO_4)_3$ (mvc-11639) and warrant further careful investigation.

The HSE06 bandgap simulations did not converge for 30 of the 235 compounds passed to tier 5. These materials included Ta_2O_5, $GdCrO_3$, $YCrO_3$, YVO_3 and all materials containing Nd, Sm, Lu, Dy, Er, Ho, Sm, or Tb. The convergence problem with lanthanides and actinites, conceivably due to strong electron correlation in these materials, has been noted before.[43]

Supplementary Methods

Note that default MP simulations settings were used for computing the phonon spectra, albeit with an increased cutoff energy, 600 eV instead of the default 520 eV, and a high k-point density. Also, the forces on atoms were relaxed to 0.001 eV/Å. A total energy convergence of 1 meV was achieved with a $3 \times 3 \times 3$ k-point grid for the $3 \times 3 \times 3$ supercell calculations. For the Γ-point simulations, the same parameters were used except the k-point density was at least 65×the magnitude of the reciprocal lattice vectors. Phonon calculations were performed with PHONOPY.[44]

Supplementary References

(1) Liu, L., Zhao, H., Andino, J. M. & Li, Y. Photocatalytic CO_2 reduction with H_2O on TiO_2 nanocrystals: Comparison of anatase, rutile, and brookite polymorphs and exploration of surface chemistry. *ACS Catal.* **2**, 1817–1828 (2012).

(2) Ni, M., Leung, M. K., Leung, D. Y. & Sumathy, K. A review and recent developments

in photocatalytic water-splitting using TiO_2 for hydrogen production. *Renew. Sust. Energy Rev.* **11**, 401–425 (2007).

(3) Zeng, S., Kar, P., Thakur, U. K. & Shankar, K. A review on photocatalytic CO_2 reduction using perovskite oxide nanomaterials. *Nanotechnology* **29**, 052001 (2018).

(4) Li, M. *et al.* Highly efficient and stable photocatalytic reduction of CO_2 to CH_4 over Ru loaded $NaTaO_3$. *Chem. Commun.* **51**, 7645–7648 (2015).

(5) Mizoguchi, H., Sleight, A. W. & Subramanian, M. A. Low temperature synthesis and characterization of $SnTa_2O_6$. *Mater. Res. Bull.* **44**, 1022–1024 (2009).

(6) Singh, P. & Hegde, M. S. $Ce_{1-x}Ru_xO_{2-\delta}$ (x=0.05, 0.10): A new high oxygen storage material and Pt, Pd-free three-way catalyst. *Chem. Mater.* **21**, 3337–3345 (2009).

(7) Hadenfeldt, C. & Vollert, H. Darstellung und kristallstruktur der calciumpnictidoxide Ca_4P_2O und Ca_4As_2O. *J. Less-Common Met.* **144**, 143–151 (1988).

(8) Ettenberg, M. & Paff, R. Thermal expansion of AlAs. *J. Appl. Phys.* **41**, 3926–3927 (1970).

(9) Wadsten, T. The crystal structure of SiAs. *Acta Chem. Scand* **19** (1965).

(10) Rebbah, A., Yazbeck, J., Leclaire, A. & Deschanvres, A. Structure du dichlorure d'arsenic et de dicadmium. *Acta Crystallogr. B-Stru.* **36**, 771–773 (1980).

(11) Spring-Thorpe, A. & Pamplin, B. Growth of some single crystal II–IV–V_2 semiconducting compounds. *J. Cryst. Growth* **3**, 313–316 (1968).

(12) Beleanu, A. *et al.* Systematical, experimental investigations on LiMgZ (Z= P, As, Sb) wide band gap semiconductors. *J Phys. D Appl. Phys.* **44**, 475302 (2011).

(13) Zou, J.-P. *et al.* Synthesis, crystal and band structures, and optical properties of a new quaternary metal pnictidehalide: ($Hg_2Cd_2As_2Br$) Br. *Inorg. Chem.* **45**, 6365–6369 (2006).

(14) Sonomura, H., Nishimura, T. & Miyauchi, T. Lattice constants in the $Al_xGa_{1-x}Sb$ system. *Phys. Status Solidi* **61**, K51–K53 (1980).

(15) Müller, J. & Zachwieja, U. Verbindungen in den systemen kalium (rubidium)/gold/antimon: $K_3Au_3Sb_2$, $Rb_3Au_3Sb_2$ und $K_{1,74}Rb_{0,26}RbAu_3Sb_2$. *Z. Anorg. Allg. Chem.* **622**, 635–639 (1996).

(16) Asadi, M. *et al.* Nanostructured transition metal dichalcogenide electrocatalysts for CO_2 reduction in ionic liquid. *Science* **353**, 467–470 (2016).

(17) Oswald, H., Asper, R. & Lieth, R. Preparation and crystal growth of materials with layered structures. *Reidel, Dordrecht* 122 (1977).

(18) Towle, L. C., Oberbeck, V., Brown, B. E. & Stajdohar, R. E. Molybdenum diselenide: rhombohedral high pressure-high temperature polymorph. *Science* **154**, 895–896 (1966).

(19) Kuehnel, M. F. *et al.* ZnSe quantum dots modified with a Ni (cyclam) catalyst for efficient visible-light driven CO_2 reduction in water. *Chem. Sci.* **9**, 2501–2509 (2018).

(20) Hahn, H., Frank, G., Klingler, W., Störger, A. D. & Störger, G. Untersuchungen über ternäre chalkogenide. VI. über ternäre chalkogenide des aluminiums, galliums und indiums mit zink, cadmium und quecksilber. *Z. Anorg. Allg. Chem.* **279**, 241–270 (1955).

(21) Mitchell, K., Haynes, C. L., McFarland, A. D., Van Duyne, R. P. & Ibers, J. A. Tuning of optical band gaps: syntheses, structures, magnetic properties, and optical properties

of $CsLnZnSe_3$ (Ln= Sm, Tb, Dy, Ho, Er, Tm, Yb, and Y). *Inorg. Chem.* **41**, 1199–1204 (2002).

(22) Do, J. & Kanatzidis, M. G. The one-dimensional polyselenide compound $CsGaSe_3$. *Z. Anorg. Allg. Chem.* **629**, 621–624 (2003).

(23) Arancia, G., Grandolfo, M., Manfredotti, C. & Rizzo, A. Electron diffraction study of melt-and vapour-grown $GaSe_{1-x}S_x$ single crystals. *Phys. Status Solidi* **33**, 563–571 (1976).

(24) Ider, M., Pankajavalli, R., Zhuang, W., Shen, J. & Anderson, T. Thermochemistry of the Ga-Se system. *ECS J. Solid State Sc.* **4**, Q51–Q60 (2015).

(25) Ghémard, G., Jaulmes, S., Etienne, J. & Flahaut, J. Structure de la phase ordonnee du sesquiseleniure de gallium, Ga_2Se_3. *Acta Crystallogr. C* **39**, 968–971 (1983).

(26) Bredol, M. & Leute, V. The crystal structure of Ga_2Se_2Te. *Phys. Status Solidi* **107** (1988).

(27) Narducci, A. A. & Ibers, J. A. Syntheses, crystal structures, and band gaps of $Cs_2Cd_3Te_4$ and $Rb_2Cd_3Te_4$. *J. Alloy. Compd.* **306**, 170–174 (2000).

(28) Liu, Y., Chen, L., Wu, L.-M., Chan, G. H. & Van Duyne, R. P. Syntheses, crystal and band structures, and magnetic and optical properties of new $CsLnCdTe_3$ (Ln= La, Pr, Nd, Sm, Gd- Tm, and Lu). *Inorg. Chem.* **47**, 855–862 (2008).

(29) Kühn, G., Schumann, B., Oppermann, D., Neumann, H. & Sobotta, H. Preparation, structure, and infrared lattice vibrations of $LiInTe_2$. *Z. Anorg. Allg. Chem.* **531**, 61–66 (1985).

(30) Dhingra, S. S. & Haushalter, R. C. One dimensional inorganic polymers: synthesis and structural characterization of the main group metal polymers $K_2HgSnTe_4$, $(Et_4N)_2HgSnTe_4$, $(Ph_4P)GeInTe_4$, and $RbInTe_2$. *Chem Mater.* **6**, 2376–2381 (1994).

(31) Bronger, W. & Kathage, H. Zur synthese und kristallstruktur von alkalimetallgold-chalkogeniden AAuX mit A = Na, K, Rb oder Cs und X = S, Se oder Te. *J. Alloy. Compd.* **184**, 87–94 (1992).

(32) Kusaba, K. & Weidner, D. J. Structure of high pressure phase I in ZnTe. In *AIP Conf. Proc.*, vol. 309, 553–556 (AIP, 1994).

(33) Kumar, V., Kumar, V. & Dwivedi, D. Growth and characterization of zinc telluride thin films for photovoltaic applications. *Phys. Scripta* **86**, 015604 (2012).

(34) Jang, J.-W. *et al.* Aqueous-solution route to zinc telluride films for application to CO_2 reduction. *Angew. Chem.* **53**, 5852–5857 (2014).

(35) Woolley, J. & Ray, B. Effects of solid solution of Ga_2Te_3 with $A^{II}B^{VI}$ tellurides. *J. Phys. Chem. Solids* **16**, 102–106 (1960).

(36) Neumann, H., Moise, E., Schwer, H. & Krämer, V. Infrared lattice vibrations of $CdGa_2Te_4$. *Cryst. Res. Technol.* **28**, 635–639 (1993).

(37) Antonopoulos, J., Karakostas, T., Bleris, G. & Economou, N. On the phase diagram of the Ga-Te system in the composition range 55 at% Te. *J. Mater. Sci.* **16**, 733–738 (1981).

(38) Chatterjee, A., Singh, A. K. & Jayaraman, A. Pressure-induced electronic collapse and structural changes in rare-earth monochalcogenides. *Phy. Rev. B* **6**, 2285–2291 (1972).

(39) Kniep, R., Wilms, A. & Beister, H. J. Phase relations in Ga_2X_3-GaY_3 systems (X= Se, Te; Y= Cl, Br, I) - crystal growth, structural relations and optical absorption of intermediate compounds GaXY. *Mater. Res. Bulletin* **18**, 615–620 (1983).

(40) Kniep, R. & Wilms, A. Phase relations in the $InBr_3$-In_2Te_3 system and the crystal structure of InTeBr. *Mater. Res. Bull.* **15**, 763–770 (1980).

(41) Puff, H. & Küster, J. Die kristallstruktur der kubischen triquecksilber-dichalkonium-dihalogenide. *Naturwissenschaften* **49**, 464–465 (1962).

(42) Le Mercier, T., Chassaing, J., Bizot, D. & Quarton, M. Structural, spectroscopic and magnetic studies of $V^{II}M^{IV}F_6$ compounds with M^{IV}= Zr, Nb. *Mater. Res. Bull.* **27**, 259–267 (1992).

(43) Perdew, J. P., Burke, K. & Ernzerhof, M. Generalized gradient approximation made simple. *Phys. Rev. Lett.* **77**, 3865 (1996).

(44) Togo, A. & Tanaka, I. First principles phonon calculations in materials science. *Scr. Mater.* **108**, 1–5 (2015).
